# Finding the perfect promoter for Cas9 in homing gene drives using single cell transcriptome data

**DOI:** 10.1038/s41467-026-76107-0

**Published:** 2026-07-25

**Authors:** Yingke Wu, Yunchen Xia, Ziyin Yao, Weizhe Chen, Xihua Jia, Nan Liang, Jackson Champer

**Affiliations:** 1 Center for Bioinformatics, School of Life Sciences, Center for Life Sciences, Peking University, Beijing, China; 2 PTN program, School of Life Sciences, Tsinghua University, Beijing, China; 3 State Key Laboratory of Gene Function and Modulation Research, School of Life Sciences, Biomedical Pioneering Innovative Center and Beijing Advanced Innovation Center for Genomics, Peking University, Beijing, China

**Keywords:** CRISPR-Cas9 genome editing, Gene expression, Genetic engineering

## Abstract

Gene drive can modify or suppress vector populations by spreading drive alleles. In CRISPR homing drives, regulating Cas9 expression has been effective for improving drive performance, but selecting suitable promoters is often a major challenge. Here, we evaluate 35 Cas9 constructs with distinct promoters in *Drosophila melanogaster* and identify associations between drive performance and single-cell RNA expression patterns of the promoter-associated genes. Our results indicate that higher drive conversion is associated with elevated expression of the promoter-associated gene in reproductive cells, but embryo resistance allele formation correlates with excessive female germline expression. For males, early germline expression produces superior performance. Thus, optimal drive performance requires restricting Cas9 expression to a tight quantitative and spatiotemporal window. Additionally, we find that an in situ construct significantly reduces potentially harmful somatic expression. Based on these results, we propose criteria for selecting promoters, providing a rationale and guidance for optimization of homing gene drives.

## Abstract (web summary)

CRISPR homing gene drives can help control disease vectors but require precise Cas9 expression. Here, the drive performance of 35 Cas9 constructs with different promoters in Drosophila were compared to single cell RNA expression patterns of promoter-associated genes.

## Introduction

Gene drive is a genetic manipulation technology that leverages the characteristics of selfish genetic elements and modern gene-editing tools. It enables control of pathogens or suppression of vector populations by promoting the spread of drive alleles though populations by super-Mendelian inheritance^1–5^. Among various drive approaches, CRISPR based homing gene drives are the most extensively studied and powerful. In this system, Cas9 and one or more guide RNAs (gRNAs) cleave the wild-type allele in germline cells of heterozygotes, converted them into drive alleles and thereby increasing the drive inheritance probability beyond Mendelian expectations (though recent studies have also showed that others nucleases such as Cas12a can function efficiently^6^). The feasibility of CRISPR based homing gene drive has been demonstrated in a variety of model organisms, disease vectors, invasive species, and agricultural pests including *Drosophila melanogaster*^6–9^, *Drosophila suzukii*^10^, *Ceratitis capitata*^11^, *Saccharomyces cerevisiae*^12,13^, *Candida albicans*^14^, *Plutella xylostella*^15^, *Anopheles gambiae*^16–18^, *Anopheles stephensi*^19,20^, *Aedes aegypti*^21^, *Culex quinquefasciatus*^22^, *Mus musculus*^23^, and viral populations^24,25^. However, none of these systems have yet been deployed in the wild. This is in large part due to issues in overall drive effectiveness. Developing drives that function well even in laboratory cage populations has been challenging, at least in multicellular organisms.

Gene drive performance can be evaluated from three aspects^26^: the drive conversion rate, the formation of resistant alleles, and fitness costs. The drive conversion rate is a core parameter for assessing the propagation potential of the drive element within a population. It primarily reflects the efficiency with which Cas9 cleaves the target DNA in germline cells and precisely copies the drive element through homology-directed repair (HDR). A high conversion rate enhances deviation from Mendelian inheritance ratios, thereby accelerating the spread of the drive element throughout the population^5^. This can allow a modification drive to spread despite fitness costs. For suppression drives, it is even more critical, directly affecting suppressive power.

Resistance alleles arise from the end-joining repair pathway or (more rarely) incomplete HDR, changing the wild-type sequence and thereby rendering them resistant to CRISPR-mediated cleavage, which interferes with drive spread^27,28^. Resistance alleles are classified into functional and nonfunctional resistance alleles^29,30^. Functional resistance alleles usually result from small indels at or near the Cas9 cut site that mutate the PAM or gRNA seed region, preventing cleavage while largely preserving the encoded protein. Such alleles can cause major drive failure, but they can be effectively overcome by gRNA multiplexing, though this can come at the cost of a modest decline in drive efficiency if many gRNAs are required^29,31^. By contrast, more common nonfunctional alleles remain problematic. They arise from frameshifts or more impactful indels that destroy protein function. All these alleles primarily form during the germline or later during embryonic stages^29,32^. In the germline, Cas9 can convert wild-type alleles into germline resistance alleles following DNA cleavage, enabling these resistance alleles to be heritable within the population^7^. During embryonic development, maternally deposited Cas9 and gRNA can mediate DNA cleavage and repair in the zygote or early embryo, resulting in the formation of embryo resistance alleles^19^. Resistance alleles formed during the zygote stage affects all cells in the developing organism, but it is also possible for resistance alleles to form later in either all or just a subset of cells. In both these cases (though usually not in the latter^33^), they can be part of the germline, potentially blocking drive conversion while still being inherited by the next generation. However, some only arise within somatic cells during embryogenesis and remain confined at the individual level, unable to enter the germline or be effectively transmitted to offspring^34^. These resistance alleles can still have similar negative fitness effects like somatic Cas9 activity (see below). Overall, high rates of resistance allele formation pose a major barrier to the successful spread of gene drives within populations, particularly when the drive system carries fitness cost^7,17,35^.

Fitness costs can come from a variety of sources, in particular somatic expression in suppression drives^29,36^. An ideal homing gene drive should ensure that Cas9 expression is restricted to germline cells associated with gametogenesis, but “leaky” somatic expression can occur even with a mostly germline-specific promoter^37^. This reduction in fitness reduces survival and/or reproductive competitiveness, ultimately affecting the efficiency of the drive^29,38^. Aberrant expression of Cas9 in somatic tissues may lead to reduced fitness if the wild-type allele (which is destroyed by somatic expression, regardless of whether HDR or end-joining occurs) is required for survival or fertility. Thus, this fitness cost due to somatic activity is particularly problematic for suppression gene drives^17,30,38^. Homing suppression drives rely on the drive reaching high frequency within the population to generate a sufficient proportion of homozygous individuals that are sterile or nonviable, thereby achieving effective population suppression (high “suppressive power”). However, if drive/wild-type heterozygous individuals carrying the drive suffer fitness cost due to somatic activity, the drive will spread to a lower proportion of the population when it reaches equilibrium, potentially preventing population elimination^38–40^. Additional fitness costs may result from the burden or toxicity of transgenes, Cas9 off-target effects, and insertion-site position effects^41^.

The Cas9 promoter plays a critical role in determining the drive performance^37^. To date, multiple Cas9 promoters with different drive performance have been identified in *Drosophila melanogaster*. For example, the *nanos* promoter exhibits a relatively high drive conversion rate and embryo resistance rate, while maintaining low levels of leaky somatic expression^7^. In contrast, the *CG4415* promoter achieves a similarly high drive conversion rate while effectively reducing the embryo resistance rate^37^. However, it also maintains low levels of somatic expression, higher than those observed with the *nanos* promoter. In *Anopheles gambiae*, the *nanos* and *zpg* promoters also have high drive conversion efficiency and low embryo resistance, while still retaining some somatic expression that causes fitness costs in suppression drives^38,39,42^. Although previous studies have revealed that different promoters exhibit distinct performance in gene drive systems^37^, the regulatory mechanisms by which Cas9 promoters influence drive efficiency at the transcriptomic level remain unclear, and systematic efforts to screen for high-performance promoters remain limited. This challenge is particularly evident in non-model species with limited foundational research^10,11,21,22,43^, where Cas9 promoters are typically sourced from those validated in model organisms or identified by cloning the upstream regions of highly expressed reproductive tissue genes using RNA-seq data. However, these approaches have disadvantages. On the one hand, the expression levels and spatiotemporal patterns of promoters transferred across species may not always align with expectations^20,37^. On the other hand, RNA-seq screening only reflects the overall average expression levels at the tissue level, failing to resolve the fine-scale expression patterns of genes across different cell types and developmental stages within complex reproductive tissues, where critical germline cells represent only a fraction of the total cells that are assessed.

With the increasing availability of high-resolution transcriptomic resources, including the recently published single-nucleus atlas of *Aedes aegypti*^44^, these datasets can potentially address this issue by providing precise information on expression patterns. Yet, it remains unclear exactly which expression pattern may be optimal, or to what extent expression patterns inferred from transcriptomic data reflect the actual Cas9 activity driven by promoters derived from native genes. It has been suggested that efficient homing may depend on Cas9 activity being restricted to specific germline cell stages in which homology-directed repair is favored over end joining^45,46^. Therefore, it is crucial to further investigate the relationship between expression of candidate promoter genes and drive performance to develop efficient and broadly applicable Cas9 promoter screening strategies for diverse species.

Based on this, our study systematically tests 35 Cas9 expression constructs driven by different promoters in several different types of gene drive systems in *Drosophila melanogaster*. By comparing their performance and integrating single-cell transcriptomic data^47^, we identify potential correlations between drive performance and gene expression patterns. While high expression in specific reproductive cells is necessary for efficient drive conversion, excessive or ectopic expression generates undesirable resistance or somatic activity. This leads to our central conclusion that optimal performance requires confining Cas9 expression to a tight quantitative and spatiotemporal window. Our analysis enables us to define the key criteria for selecting high performance promoters, providing a foundation and practical strategies for optimization of nuclease promoters in homing drives.

## Results

### Selection of 35 promoter-candidate genes with different expression patterns

**Fig. 1:**
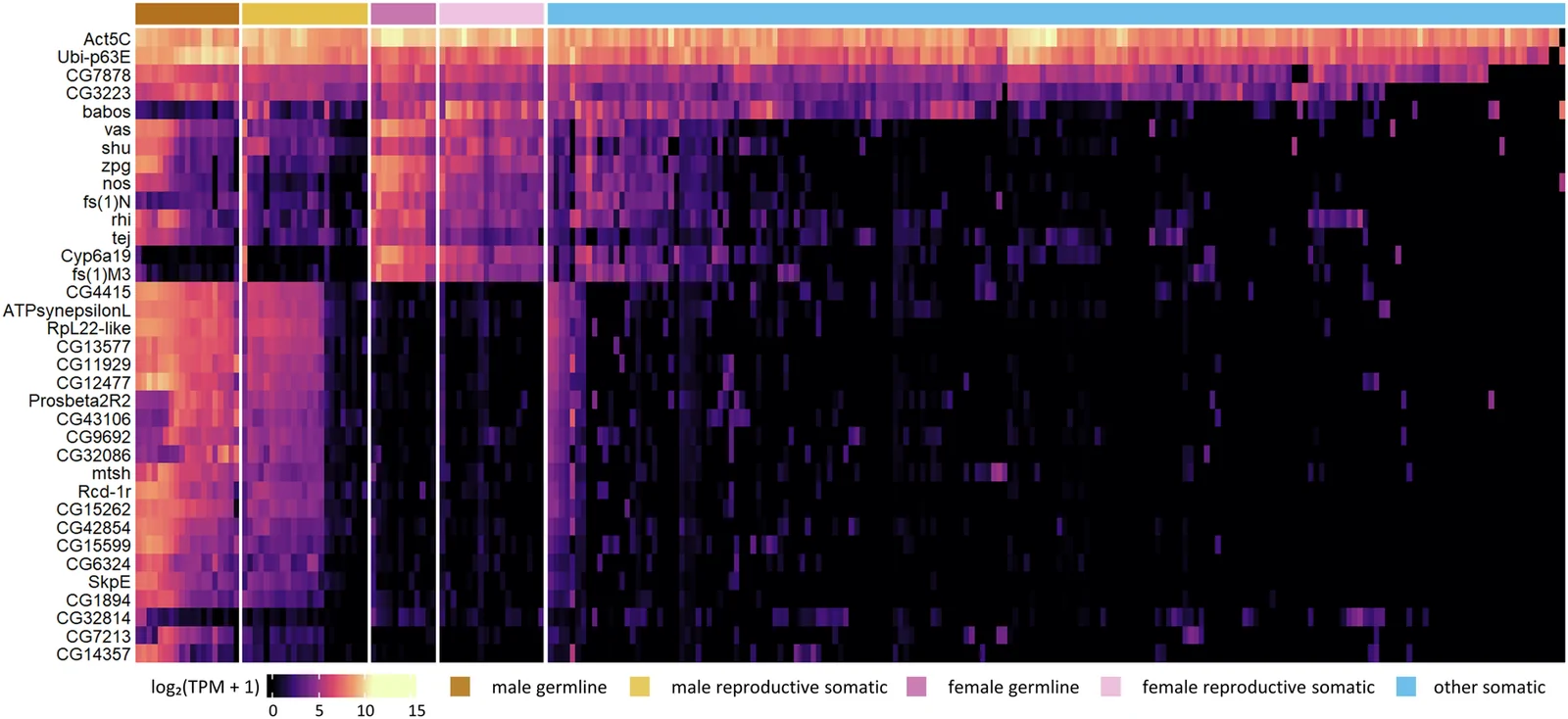
Single cell expression profiles of promoter candidate genes in *Drosophila melanogaster.* The heatmap displays the expression profiles of 35 genes across 259 single cell subtypes from the Fly Cell Atlas. Raw Transcripts Per Million (TPM) were transformed to log_2_(TPM + 1) and color-mapped. To determine gene and cell type order, we computed Euclidean distances on the transformed expression matrix and performed complete-linkage hierarchical clustering.

Based on single-cell transcriptomic data in the Fly Cell Atlas^47^, we selected 25 genes sex-specific or both-sex germline expression chosen to minimize somatic expression (Fig. 1, and Fig. S1). We included eight additional germline promoters from previous studies^37^ and added two constitutive genes (*Act5C* and *Ubi-p63E*) that are expressed at relatively high levels across various cell types and conditions. The promoter regions were defined as the 5′ untranslated region (5′ UTR) together with 64 to 3382 base pairs upstream of these genes (Table S1), selected according to transcription factor binding densities from the ReMap database (version 2022)^48^ while attempting to avoid regions in the coding sequence and UTRs of adjacent genes.

### Different promoters exhibit substantially varied drive performance

**Fig. 2:**
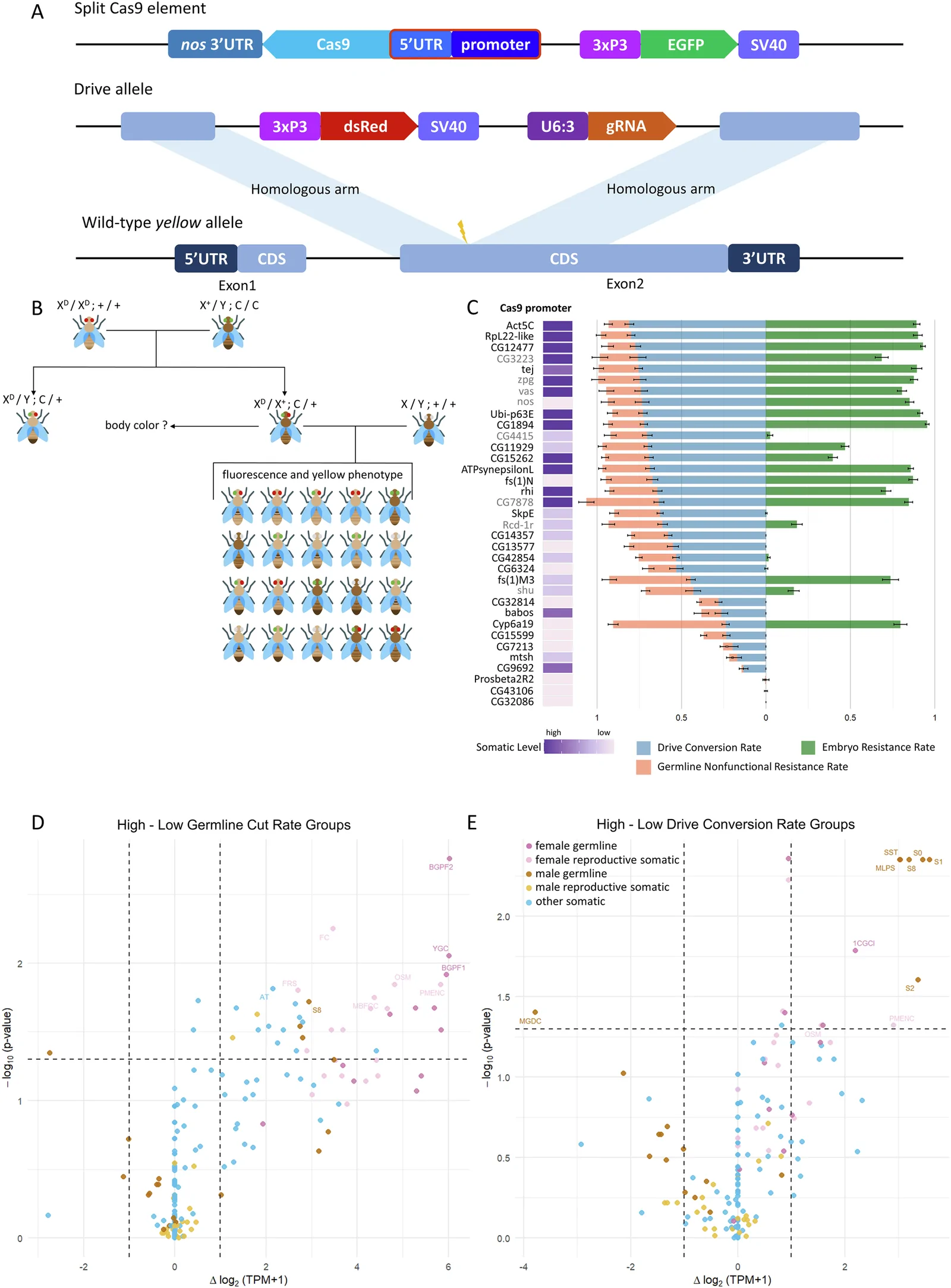
Performance of Cas9 promoters with the split drive targeting *yellow.* **A** The split Cas9 element expresses Cas9 under the control of different promoters and germline the *nanos* 3′ UTR. The gRNA element targets *yellow*. **B** Females with the drive element are first crossed to males with Cas9. Female progeny carrying both fluorescent markers were phenotyped for body color as an indicator of somatic Cas9 activity. They were then crossed with *w*^*1118*^ males, and their offspring were phenotyped. **C** Drive performance of 35 Cas9 constructs with promoters. The results for *rhi* were obtained using the RO3 construct. Data from gray labeled Cas9 promoters are from a previous study^37^. For improved visualization, drive conversion rates calculated as slightly below zero are displayed as zero. Error bars indicate standard error of the mean. Fig. S1 presents the germline cell expression profiles of the promoter associated genes, and Fig. S2 shows the relationship between the phenotype classes and the different drive parameters (the purple color in this figure corresponds to the purple colors for somatic expression in Fig. S2). Source data are provided in Source Data S1-S2. **D** Differential expression of promoter-associated genes between high (greater than 90%) and low (less than 25%) germline cut rate (drive conversion + germline nonfunctional resistance) groups. Each point represents a single cell type from the Fly Cell Atlas dataset, with the horizontal axis indicating the difference in log_2_(TPM + 1) between high and low cut rate groups, and the vertical axis showing -log_10_(*p* value) derived from two-sided Wilcoxon rank-sum tests. No adjustment was made for multiple comparisons. The horizontal dashed line indicates the threshold for *p* < 0.05, and the two vertical dashed lines indicate the thresholds for |Δlog2(TPM + 1)| > 1. Top cell types with significant differences (*p* < 0.05 and |Δlog_2_(TPM + 1)| > 1) are labeled with abbreviated names (Table S2). Exact *p* values are provided in Source Data S5. **E** Differential expression of promoter-associated genes between high (greater than 40%) and low drive conversion (less than 15%) groups. Plot structure and analysis are the same as in part D. Exact *p* values are provided in Source Data S6.

We systematically evaluated the drive performance of various Cas9 promoters within a split-drive system targeting the *yellow* gene on the X chromosome. In this system, the gRNA directs Cas9 to cleave *yellow*, enabling HDR to convert the wild-type allele into a drive allele (Fig. 2A). We assessed the drive performance of different Cas9 promoters by performing crosses and analyzing the progeny based on the fluorescence markers and body color phenotypes (Fig. 2B, and Fig. S2).

The constitutive promoters *Act5C* and *Ubi-p63E* display expectedly high somatic expression, consistent with their characterization as ubiquitously active promoters (Fig. 2C, and Fig. S3A). However, both promoters also exhibited unexpectedly high drive conversion of 81% and 73%, respectively. The embryo resistance formation was high at 89% and 91%, respectively. Seventeen other germline promoters also showed high drive conversion rate (over 60%). Among them, the *CG4415* and *SkpE* promoters also exhibited low embryo resistance rate, and *Rcd-1r*, *CG11929*, and *CG15262* exhibited moderate levels of embryo resistance formation. Six promoters exhibited moderate drive conversion rate (ranging from 30% to 60%), with *CG6324*, *CG42854*, *CG13577*, and *CG14357* also having extremely low embryo resistance. Seven promoters showed relatively low drive conversion rate (10% to 30%), among which only *Cyp6a19* displayed high total germline cut rate (it also had high embryo resistance). The remaining six promoters showed moderate to low germline cut rates and did not generate embryo resistance alleles. Additionally, three promoters did not exhibit any detectable cut activity (Fig. 2C, and Fig. S3A). One caveat of this data is that embryo resistance can act on any wild-type allele, including one that avoided germline cleavage. Thus, our germline nonfunctional resistance allele formation rates measured from male progeny and total germline cut rates may be overestimates if some of these formed in the embryo. Given that the Cas9 would have been expressed in the germline in the first place, it is likely that these would only be small overestimates, except perhaps in a fraction of promoters if Cas9 was expressed occurred very late in the germline. Because moderate to high embryo resistance is also associated with mosaic outcomes, we’d expect to see mosaic male progeny (in additional to full yellow phenotype males) if wild-type alleles were uncut in female germline but later affected by embryo activity. Most constructs produced none of these males. They only showed up in appreciable fractions for Cyp6a19 and to a lesser extent fs(1)M3. For these, our germline cleavage rates are likely overestimates. It is possible that these promoter lead to high levels of Cas9 expression in the late germline where there is insufficient time to cleavage all wild-type alleles, resulting in both high maternal deposition and cleavage of maternal wild-type alleles in the embryo.

To understand these results in the context of transcriptome profiles, we performed a systematic correlation analysis between drive performance and the predicted expression patterns of each promoter, as inferred from the Fly Cell Atlas. Among promoters with high total germline cut rate ( > 90%), the five cell types showing the most elevated expression were all female reproductive system cells, four of which were germline (Fig. 2D, and Fig. S4). Several other female germline and non-germline cells showed moderate elevation, and a few cells of other types showed small but statistically significant changes in expression (usually increased expression). Because our total cut rate in this X-linked system was for females, these results are consistent with expectations. We also performed a similar analysis focusing on the drive conversion rate. Compared to promoter genes with low drive conversion (less than 15%), those associated with high drive conversion (more than 40%) showed significantly higher expression across 15 reproductive system cells in *Drosophila*, comprising eight female and seven male cell types, with male germline cells showing the highest levels of effect size and statistical significance (Fig. 2E, and Fig. S5). Because *yellow* is located on the X chromosome and drive conversion rates can only be measured in female individuals, we hypothesize that the statistical significance observed in male germline cells may be due to co-regulation of certain genes in both male and female germline cells, which together with limited signal size and uncertain/less specific female germline cell classification in the transcriptome data set may have resulted in male germline cells yielding the strongest signal in this analysis.

### High drive conversion in different sexes is associated with elevated expression in the corresponding germline cells

**Fig. 3:**
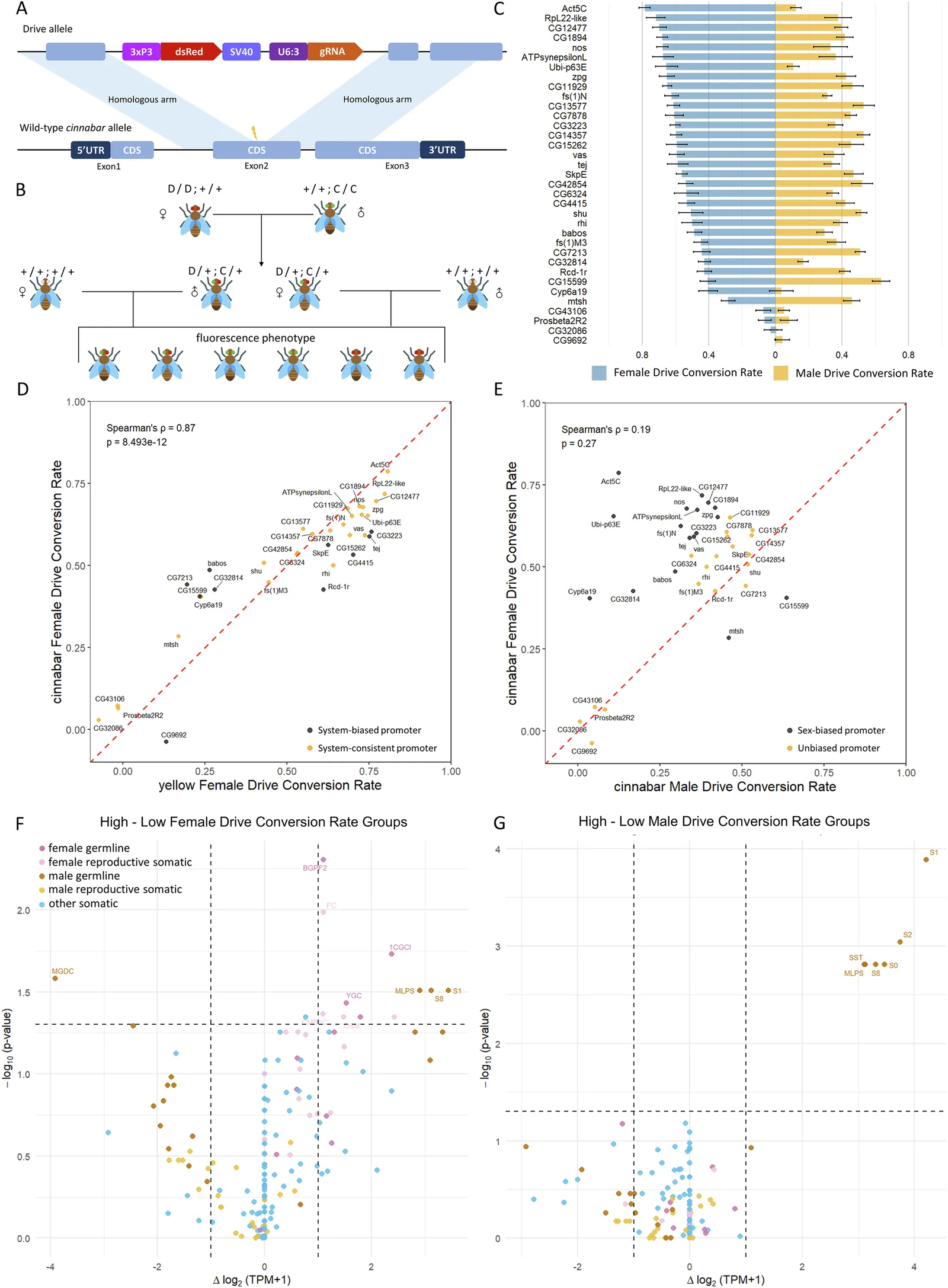
Performance of Cas9 promoters with the split drive targeting *cinnabar.* **A** The gRNA in the split drive element targets *cinnabar*. **B** Females with the drive element are first crossed with Cas9 males. Progeny with both drive and Cas9 were crossed to *w*^*1118*^ individuals. Their offspring were phenotyped. **C** Drive performance of 35 Cas9 constructs regulated by different gene promoters in the *cinnabar* system. For improved visualization, calculated drive conversion rates slightly below zero are displayed as zero. The results for *rhi* were obtained using RO3. Error bars indicate standard error of the mean. Source data are provided in Source Data S3. **D** Comparison of female drive conversion rates between the *cinnabar* and *yellow* drive systems. Orange points indicate promoters showing no significant difference between the systems (*p* ≥ 0.05, based on Welch’s t-test). Spearman correlation analysis revealed a strong positive association between the two systems (ρ = 0.87, *p* = 8.49×10^-12^). **E** Comparison of female and male drive conversion rates in the *cinnabar* system. Spearman correlation analysis revealed a moderate positive association between male and female drive conversion (*ρ* = 0.19, *p* = 0.27). **F** Differential expression of promoter-associated genes between high (greater than 40%) and low (less than 15%) female drive conversion groups. Each point represents a single cell type from the Fly Cell Atlas dataset, with the horizontal axis indicating the difference in log_2_(TPM + 1) between high and low female drive conversion groups, and the vertical axis showing the -log_10_(*p* value) derived from two-sided Wilcoxon rank-sum tests. No adjustment was made for multiple comparisons. The horizontal dashed line indicates the threshold for *p* < 0.05, and the two vertical dashed lines indicate the thresholds for |Δlog2(TPM + 1)| > 1. Top cell types with significant differences (*p* < 0.05 and |Δlog_2_(TPM + 1)| > 1) are labeled with their abbreviated names (Table S2). Exact *p* values are provided in Source Data S7. **G** Differential expression of promoter-associated genes between high (greater than 40%) and low (less than 10%) male drive conversion groups. Plot structure and analysis are the same as in part F. Exact *p* values are provided in Source Data S8.

To compare drive performance between males and females and potentially examine co-regulatory effects of promoters in male and female germline cells, all promoters were tested in another drive system that allows assessment of drive conversion rate in both sexes. This system is also a split gRNA element, but targeting *cinnabar* located on chromosome 2 (Fig. 3A). Drive conversion associated with each Cas9 promoter was assessed through crosses based on fluorescence phenotypes (Fig. 3B, C), but embryo resistance could not be assessed for these because all tested individuals had a *w*^*1118*^ background.

For female drive conversion, the results obtained from the *cinnabar* system were highly consistent with those from the *yellow* system (Fig. 3D), indicating that the influence of promoters on drive performance is likely generalizable across different gene drive systems, although gRNA activity and genetic target could also make important contributions. Moreover, promoters that exhibited high drive conversion in males often showed similarly high conversion in females (Fig. 3E), supporting the existence of a co-regulatory effect between the sexes. We also investigated other promoters with higher female-bias in drive conversion. Compared with promoter-associated genes having unbiased drive conversion, genes with female-biased drive conversion exhibited higher expression in female reproductive system cells (Fig. S6). This could potentially be because male and female germline cells require different expression levels and/or timing to achieve the same drive conversion rate.

Also similar to the *yellow* system, the promoters with high drive conversion rates in females typically displayed significantly elevated expression in female and male germline cells (Fig. 3F), as exemplified by *nanos*, *vasa*, and *fs(1)N*, which both showed high expression in ovary cells corresponding with high female conversion rate (Fig. S7, Fig. S8A–C). In males, the pattern was simpler, with only male germline cells displaying significant differences, predominantly early male pre-meiotic germline cells (Fig. 3G). *CG15599*, *CG12477*, and *CG14357* exemplify this, showing high expression in testis cells and high male conversion rate (Fig. S8D–F, Fig. S9). These findings suggest that high drive conversion in each sex remains closely associated with elevated expression in the corresponding germline cells.

However, we noted exceptions such as *Act5C* and *Ubi-p63E*, which showed high expression in all cell types of both sexes but did not have high drive conversion in males (Fig. 3C), despite high drive conversion in females (Fig. 2C, and Fig. 3C). To investigate this discrepancy, we performed target site sequencing on progeny derived from crosses between drive males and the wild-type females, focusing on offspring that did not inherit the drive. The results showed that all target sites in these progeny were successfully cleaved by Cas9, indicating that the low conversion rates in males were not due to inefficient cleavage, but were more likely a consequence of repair bias away from HDR and toward end-joining (Fig. S10). This observation suggests that high germline expression alone may not always be sufficient for efficient drive conversion, highlighting the importance of possibly considering the HDR efficiency and timing within the germline cells of each sex. It is possible that these ubiquitously active promoters may have expressed Cas9 earlier than our germline promoters, inducing cleavage in germline stem cells earlier. We speculate that cleavage at this time in males (but not females) could be less likely to be repaired by HDR.

### Undesirable drive performance is associated with excessive expression of Cas9

Undesirable drive performance is characterized by limited drive conversion, accumulation of resistance alleles, and fitness cost to carriers. Two key contributors are embryo resistance allele formation and somatic Cas9 expression. Resistance alleles formed in the zygote or early embryo through end-joining can block subsequent cutting and conversion, creating a major barrier to drive spread, especially when the drive itself imposes fitness costs. For suppression drive, this can also lead to premature removal of drive alleles in sterile drive/nonfunctional resistance allele heterozygotes. In addition, somatic Cas9 activity in suppression drives (and some modification drive variants) imposes fitness costs that reduce reproductive competitiveness, ultimately limiting drive power.

**Fig. 4:**
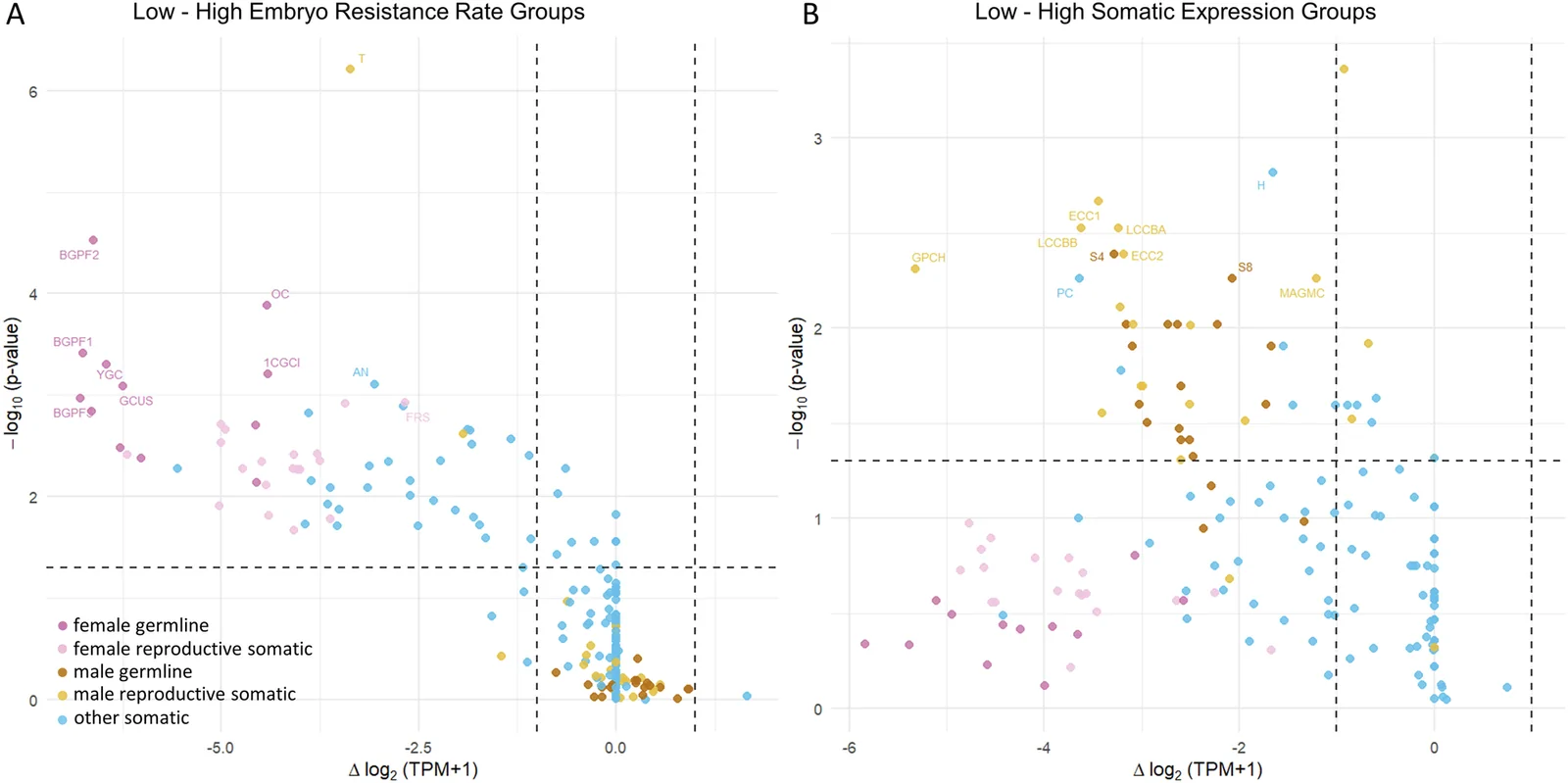
Differential cell type expression analysis of promoter associated genes for low and high embryo resistance and somatic activity in the *yellow* drive system. **A** Differential expression of promoter-associated genes across various cell types between low (less than 5%) and high (greater than 65%) embryo resistance rate groups, restricted to promoters with drive conversion rates above 40%. Each point represents a single cell type from the Fly Cell Atlas dataset, with the horizontal axis indicating the difference in log_2_(TPM + 1) between high and low embryo resistance rate groups, and the vertical axis showing the -log_10_(*p*-value) derived from two-sided Wilcoxon rank-sum tests. No adjustment was made for multiple comparisons. The horizontal dashed line indicates the threshold for *p* < 0.05, and the two vertical dashed lines indicate the thresholds for |Δlog2(TPM + 1)| > 1. Top cell types with significant differences (*p* < 0.05 and |Δlog_2_(TPM + 1)| > 1) are labeled with their abbreviated names (Table S2). Exact *p* values are provided in Source Data S10. **B** Differential expression of promoter-associated genes across various cell types between low (wild-type) and high (yellow) somatic expression groups, restricted to promoters with drive conversion rates above 40%. Plot structure, statistical analysis, significance criteria and multiple comparison treatment are as in part A. Exact *p* values are provided in Source Data S11.

To investigate the relationship between resistance allele formation and Cas9 expression patterns, we compared the transcriptomic profiles of genes corresponding to promoters in the *yellow* drive system that exhibited drive conversion rate higher than 40% (representing potentially viable drive candidates), along with either high embryo resistance rates (more than 65%) or low rates (less than 5%). Based on bulk RNA-seq data, differential expression analysis revealed that the formation of embryo resistance alleles is primarily associated with expression in early embryo stages and female adults, as expected (Fig. S11). Additionally, analysis of single-cell transcriptomic data revealed that the formation of embryo resistance alleles is strongly associated with excessive Cas9 expression in female germline cells (Fig. 4A, and Fig. S12). This is congruent with the notion that embryo resistance allele formation is the result of Cas9 deposition in gametes, which is then transmitted to the zygote. Male-specific cells had no substantial correlation with embryo resistance except for unknown testis cells, which may be related in their expression pattern to female cells, or perhaps include some precursor cell types shared by both sexes.

A similar analysis of promoters associated with high and low somatic Cas9 expression showed that somatic expression from our promoters is not necessarily strongly linked to excessive Cas9 expression in somatic cells, though a few somatic cells types had significant correlation (Fig. 4B, and Fig. S13). Unexpectedly, the majority of significant cell types were associated with the male reproductive system, where high expression was positively correlated with somatic expression. This may be in part due to our selection of promoters that were mostly germline restricted. However, the lack of statistical significance for female cells (despite slightly higher effect size) may well still hold scientific meaning. It may be that when promoters are used with non-native genes such as Cas9 at non-native genomic sites, then male expression patterns are more easily lost, with additional somatic “leakiness” where none existed before. It is also possible that this phenomenon is due to our specific chosen Cas9 genomic locus.

To further assess somatic expression, we used a *doublesex* suppression system^49^ to examine the Cas9 promoter constructs that exhibited low somatic expression in the *yellow* system (Fig. S14). None of the females with both drive and Cas9 displayed a strong intersex phenotype, which was previously indicative of somatic expression^50^. We also found that these females did not exhibit sterility at greater rates than wild-type (though we cannot rule out small to moderate fertility reductions), even with lines that had small amounts of visible somatic activity in the *yellow* system. These results show that several of our Cas9 constructs maintained consistently relatively low somatic expression between systems, though small differences in expression timing and location may somewhat change fitness effects between different targets.

### Ideal drive performance requires a narrow range of Cas9 expression in female germline cells

**Fig. 5:**
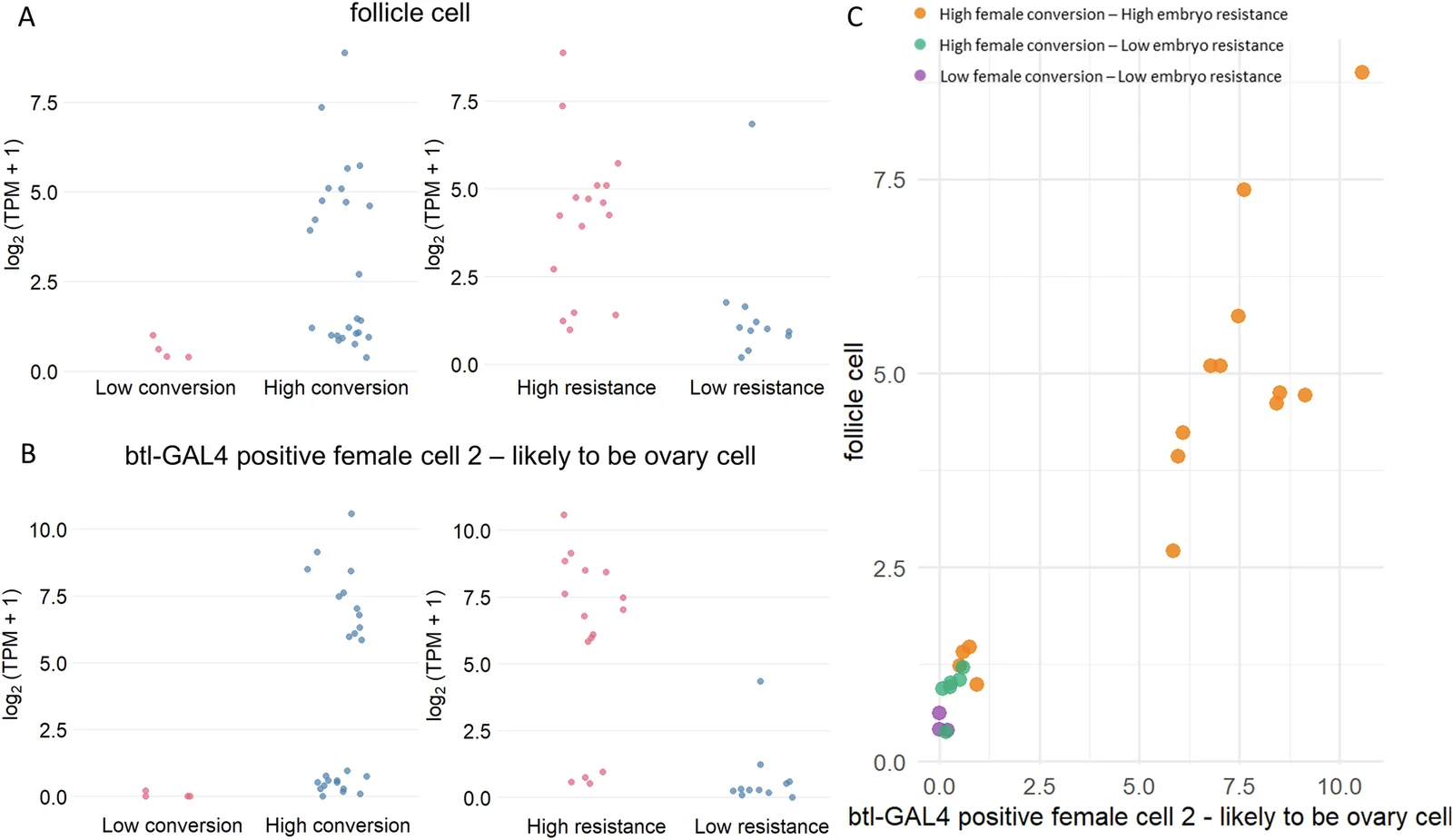
Expression profiles of promoter-associated genes in female germline cells for optimal drive performance. **A** Expression differences of promoter-associated genes in follicle cells. The left panel compares high conversion promoters with low conversion promoters. The right compares high resistance with low resistance. The vertical axis shows log₂(TPM + 1) expression of the genes in follicle cells. Each dot represents the expression value of one promoter-associated gene. Statistical differences were evaluated using the two-sided Wilcoxon rank-sum test (*p* < 0.05 considered significant). No adjustment was made for multiple comparisons. Exact *p* values are provided in Source Data S6 and S10. **B** Expression differences of promoter-associated genes in btl-GAL4 positive female cell 2 - likely to be ovary cell. Plot structure, statistical analysis, significance criteria and multiple comparison treatment are as in part A. Exact *p* values are provided in Source Data S6 and S10. **C** The range of Cas9 expression in female germline cells. The plot shows log₂-transformed expression levels of selected promoter-associated genes in two female reproductive cell types: btl-GAL4 positive female cell 2-likely to be ovary cell and follicle cell. Promoters are grouped into three categories based on their drive performance in the *yellow* system. Each dot represents one promoter.

Ideal drive performance is characterized in part by a combination of high drive conversion rate, low embryo resistance allele formation, and low leaky somatic expression. In examining the relationship between ideal drive performance and single-cell transcriptomic expression patterns, we took as an example two female germline cell types with strong associations according to our previous results to drive conversion and embryo resistance. We found that female drive conversion is associated with high Cas9 expression in these cell types, but low embryo resistance allele formation is linked to low Cas9 expression in those same cell types (Fig. 5A, B). This suggests that achieving ideal drive performance may require Cas9 expression to be maintained within a specific expression window, which could include a temporal window and/or a quantitative window.

To further assess this, we compared the expression profiles of genes corresponding to three kinds of promoters with distinct drive performance: high female conversion-high embryo resistance, high female conversion-low embryo resistance (ideal performance), and low female conversion-low embryo resistance, focusing on their expression in these female germline cell types. Results indicate that optimal drive performance tends to be associated with promoter activity falling within a relatively constrained expression range (Fig. 5C). While this does not rule out a wider expression quantity window if expression timing was further optimized, it does at least indicate that achieving good performance is possible, even if difficult.

We then tested whether the pattern inferred from this framework could be extended to promoter performance with a different gRNA element. Based on the results in the *yellow* and *cinnabar* drive systems, we selected six Cas9 promoter constructs with superior drive performance and evaluated their drive inheritance in a drive system targeting *RpL35A*. This drive was previously found to be effective at removing resistance alleles, yielding high drive inheritance and low to moderate embryo resistance allele formation, but it also had lower germline cut rates in previous tests^8^, perhaps only just enough to support high drive conversion. All six of our Cas9 constructs exhibited high drive inheritance in males, whereas female drive inheritance was comparatively low (Fig. S15). This is consistent with our transcriptomic analysis, which shows that a narrow activity window is necessary for good drive inheritance but low embryo resistance allele formation in females. This lower activity gRNA drive element was close to this ideal window with the high expression *nanos* promoter, which itself had too high of an embryo resistance rate in the *yellow* system. However, for our promoters that had ideal activity in the *yellow* system, their activity was too low in the *RpL35A* system for females. It is interesting that they still maintained high activity in males, a contrast to our investigation in *cinnabar* where there were no candidates that had low activity in females but high activity in males.

### In situ expression of Cas9 at the *rhino* locus prevents leaky somatic expression

**Fig. 6:**
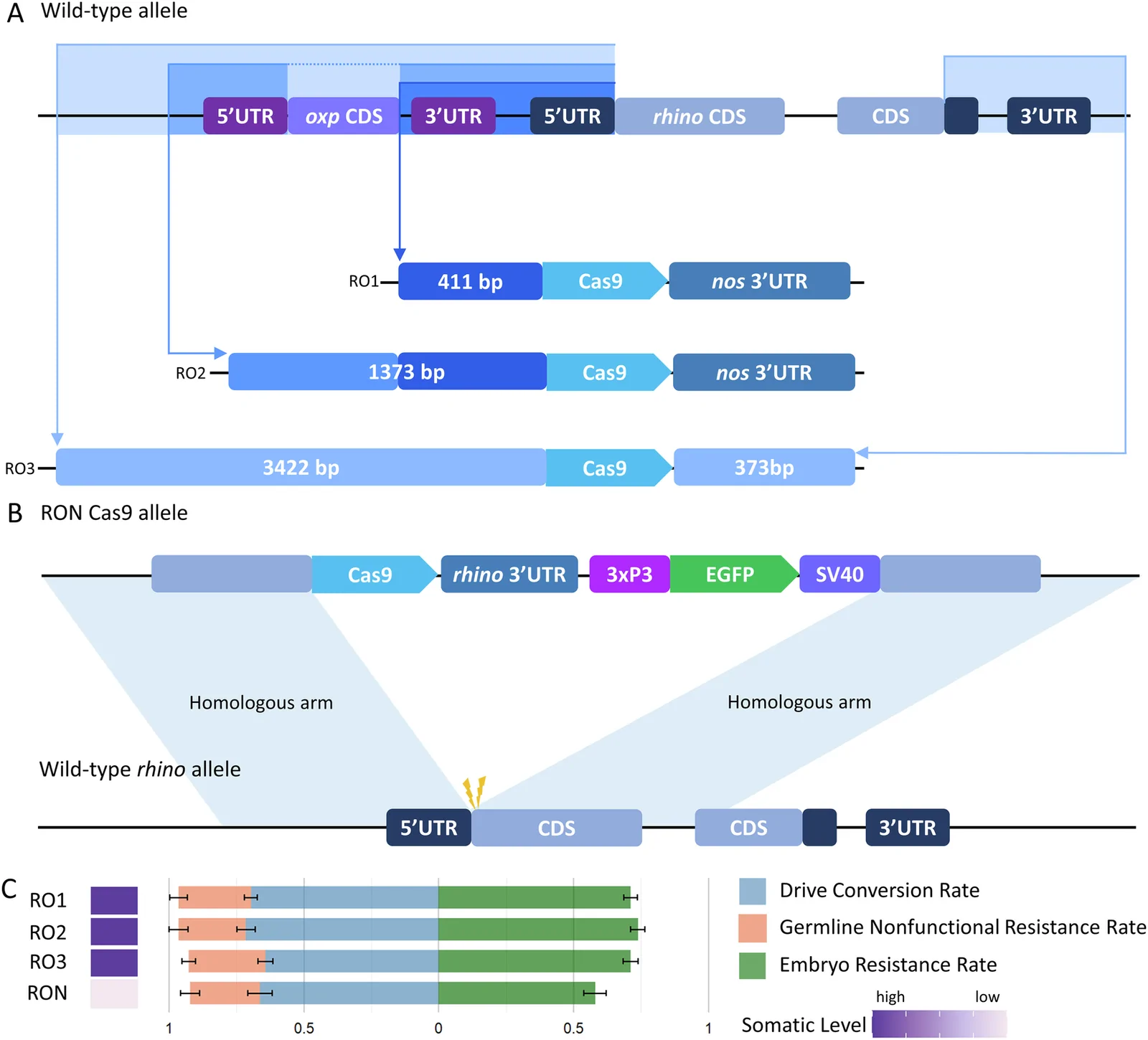
Drive performance of four Cas9 constructs with different *rhino* regulatory regions. **A** Three ectopic Cas9 constructs based on *rhino*. These were integrated into a neutral site on chromosome 2 R and carried EGFP as a fluorescent marker. They contained different lengths of upstream *rhino* regulatory sequence. The downstream regulatory sequence was derived from *nanos* for RO1 and RO2 and from *rhino* for RO3. **B** An in situ Cas9 construct based on the *rhino*. This construct carried EGFP and Cas9, but it was inserted directly into the *rhino* locus, with Cas9 replacing the *rhino* coding sequence immediately after the 5′ UTR. **C** Drive performance of the Cas9 constructs in the *yellow* system. Error bars indicate standard error of the mean. Source data are provided in Source Data S1-S2.

Gene drives usually require use of native gene promoters at non-native sites. In our investigation, we kept the genomic locus constant to facilitate comparison between promoters. Some differences were seen between drive performance and expected native gene expression patterns (Figs. 1–3). This could potentially be due to different genomic locus or due to missing regulatory elements in synthetic promoters. We also used the *nanos* 3ʹ UTR due to superior performance in previous tests^37^, but this could have influenced our promoters in different ways. To investigate how regulatory sequences of different lengths and genomic contexts shape Cas9 expression and drive performance, we selected *rhino* (*rhi*) as a representative germline specific promoter because it showed detectable drive activity, while still exhibiting relatively high embryo resistance and leaky somatic expression, suggesting substantial room for improvement through construct optimization. We designed four Cas9 expression constructs (RO1, RO2, RO3, and RON - only RO3 was used in the analysis in previous sections) derived from the regulatory regions of the *rhi* gene. RO1, RO2, and RO3 were inserted into our usual neutral site on chromosome 2 R and carried 441 bp, 1373 bp, and 3422 bp of the upstream *rhino* regulatory sequence, respectively (Fig. 6A). RO2 excluded the coding sequence of a nearby gene and was thus composed of two separate promoter elements. Transcription termination was mediated by the *nanos* downstream regulatory sequence in RO1 and RO2, and by *rhino* in RO3. The RON construct was generated by inserting Cas9 directly into the endogenous *rhino* locus (together with a copy of the *rhino* 3ʹ UTR), thereby enabling its in situ expression (Fig. 6B). This construct disrupted the native *rhino* gene, causing recessive female sterility^51,52^, so only male carriers instead of male homozygotes were used to generate flies for testing.

Drive performance evaluated through the *yellow* system revealed that the three ectopic Cas9 constructs showed no significant differences in any aspect of drive performance (Fig. 6C). This suggests that promoter element length itself was not a determining factor, as long as the critical regulatory regions are present. In addition, use of the native 3′ UTR did not noticeably alter drive performance. However, the in situ expression construct showed a slight decrease in embryo resistance rate and exhibited markedly reduced Cas9 activity in somatic cells compared to the three ectopic expression constructs. This finding indicates that when Cas9 is expressed in situ at the *rhino* locus, its transcription and translation are subjected to tighter endogenous regulation, thereby reducing leaky expression, especially in somatic cells.

## Discussion

In this study, we evaluated 35 Cas9 lines driven by different promoters across multiple types of gene drive systems. By comparing their performance within these systems, we not only identified promoters with good drive performance but also, through integration with single-cell transcriptomic data, found potential associations between drive performance and promoter properties. Previous studies have suggested that restricting Cas9 expression to germline cells using germline-specific promoters can substantially improve drive performance. Our findings support this notion. Furthermore, our results showed that drive conversion in each sex correlates with the expression level of the promoter-associated gene in the corresponding germline cells. In addition, embryo resistance allele formation is associated with excessive Cas9 expression in female germline cells, while somatic activity is associated with male germline cell expression of native genes. Taken together, these findings reveal that optimal drive performance is strongly associated with a narrow range of Cas9 expression in female germline cells. Though exact quantitative levels may differ across species, this pattern could potentially allow for easier selection of Cas9 promoters in non-model organisms while yielding insights into drive performance complexities.

We evaluated Cas9 promoters across multiple gene drive systems. Initially, we performed a preliminary screen using the *yellow* system^7^. To enable assessment of male drive performance, we then employed the *cinnabar* system. Finally, the promoters with superior drive performance were assessed in well-studied and more advanced drive systems targeting *doublesex*^50^ or *RpL35A*^8^, representing suppression and modification drives, respectively. This strategy demonstrated that Cas9 promoter performance may be at least somewhat generalized across distinct drive systems, even if gRNA activity levels and genomic location will often also play an important role in drive performance.

For the selection of promoter regulatory regions, we identified regions based on transcription factor binding densities from the ReMap database^48^. However, this approach cannot guarantee complete coverage of all regulatory elements, in particular distal regulatory elements (enhancers)^53,54^, features mediated by higher-order chromatin structures^55^, and noncanonical regulatory modes (such as epigenetic mechanisms)^56^. Despite these limitations, integrating our evaluation of drive performance across multiple drive systems with single-cell transcriptomic analyses indicates that, for most cases, the selected regions mechanistically account for differences in drive conversion, resistance allele formation, and leaky somatic expression, and align with the expression levels and temporal patterns of the promoter associated genes in certain cell types. It remains possible that our low-activity promoters in particular may be missing important enhancers. Taken together, though, these observations suggest that the promoter regions included in this study capture much of the critical regulatory elements of their associated genes.

For the selection of 3′ UTR, we uniformly used the *nanos* 3′ UTR rather than the endogenous 3′ UTR corresponding to each promoter. This decision was based on previous findings showing that the *nanos* 3′ UTR not only lacks observable negative effects but may, in some cases, enhance drive performance^37^. Using a consistent 3′ UTR while varying only the promoter allowed us to isolate and evaluate the effects of different promoters. However, gene expression regulation is not solely dependent on the promoter and 5′ UTR^57^. Several studies have shown that 3′ UTRs possess regulatory capabilities comparable to those of promoters^58–60^. Moreover, some endogenous genes have been proven unable to be expressed through promoter activation alone, but instead require simultaneous replacement of the 3’ UTR^61^. These findings suggest that using both the corresponding promoter and 3’ UTR sequences should better approximate expression patterns of endogenous genes. It is possible that some of our promoters did not function or functioned differently due to the use of the *nanos* 3′ UTR. Yet, this study and previous gene drives studies did not show substantially different results when the 3′ UTR was varied, so it remains unclear under what circumstances the exact form of the 3′ UTR becomes important.

Beyond regulation at the promoter and UTR levels, protein stability may also influence Cas9 activity and drive performance. Previous work using PEST sequences to modulate Cas9 protein stability suggests that post-translational regulation can substantially affect effective Cas9 activity^37,62^. Determining protein turnover rates would provide a more complete understanding of how Cas9 expression is regulated after transcription and translation in the future.

In this study, most Cas9 constructs were inserted into a neutral site on chromosome 2 R, downstream of both adjacent genes. Notably, our results showed that our *rhino* promoter in situ construct, while maintaining drive performance comparable to the ectopic constructs, markedly reduced somatic Cas9 activity. This finding further underlines the importance of endogenous spatiotemporal regulation. The in situ construct, by relying on the gene’s own endogenous regulatory framework, achieved more precise control of Cas9 across developmental stages and tissue specificity, thereby effectively avoiding leaky expression in non-target cells. This observation suggests that in situ expression could not only serve as a strategy to reduce the fitness costs associated with somatic expression but also provide a potential means to further enhance the drive performance. Self-limiting split drive strategies and particularly the integral drive strategy^63,64^ are well-placed to take advantage of this finding, though there may be increased complexity in overcoming challenges with resistance alleles and native gene expression as a tradeoff for this. Although integral gene drive designs can exploit endogenous loci and native regulatory elements^65^, most gene drives require the Cas9 gene to be at a non-native locus or cannot take advantage of this strategy for other reasons (for example, suppression drive targets using native regulatory elements to express Cas9 may be cleaved and lose essential expression in heterozygotes unless the delay in CRISPR cleavage is sufficiently long for enough native gene product to first be expressed). It remains unclear whether the native locus in our test had specific regulatory elements or genomic structure that may interact with Cas9 expression or if our chosen site for most of our testing itself makes genes more prone to somatic activity. If it is the latter, then prospects for achieving optimal drive activity based on single cell transcription data are improved because there will usually be multiple possible genomic sites and target genes for drive designs.

During the selection of candidate genes, we attempted to choose promoters with sex-specific expression patterns based on transcriptomic profiles (Fig. 1, Fig. S1). For example, *fs(1)M3* and *babos* were found to be highly expressed in female germline cells, whereas *CG14357* and *CG7213* showed high expression in male germline cells. However, drive performance assays revealed that these promoters exhibited relatively high conversion in both male and female germline cells (Fig. 2C). Transcriptome analysis further revealed that these genes were not entirely silenced in the germline cells of the opposite sex, but it remains surprising that their expression was sufficient for strong drive activity, in line with Fig. 5C showing that promoter candidates with superior drive performance exhibit only low expression in two significant female reproductive cell types.

It is noteworthy that although *Act5C* and *Ubi-p63E* are widely regarded as strong constitutive promoters with similar expression in most cell types, they exhibited marked sex-specific differences in the *cinnabar* drive system. We thus hypothesize that the timing of HDR-biased repair or perhaps Cas9 cleavage with the same promoters differs between male and female germline cells, affecting the outcome of drive conversion. In females, Cas9-mediated cleavage occurring during early germline development is perhaps more likely to engage the HDR pathway, thereby enabling successful drive conversion. In contrast, similar cleavage events in males may be prone to repair through end-joining, resulting in the formation of resistance alleles. This observation implies that the time window for HDR in male germline cells may be later than in females (and note also that expression in early male germline cells appeared to be optimal). Further experimental evaluation may resolve this. Perhaps related to this, the identity of male germline cells in the single-cell transcriptome data has higher confidence that female germline cell types, likely due to much higher quantity and other associated developmental factors. Gaining greater resolution in female cell types and their relative timing may thus allow for improvement in female transcriptomic data, potentially yielding information that could clarify this situation and allow for greater effectiveness when applied for gene drive optimization.

We unexpectedly observed that leaky somatic expression is correlated with high expression of a class of promoter-associated genes in male reproductive system cells. This phenomenon could potentially be attributed to biases introduced during promoter selection. First, in single-cell transcriptomic (scRNA-seq) data, male reproductive system lineages have more granular and consistent developmental stage annotations, whereas the annotation of female lineages is less complete than the former. Consequently, we were more likely to identify and select male promoters with desired expression patterns, resulting in an inflated proportion of male-specific and possible introduction of selection bias. In addition, this limitation influenced our significance analyses. We could pinpoint the optimal stage for male drive conversion with good precision (early pre-meiotic germline cells), whereas for females, we could only could only implicate some early germline cell types and could not confidently assign specific developmental stages to the remaining female germline cells (Fig. 3F, G). Secondly, our selection strategy prioritized promoters exhibiting stage-specificity and high expression within different germline cells. Although this strategy provides a more precise basis for evaluating and comparing drive performance across developmental stages, it also decreases the overall somatic expression in our candidates compared to most other genes. However, the bias described above mainly influences the analysis of somatic expression and is unlikely to exert an impact on the evaluation of other drive performance analyses. The principal reason for this is that these two analyses operate at different levels and are statistically stratified: drive performance such as drive conversion rate and resistance formation rate are evaluated by the relative comparisons in reproductive system lineages, whereas somatic expression is determined primarily by the somatic cells from other tissues. Expansion of promoter candidates to include specificity across multiple somatic organs could provide a more balanced reference for somatic expression analysis and allow a more direct evaluation of whether the correlation observed here reflects a true biological pattern.

We acknowledge that the actual expression pattern of Cas9 may not fully replicate in situ expression of the corresponding genes, but our experimental results nonetheless show that in most cases, they are closely correlated. This indicates that our approach should be broadly applicable despite individual exceptions. Based on the results of this study, we propose a workflow for screening Cas9 promoters across different species to enable efficient Cas9 expression in gene drive systems. First, the required expression activity and spatiotemporal window for Cas9 should be clearly defined according to the mechanism of the designed drive system. For example, in suppression drives, it is necessary to achieve high Cas9 expression in germline cells to enable efficient cleavage at target sites while avoiding somatic expression as much as possible to reduce fitness costs caused by off-target cleavage. Embryo resistance should also be avoided, though this is not as high a priority as avoiding somatic activity. In modification rescue drives, embryo resistance is still harmful, but it is more important to maximum drive conversion. Somatic activity is substantially less harmful than suppression drives. A notable exception to this is drives targeting haplolethal genes, where embryo resistance and somatic expression are harmful to the drive (though with their priorities are probably reversed compared to suppression drives), but potentially still worth the improved rate of nonfunctional resistance allele elimination.

After obtaining high-quality scRNA-seq data or bulk RNA-seq data for the species of interest (which has already been at least partially conducted for some major vectors^44,66^), a screening strategy should be formulated based on the drive requirements. For species with scRNA-seq data, the requirements should first be mapped to the corresponding cell clusters. High drive conversion typically requires medium to high levels of expression in germline cells at early developmental stages, while the low embryo resistance rate favors low or absent expression in germline cells at later developmental stages or in embryos. There is a narrow window where both of these can be achieved. However, it should be noted that this window may be quantitatively different for different species and drives. For example, variance in specific gRNA activity may have allowed a low-expression system to produce good drive conversion in our *yellow* system, but higher activity would be needed for other less active gRNA targets such as our *RpL35A* system to achieve the same total cut rate. To avoid somatic expression, low or absent expression in somatic cells is required, and such absence of somatic activity may be correlated with low expression in male reproductive system cells. The desired expression specificity of each gene in germline cells should then be assessed, prioritizing genes with high specificity as candidates. Then candidate genes with medium to low expression in female germline cells and low or absent expression in late-stage germline cells and early embryos should be further screened. The classification of high, medium, and low expression levels can be referenced against the expression profiles and drive performance of genes corresponding to promoters that have been tested and used in drive systems within the same species to establish relative standards. These are likely to be somewhat different between species, but general patterns should hold. Thus, it may be possible to identify optimal levels with more limited data in non-model species using our *Drosophila melanogaster* data as a reference.

For species with only bulk RNA-seq data available, it is recommended to prioritize scRNA-seq of reproductive tissues (such as ovary or testis) to improve the efficiency and accuracy of subsequent screening. If scRNA-seq data cannot be obtained, a traditional approach can be adopted. By comparing the expression profiles of reproductive tissues with those of embryos or other somatic tissues, candidate genes with high expression and high specificity in reproductive tissues but low expression in new embryos can be screened. However, this runs the risk of yielding candidates with low female germline expression and high female ovary expression, which may be particularly incompatible when used for a suppression drive (where target genes are often but not always expressed in the ovary), which is the drive type that most needs an optimal promoter. Alternatively, homologous genes that have demonstrated good drive performance and are conserved in other species can be referenced. However, the success rate of identifying suitable promoters using this method is mixed^20,37^. It is possible, though, that using both of these methods together may yield more suitable candidates, each method potentially covering drawbacks of the other.

In summary, we interpreted the performance of a large number of different gene drive Cas9 promoters from a single-cell transcriptomic perspective. Our findings provide a theoretical foundation for the efficient selection of promoter elements and the prediction of drive performance. Specifically, we find that early pre-meiotic germline cells are likely the optimal window for male expression and that the quantitative window for optimal drive performance may be narrow. Avoiding somatic expression may be more complicated and could involve a genomic locus. Future work can apply our promoter screening strategy to other organisms, such as disease-vector mosquitoes, in order to optimize drive performance. Additionally, ongoing studies should continue to evaluate the generalizability and applicability of this approach across diverse species and gene drive systems.

## Methods

### Plasmid construction

SNc9CGnG^37^, TTChsp70c9^67^, BHDcN1^8^, and BHDabg1^33^ were constructed previously. SNc9CGnG contains Cas9 driven by the *CG11052* promoter and with the *nanos* 3′ UTR, together with an EGFP marker driven by the 3×P3 promoter. TTChsp70c9 contains Cas9 under the control of the *Hsp70Bb* promoter. BHDcN1 contains Cas9 driven by the *nanos* promoter and with a *nanos* 3′ UTR, a 3×P3-dsRed marker, and a gRNA gene targeting the *cinnabar* gene. BHDabg1 contains a gRNA gene targeting the intergenic region between *gapdh1* and *CG12824* on chromosome 2 R.

SNc9CGnG and BHDcN1 were first digested at unique sites with restriction enzymes (New England Biolabs). Genomic or plasmid DNA of interest were amplified using NEBNext^®^ Ultra™ II Q5^®^ Master Mix. Gibson assembly was performed using NEBuilder^®^ HiFi DNA Assembly Master Mix. The assembled plasmids were transformed into chemically competent *E. coli* strains DH5α (TIANGEN) or XL10 (Angyu). Plasmids were extracted using the ZymoPure Midiprep Kit and verified by Sanger sequencing or full-plasmid sequencing.

### Generation of transgenic lines

Embryo microinjections were performed by UniHuaii and Fungene Biotech. For Cas9 promoter lines, donor plasmids (500 ng/µL) were co-injected with the gRNA helper plasmid BHDabg1^33^ (100 ng/µL) and the Cas9 expression plasmid TTChsp70c9^67^ (450 ng/µL) into *w*^*1118*^ embryos. For the RON line, donor plasmid ISc9ROG (500 ng/µL) were co-injected with the gRNA helper plasmid BHDrhg2 (100 ng/µL) and the Cas9 expression plasmid TTChsp70c9 (450 ng/µL) into *w*^*1118*^ embryos. For the *cinnabar* gRNA line, the donor plasmid (640 ng/µL) was co-injected with TTChsp70c9 (460 ng/µL) into *w*^*1118*^ embryos. Injected individuals were crossed to *w*^*1118*^ flies, and progeny carrying the drive element were identified by fluorescence. After confirming the insertion site by Sanger sequencing of genomic DNA, homozygous lines were established by inbreeding for most lines. However, a homozygous RON line could not be established as the Cas9 insertion disrupts the *rhino* locus, which results in recessive female sterility^51,52^. The gRNA lines targeting *yellow*^7^, *doublesex*^50^, and *RpL35A*^8^ were previously generated. Previously constructed Cas9 promoter lines include: SNc9nG, SNc9SnG, SNc9XnGr, SNc9VnG, SNc9EnG, SNc9CnG, SNc9DnG^37^, and SNc9320nG. Plasmid maps of all new sequences are available on GitHub (https://github.com/jchamper/Single-Cell-Promoters-Homing).

### Fly rearing and phenotypes

Flies were reared on modified Cornell standard cornmeal medium (using 10 g agar instead of 8 g per liter, addition of 5 g soy flour, and without the phosphoric acid) and maintained in vials at 25 °C under a 14/10 h day/night cycle at 60% humidity. Flies were anesthetized using CO₂ and screened for fluorescence using NIGHTSEA adapter SFA-GR for DsRed and SFA-RB-GO for EGFP. Fluorescent protein expression was specifically driven in the white eyes of *w*^*1118*^ under the control of the 3×P3 promoter and the SV40 3’UTR/terminator. EGFP expression indicates the presence of the Cas9 allele, while DsRed indicates the presence of the drive element in individual flies.

In the *yellow* system, loss of *yellow* (*y*) function yields a recessive yellow body phenotype characterized by a lack of dark pigment in adults. We scored body color to infer resistance genotypes and somatic activity. Because *y* is recessive and X-linked, hemizygous males with a disrupted *y* are yellow, whereas females are yellow only when both X-linked copies are disrupted. Resistance alleles were grouped as functional or nonfunctional, of which only the latter contributes to the yellow phenotype. In addition, post-zygotic cleavage and end-joining can occur in embryos, producing flies with partial loss of *y* function. Somatic activity can also result in cleavage of only some cells in the body. In both these situations, individuals were scored as “mosaic”. In practice, flies typically display either wild-type or yellow body and wing pigmentation, but some flies may exhibit a mosaic pattern with mixed yellow and wild-type pigmentation on the dorsal abdomen or wings, which were classified as “mosaic”. In the *cinnabar* system, individuals lacking wild-type alleles usually have a bright orange eye phenotype, but this is not present in *w*^*1118*^ individuals which do not produce eye pigments.

### Drive performance assays

Males from different promoter lines were crossed with females from various gRNA lines to generate male or virgin female progeny carrying both DsRed and EGFP fluorescent markers. Male promoter lines were used in this grandparent cross design to minimize potential maternal Cas9 deposition, which could provide additional persistent Cas9 and affect the drive performance assay. In parallel, somatic expression in the *yellow* system was evaluated by examining the body color of female virgins carrying both fluorescent markers. These individuals were then crossed to the *w*^*1118*^ strain, and their progeny were scored based on fluorescence markers and other relevant phenotypes. In the *doublesex* system, somatic activity was indirectly assessed by the fertility of female virgins carrying both fluorescent markers.

To control for batch effects, statistical analysis was performed in R (version 4.5.0) following established approaches^8,31^. A generalized linear mixed-effects model (GLMM) was fitted using the lme4 package. Each individual was classified into binary categories (e.g., drive vs. non-drive, resistance vs. non-resistance), with vial ID included as a random effect (drive ~ 1 + (1 | group)). After model fitting, marginal means and their standard errors were estimated using the emmeans package to calculate the expected probabilities for each genetic outcome. The program used is available on Github (https://github.com/jchamper/Single-Cell-Promoters-Homing).

### Target site sequencing

To assess cleavage at the *cinnabar* target site, genomic DNA was extracted from individual non-drive progeny (produced by crossing males carrying both the Cas9 element and the *cinnabar* drive element to *w*^*1118*^ females) using DNAzol Reagent (Thermo Fisher Scientific). The extracted DNA was submitted for Sanger sequencing using the primer cinnabar-DNA-S-R, 5′-TAGTTGGCAGGCATCTGGAAG-3′. Sequencing chromatograms were inspected for mixed peaks or heterozygous signals around the target site, which were used as evidence of Cas9 mediated cleavage.

### Cell type-specific differential expression analysis

We obtained the single-cell expression matrix of the Fly Cell Atlas from the Single Cell ExpressionAtlas^47^. Cell type annotations were obtained from the associated metadata file. Gene expression values were then aggregated by cell type by calculating the median expression level for each gene within each cell type. The bulk RNA-Seq data were obtained from FlyBase database (release fb_2024_01)^68,69^. The processed Fly Cell Atlas data and bulk RNA-Seq data used in this study are available in Zenodo (DOI: 10.5281/zenodo.18996871).

Promoter-associated genes were classified based on drive performance as follows. For female germline cut rate (drive conversion + germline resistance allele formation), genes with cut rates greater than 90% (near complete cleavage) and less than 25% (insufficient cleavage to support decent drive conversion in flies, where resistance formation is high) were compared. For female drive conversion, genes with conversion rates greater than 40% (representing at minimum a moderate efficiency drive, and the lowest generally observed when total cleavage rates were high^29,37^) and less than 15% (insufficient to spread quickly or overcome small fitness costs) were compared. For male drive conversion, genes with conversion rates greater than 40% and less than 10% (this was slightly lower than female due to the tendency for reduced male drive efficiency compared to female) were compared. Different thresholds were used for males and females because the distributions of drive performance differed between the two sexes. In particular, female conversion rates were generally higher than male conversion rates, so applying identical cutoffs would have resulted in too few promoters in the female low conversion group and a less balanced comparison. For embryo resistance, genes with drive conversion rates above 40% (representing at minimum a moderate efficiency drive) were further divided based on embryo resistance levels: greater than 65% (representing high embryo resistance) versus less than 5% (negligible embryo resistance). For Cas9 somatic expression, genes with drive conversion rates above 40% were further divided based on body color: wild-type body color (zero/low somatic expression) versus fully yellow body color (high somatic expression). We deliberately contrasted extreme groups (very high and very low cut rate/drive conversion rates) to maximize effect size and improve statistical power. For the embryo resistance and somatic expression analyses, we included only promoters whose drive conversion rate met a predefined threshold, thereby excluding promoters that appeared to have low embryo resistance or somatic expression solely due to insufficient germline Cas9 expression that could not support acceptable drive efficiency and thus did not represent a good candidate for optimal drive performance. For the comparison of female drive conversion rates between the *cinnabar* and *yellow* drive systems and for the comparison of female and male drive conversion rates in the *cinnabar* system, *p-*values were calculated using two-sided Welch’s *t*-tests. For the female-biased analysis, promoters were classified based on the comparison of male and female drive conversion in the *cinnabar* system: promoters with significantly higher female drive conversion were assigned to the female biased group, whereas promoters with no significant sex difference were assigned to the unbiased group. Four promoters lacking observable cleavage activity were excluded because sex bias in drive conversion could not be meaningfully assessed in the absence of detectable cutting.

For each of these pairs of groups, transcripts per million (TPM) differences between them were analyzed across 258 annotated cell types. One cell type was labeled as “unknown” in the original single cell transcriptomic dataset and was therefore excluded from subsequent analyses. To mitigate the effect of zeros, raw TPM was transformed to log_2_(TPM + 1). For each cell type, expression values for the two gene groups were extracted, and differences were assessed for statistical significance using the two-sided Wilcoxon rank-sum test. In parallel, the effect size was defined as the between-group difference in this quantity, denoted Δlog_2_ (TPM + 1). A cell type was considered to show significant differential expression if *p* < 0.05 and |Δlog_2_ (TPM + 1)| > 1.

### Statistics & reproducibility

No statistical method was used to predetermine sample size. No data were excluded from the analyses. The experiments were not randomized. The Investigators were not blinded to allocation during experiments and outcome assessment.

### Reporting summary

Further information on research design is available in the Nature Portfolio Reporting Summary linked to this article.

## Supplementary information

Supplementary Information

Reporting Summary

Transparent Peer Review file

## Source data

Source data

## Data Availability

All raw data is available in the Source Data file, provided with this paper. Target site sequencing data and plasmid sequences are available in (https://github.com/jchamper/Single-Cell-Promoters-Homing). A DOI link is also available (10.5281/zenodo.20595130). The processed single-cell transcriptome data has been made available (10.5281/zenodo.18996871). The original Fly Cell Atlas data^47^ used in this study were downloaded from the EMBL-EBI Expression Atlas under accession code E-MTAB-10519, using the Normalized counts files in MatrixMarket format. Cell type annotations were obtained from the associated metadata file (https://www.ebi.ac.uk/gxa/sc/experiment/E-MTAB-10519/download?fileType=experiment-design&accessKey =). Source data are provided with this paper.

## Code availability

All code is available for free and unlimited use at (https://github.com/jchamper/Single-Cell-Promoters-Homing). A DOI link is (10.5281/zenodo.20595130).
