# Supplementary material for: Finding the perfect promoter for Cas9 in homing gene drives using single cell transcriptome data
Source: Nat Commun. 2026 Jul 25;17:9043. doi: 10.1038/s41467-026-76107-0 (PMC13507122; doi:10.1038/s41467-026-76107-0)
Supplement: Supplementary file 1 [file 41467_2026_76107_MOESM1_ESM.pdf]

Finding the perfect promoter for Cas9 in homing gene drives using single cell transcriptome data

Yingke Wu[1], Yunchen Xia[1], Ziyin Yao[1], Weizhe Chen[1,2], Xihua Jia[1], Nan Liang[1,3*], Jackson Champer[1*]

[1]Center for Bioinformatics, School of Life Sciences, Center for Life Sciences, Peking University, Beijing, China
[2]PTN program, School of Life Sciences, Tsinghua University, Beijing, China
[3]State Key Laboratory of Gene Function and Modulation Research, School of Life Sciences, Biomedical Pioneering Innovative Center and Beijing Advanced Innovation Center for Genomics, Peking University, Beijing, China

*liangn.personal@qq.com, jchamper@pku.edu.cn

Supplemental Information

Supplementary Table 1 List of donor plasmid names with Cas9 regulatory features

**Plasmid name**	**Promoter/5′UTR**	**Length (bp)**	**3′UTR/terminator**	**Length (bp)**
ISc9ROnG	*rhino* (native site)	N/A	*rhino* (native site)	N/A
SNc9ActG	*Act5C*	2482/1837	*Act5C*	1571/121
SNc9banG	*babos*	440/360	*nanos*	839/78
SNc9Cyp6nG	*Cyp6a19*	1825/14	*nanos*	839/78
SNc9fsM3nG	*fs(1)M3*	2020/90	*nanos*	839/78
SNc9fsNnG	*fs(1)N*	2000/61	*nanos*	839/78
SNc9ms1nG	*CG42854*	444/49	*nanos*	839/78
SNc9mtnG	*mtsh*	419/232	*nanos*	839/78
SNc9pp1nG	*CG15262*	96/139	*nanos*	839/78
SNc9pp2nG	*CG15599*	569/68	*nanos*	839/78
SNc9pp3nG	*RpL22-like*	1896/170	*nanos*	839/78
SNc9pp4nG	*CG13577*	848/87	*nanos*	839/78
SNc9pp5nG	*ATPsynεL*	354/253	*nanos*	839/78
SNc9ps2nG	*CG14357*	452*	*nanos*	839/78
SNc9ps3nG	*SkpE*	1915/81	*nanos*	839/78
SNc9RO1nG	*rhino*	371/40	*nanos*	839/78
SNc9RO2nG	*rhino*	1333/40	*nanos*	839/78
SNc9RO3G	*rhino*	3382/40	*rhino*	273/100
SNc9s02nG	*CG12477*	534/145	*nanos*	839/78
SNc9sp0nG	*CG6324*	690/1851	*nanos*	839/78
SNc9sp1nG	*CG1894*	1094/95	*nanos*	839/78
SNc9sp2nG	*CG7213*	1338/124	*nanos*	839/78
SNc9sp3_2nG	*CG9692*	970/44	*nanos*	839/78
SNc9sp3nG	*CG11929*	1570/147	*nanos*	839/78
SNc9sp4nG	*CG43106*	2005/86	*nanos*	839/78
SNc9sp5nG	*Prosβ2R2*	128/92	*nanos*	839/78
SNc9spLnG	*CG32814*	64/1062	*nanos*	839/78
SNc9tejnG	*tej*	897/108	*nanos*	839/78
SNc9UbiG	*Ubi-p63E*	204/1425	*Ubi-p63E*	209/102
SNc9320nG	*CG32068*	1036/31	*nanos*	839/78

Naming convention: SN = split nuclease, c9 = Cas9, G = EGFP
For length of Promoter/5′ UTR, the first number represent the number of nucleotides before the 5′ UTR, and the second represents the number of nucleotides in the 5′ UTR. For length of the 3′ UTR/terminator, the first number represents the length of the 3′ UTR, and the second represents the number of nucleotides included after the 3′ UTR.
*Due to lack of detailed genomic annotation, represents the total 5′ UTR/promoter length.

Supplementary Table 2 Cell Type Abbreviations

Cell Type	Abbreviation
16-cell germline cyst in germarium region 2a and 2b	1CGCI
adult abdominal pericardial cell	AAPC
adult enterocyte	AE
adult tracheocyte	AT
adult neuron	AN
anterior ejaculatory duct	AED
btl-GAL4 positive female cell 1 - likely to be ovary cell	BGPF1
btl-GAL4 positive female cell 2 - likely to be ovary cell	BGPF2
btl-GAL4 positive female cell 3 - likely to be ovary cell	BGPF3
btl-GAL4 positive female cell 4 - likely to be ovary cell sim positive	BGPF4
btl-GAL4 positive female cell 5 - likely to be ovary cell sim+, H15+	BGPF5
central main body follicle cell ca. St. 6-8	CMBFC
early cyst cell 1	ECC1
early cyst cell 2	ECC2
enterocyte of posterior adult midgut epithelium	EOPAM
early elongation stage spermatid	EESS
early-mid elongation-stage spermatid	EMESS
female reproductive system	FRS
follicle cell	FC
follicle cell St. 9+	FCS9
follicle stem cell and prefollicle cell	FSCAP
germ cell stage 4 and later	GCS4A
germinal proliferation center hub	GPCH
germline cell	GC
germline cell, unknown stage	GCUS
gustatory receptor neuron	GRN
hemocyte	H
late cyst cell branch a	LCCBA
late cyst cell branch b	LCCBB
late primary spermatocyte	LPS
main body follicle cell ca. until St. 5	MBFCC
male accessory gland	MAG
male accessory gland main cell	MAGMC
male germline differentiating cell	MGDC
mid-late elongation-stage spermatid	MLESS
mid-late proliferating spermatogonia	MLPS
ovarian sheath muscle	OSM
ovary cell	OC

oviduct	O
pigment cell	PC
polar follicle cell	PFC
post-mitotic endocycling nurse cell	PMENC
post-mitotic germ cell early 16-cell cyst	PMGCE
posterior terminal follicle cell ca. St. 5-8	PTFCC
prefollicle cell/stalk follicle cell	PCSFC
spermatid	S
spermatocyte	SCYTE
spermatocyte 0	S0
spermatocyte 1	S1
spermatocyte 2	S2
spermatocyte 3	S3
spermatocyte 4	S4
spermatocyte 5	S5
spermatocyte 6	S6
spermatocyte 7a	S7
spermatocyte cyst cell branch a	SCCBA
spermatocyte cyst cell branch b	SCCBB
spermatogonium	S8
spermatogonium-spermatocyte transition	SST
stalk follicle cell	SFC
stretch follicle cell	SFC1
testis	T
young germ cell	YGC

Note: only a subset of cell type abbreviations with statistically significance and relatively high rankings are listed in this table.

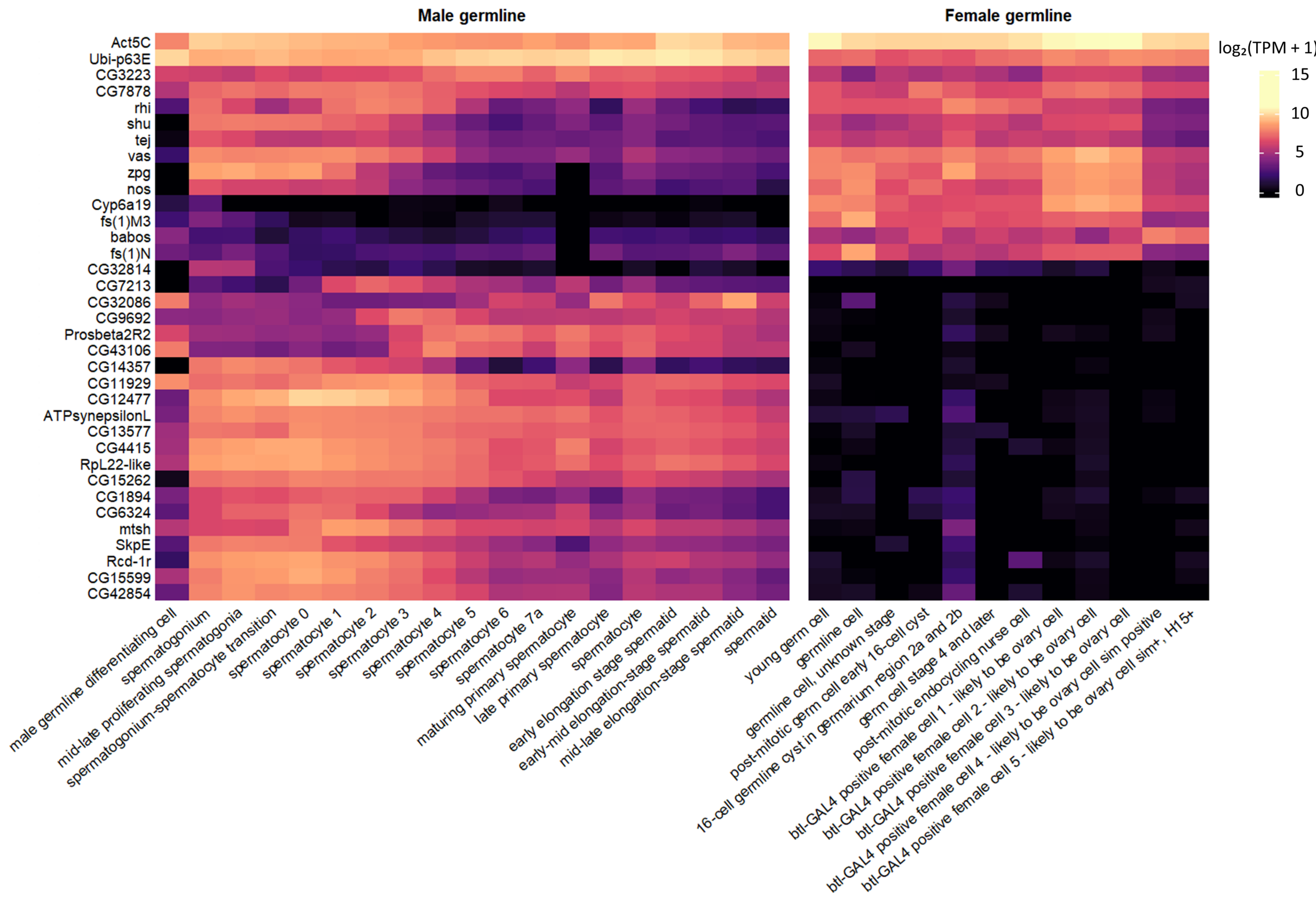

Supplementary Figure 1. Germline cell expression profiles of promoter-candidate genes in *Drosophila melanogaster*. The heatmap displays the expression profiles of 35 gene candidates across 259 single cell subtypes from the Fly Cell Atlas. Raw Transcripts Per Million (TPM) were transformed to $\log_2(\text{TPM} + 1)$ and color-mapped. To determine gene order, Euclidean distances were computed complete-linkage hierarchical clustering. Cell types were reordered to best approximate temporal position in gamete development (earlier cells are to the left).

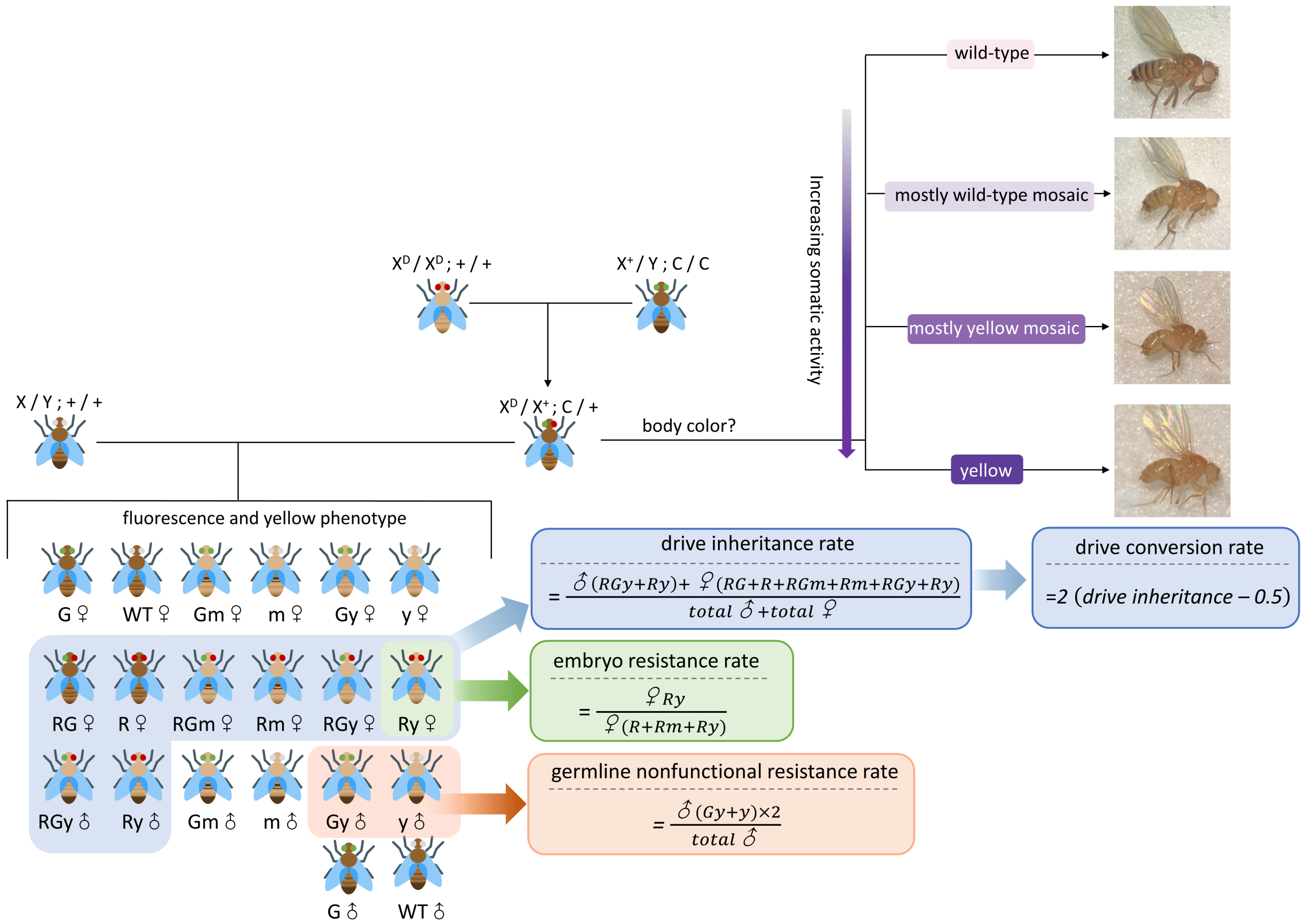

Supplementary Figure 2. Phenotype classification and calculation of gene drive performance parameters. Schematic illustrating how progeny phenotypes were grouped to calculate the drive performance parameters in the *yellow* system. Progeny were classified according to sex, fluorescent marker, and body color phenotype, including dsRed (R), EGFP (G), yellow (y), mosaic for yellow (m), and wild type (WT). X^D refers to the drive allele, and C refers to the Cas9 allele. The drive inheritance rate and conversion rate were calculated based on the proportion of progeny carrying R. Embryo resistance was estimated from the proportion of Ry females among all females carrying R, reflecting resistant allele formation during embryonic development. Germline nonfunctional resistance was estimated from the proportion of yellow bodied male classes (including Gy and y males) among all males, reflecting nonfunctional resistant allele formation in the germline. Somatic expression was evaluated by the body color of female progeny from the initial cross carrying both fluorescent markers. More yellow pigmentation indicated higher somatic expression. Representative images for each class are shown below. Not that in the absence of somatic expression, embryo resistance can be calculated to include EGFP Cas9 females together with the phenotypes shown. Related data are provided in Source Data S2.

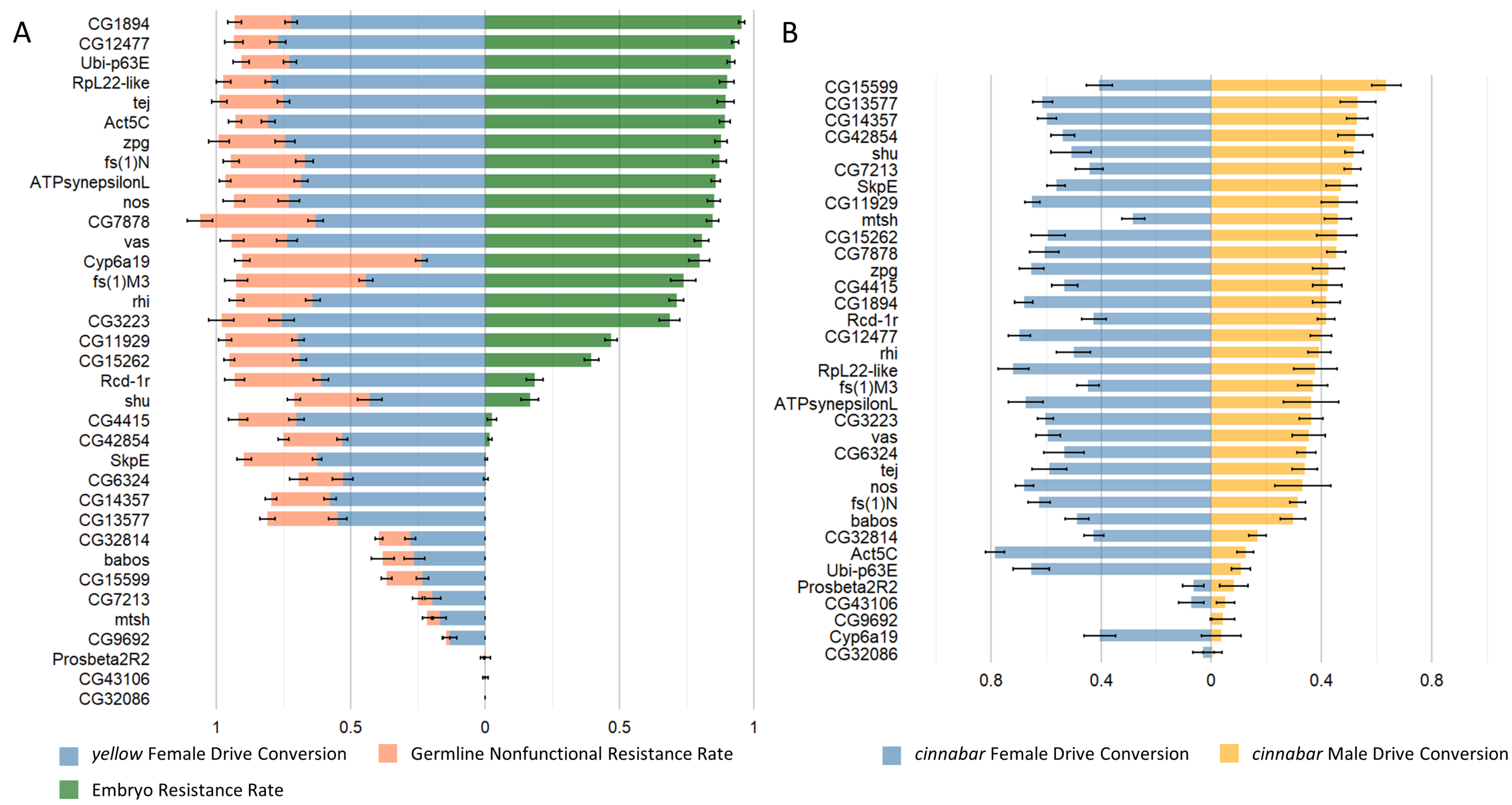

Supplementary Figure 3. Drive performance of Cas9 constructs with different promoters in the *yellow* and *cinnabar* system. A. Drive performance of 35 Cas9 constructs regulated by different gene promoters in the *yellow* system. The sum of the drive conversion rate and the germline nonfunctional resistance rate is nearly equal to the total germline cut rate for each promoter. For visualization purposes, calculated drive conversion rates below zero are displayed as zero. Source data are provided in Source Data S1-S2. **B.** Drive performance of 35 Cas9 constructs regulated by different gene promoters in the *cinnabar* system. Error bars indicate standard error of the mean. Source data are provided in Source Data S3.

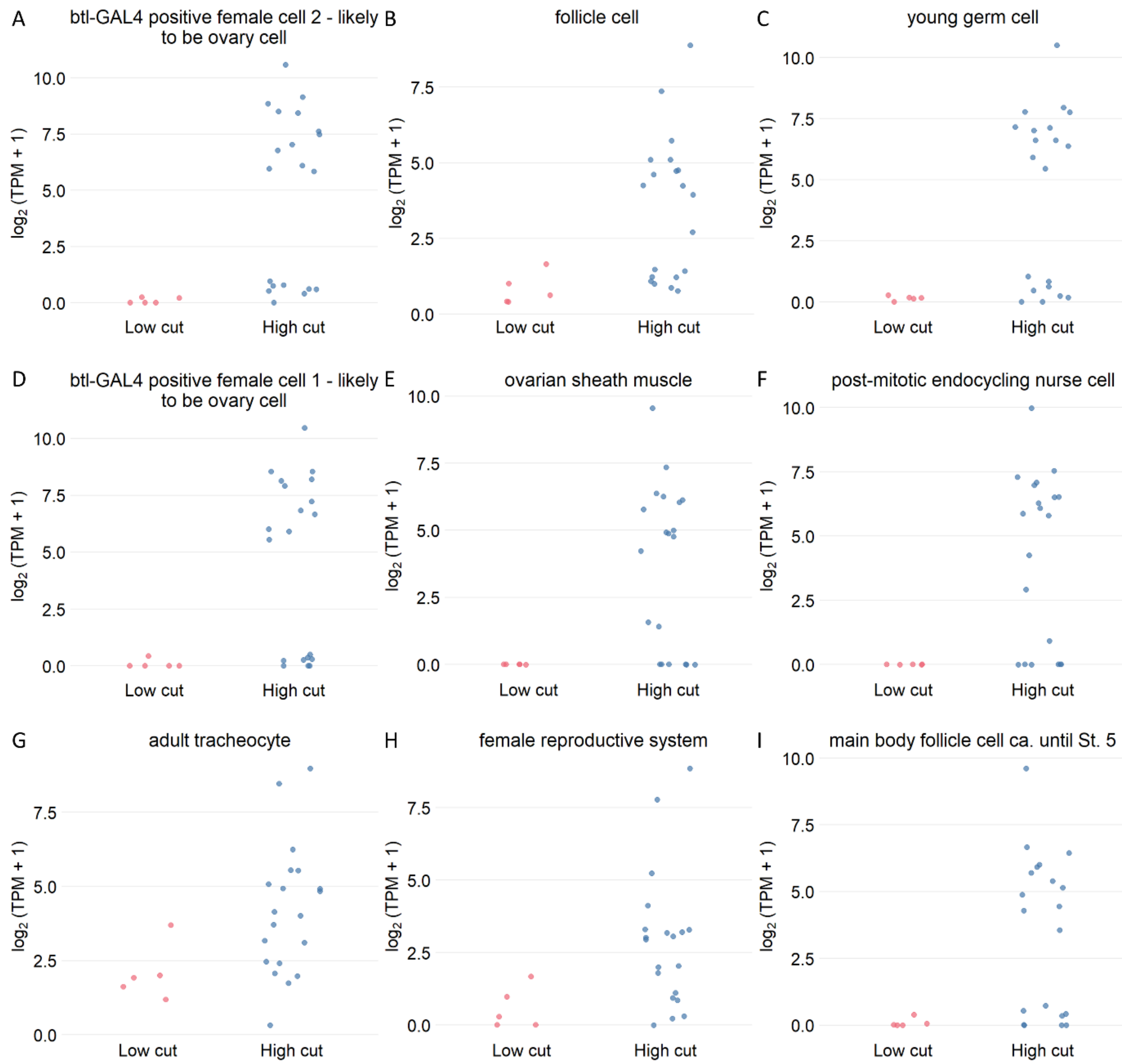

Supplementary Figure 4. Expression differences of promoter-associated genes between high and low total germline cut rate groups in the *yellow* system. Scatter plots showing the $\log_2(\text{TPM} + 1)$ expression of promoter-associated genes in significantly different cell types ($p < 0.05$), comparing high cut rate (greater than 90%) and low cut rate (less than 25%) groups. Panels show the nine most statistically significant cell types: **A.** btl-GAL4-positive female cell 2, likely to be ovary cell, **B.** follicle cell, **C.** young germ cell, **D.** btl-GAL4-positive female cell 1, likely to be ovary cell, **E.** ovarian sheath muscle, **F.** post-mitotic endocycling nurse cell, **G.** adult tracheocyte, **H.** female reproductive system, **I.** main body follicle cell approximately until stage 5. Each point represents the expression of one gene in the cell type. Statistical differences were evaluated using the two-sided Wilcoxon rank-sum test. No adjustment was made for multiple comparisons. The vertical axis shows the $\log_2(\text{TPM} + 1)$ expression of a promoter's gene. Exact p values are provided in Source Data S5.

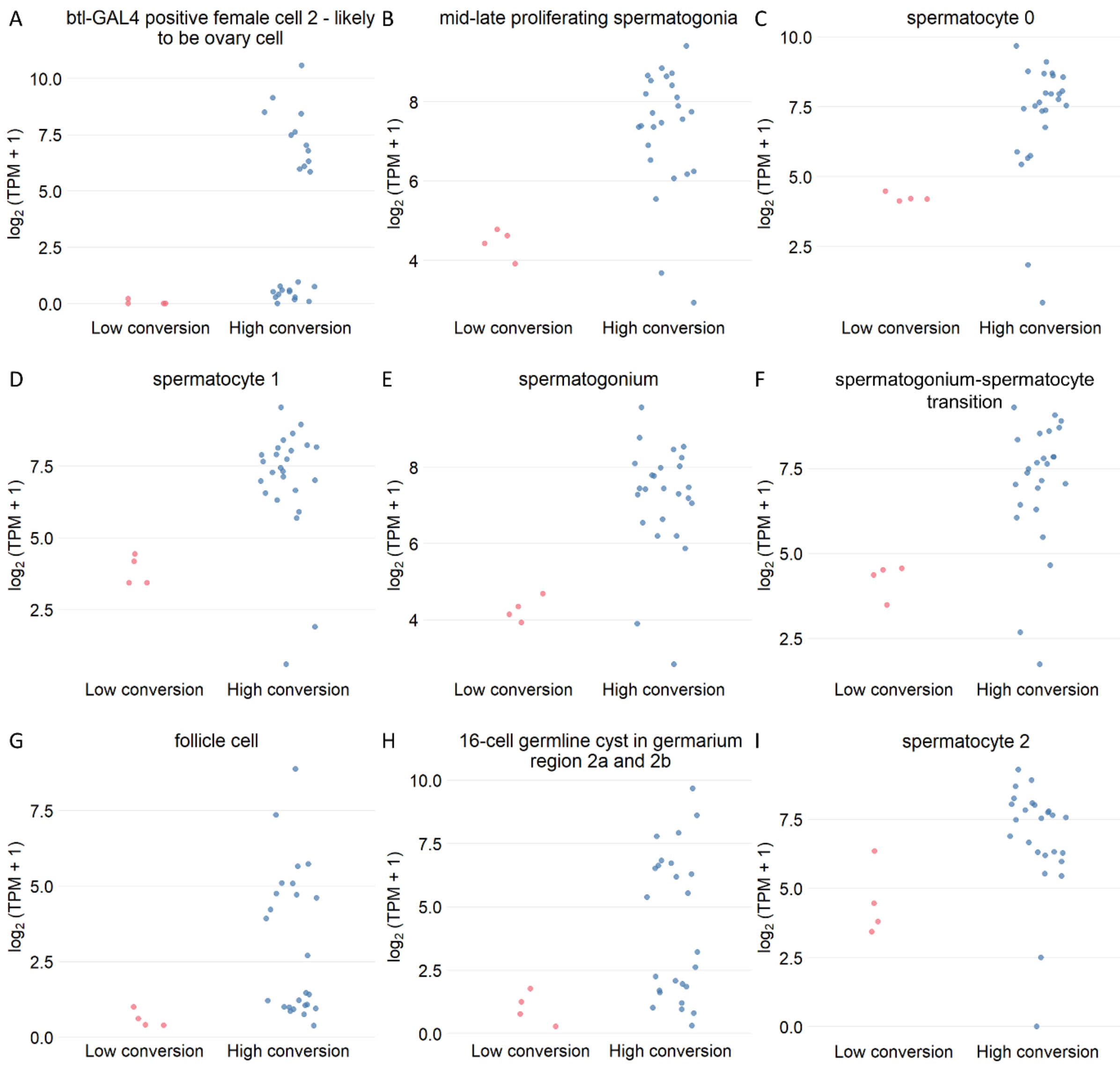

Supplementary Figure 5. Expression differences of promoter-associated genes between high and low drive conversion rate groups in the *yellow* system. Scatter plots showing the $\log_2$(TPM + 1) expression of promoter-associated genes in significantly different cell types ($p < 0.05$), comparing high drive conversion (over 40%) and low drive conversion (under 15%) groups. Panels show the nine most statistically significant cell types: **A.** btl-GAL4 positive female cell 2 - likely to be ovary cell, **B.** mid-late proliferating spermatogonia, **C.** spermatocyte 0, **D.** spermatocyte 1, **E.** spermatogonium, **F.** spermatogonium-spermatocyte transition, **G.** follicle cell, **H.** 16-cell germline cyst in germarium region 2a and 2b, **I.** spermatocyte 2. Each point represents the expression of one gene in the cell type. Statistical differences were evaluated using the two-sided Wilcoxon rank-sum test. No adjustment was made for multiple comparisons. The vertical axis shows the $\log_2$(TPM + 1) expression of a promoter's gene. Exact p values are provided in Source Data S6.

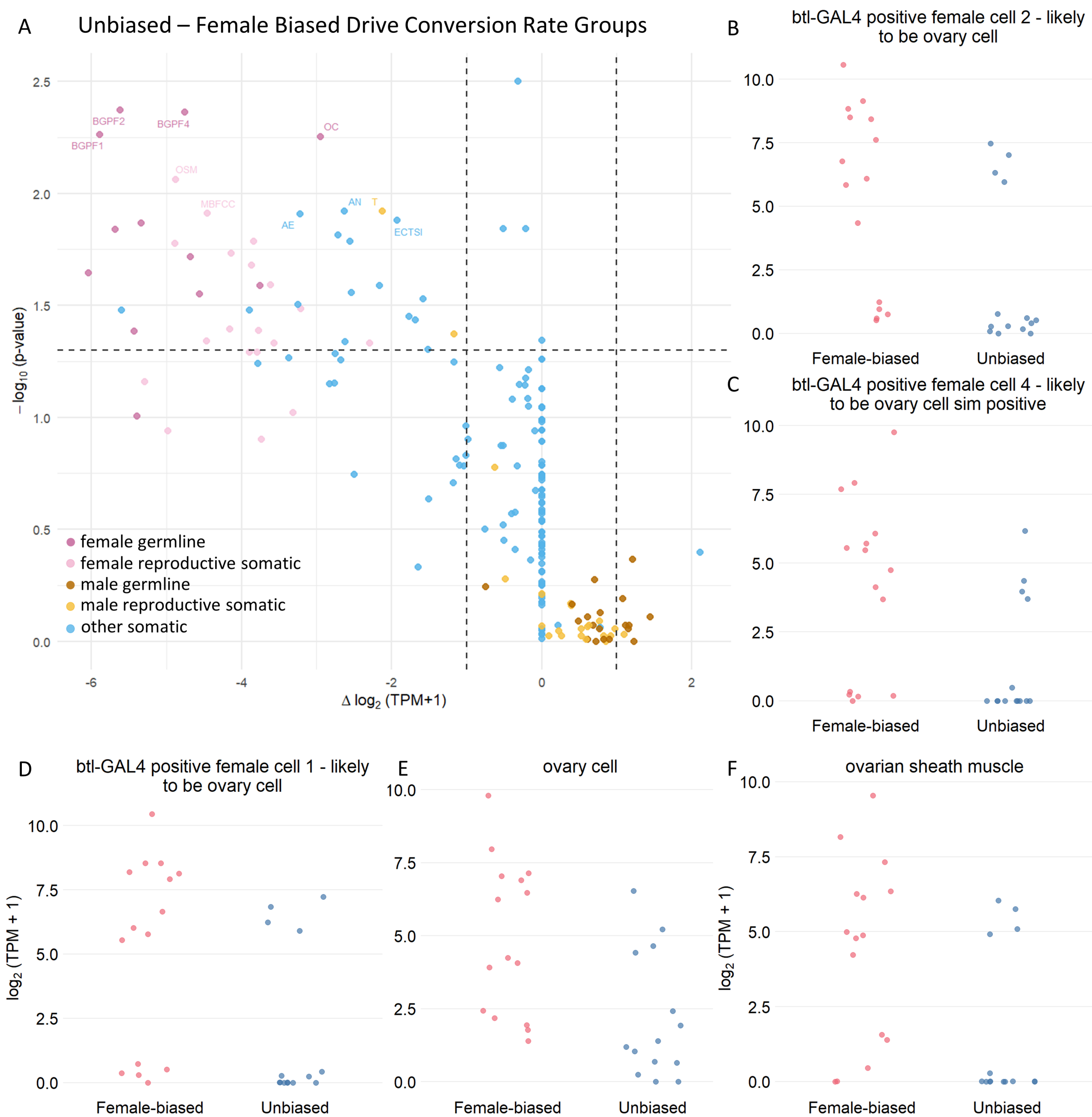

Supplementary Figure 6. Female-biased conversion is associated with distinct promoter-associated gene expression across cell types. A. Differential expression analysis of promoter associated genes between female-biased and unbiased groups across various cell types. Each point represents a single cell type from the Fly Cell Atlas dataset, with the horizontal axis indicating the difference in $\log_2$(TPM+1) between unbiased and female-biased groups, and the vertical axis showing the $-\log_{10}$(p-value) derived from two-sided Wilcoxon rank-sum tests. No adjustment was made for multiple comparisons. Several cell types with significant differences ($p < 0.05$ and $|\Delta\log_2(\text{TPM}+1)| > 1$) are labeled with their abbreviated names (Table S2). Exact p values are provided in Source Data S9. **B-F.** Expression differences of promoter-associated genes between female-biased and unbiased groups. Panels show the five most statistically significant cell types: **B.** btl-GAL4 positive female cell 2 - likely to be ovary cell, **C.** btl-GAL4 positive female cell 4 - likely to be ovary cell sim positive, **D.** btl-GAL4 positive female cell 1 - likely to be ovary cell, **E.** ovary cell, **F.** ovarian sheath muscle. Scatter plots showing the $\log_2$(TPM + 1) expression of promoter-associated genes in significantly different cell types ($p < 0.05$), comparing female-biased

(female drive conversion rate significantly exceeds that of males) and unbiased (female drive and male drive conversion rate do not differ significantly) groups as measured in the *cinnabar* system.

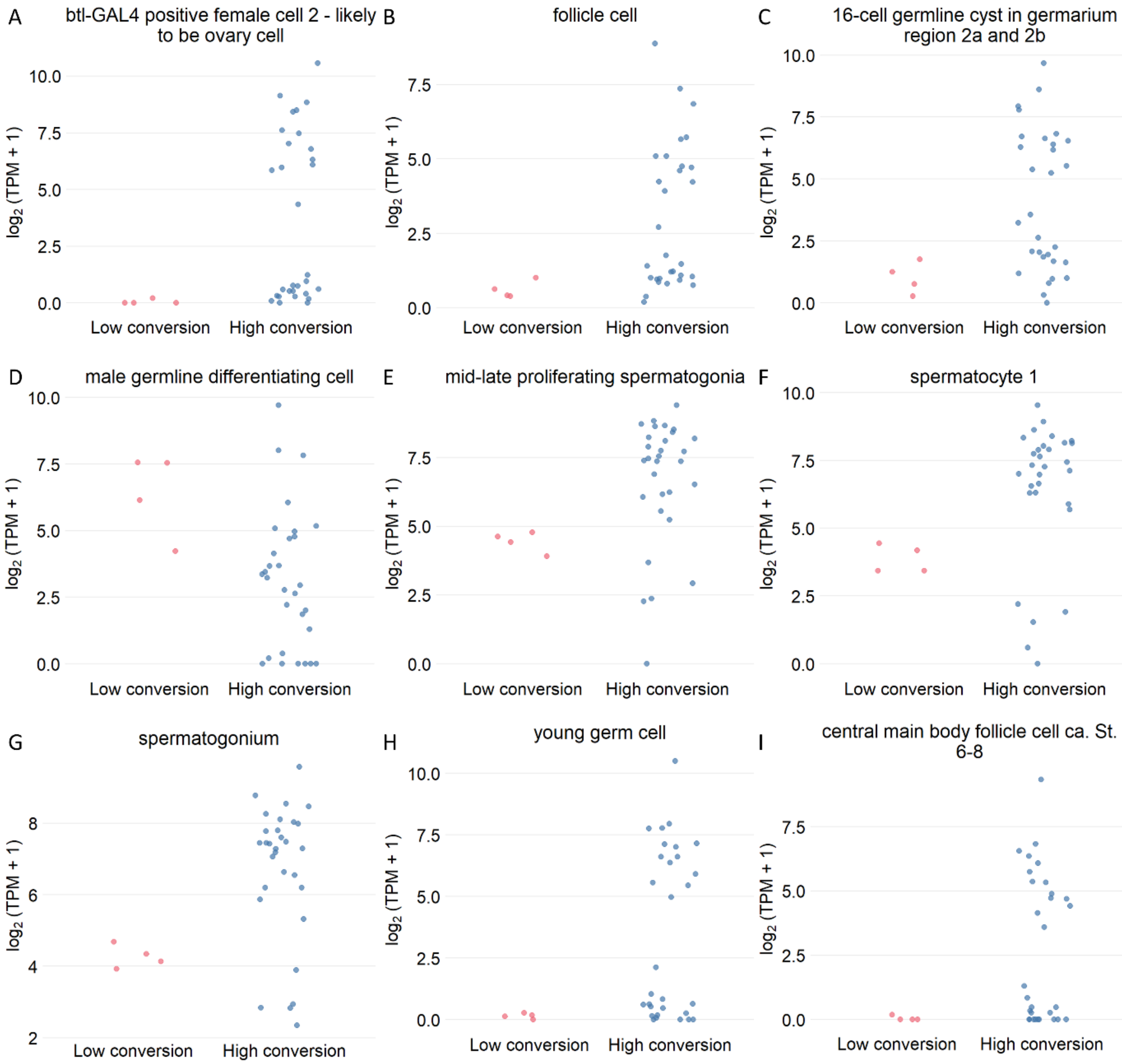

Supplementary Figure 7. Expression differences of promoter-associated genes between high and low female drive conversion rate groups in the *cinnabar* system. Scatter plots showing the $\log_2(\text{TPM} + 1)$ expression of promoter-associated genes in significantly different cell types ($p < 0.05$), comparing high drive conversion (greater than 40%) and low drive conversion (less than 15%) groups. Panels show the nine most statistically significant cell types: **A.** btl-GAL4 positive female cell 2 - likely to be ovary cell, **B.** follicle cell, **C.** 16-cell germline cyst in germarium region 2a and 2b, **D.** male germline differentiating cell, **E.** mid-late proliferating spermatogonia, **F.** spermatocyte 1, **G.** spermatogonium, **H.** young germ cell, **I.** central main body follicle cell ca. St. 6-8. Each point represents the expression of one gene in the cell type. Statistical differences were evaluated using the two-sided Wilcoxon rank-sum test. No adjustment was made for multiple comparisons. The vertical axis shows the $\log_2(\text{TPM} + 1)$ expression of a promoter's gene. Exact p values are provided in Source Data S7.

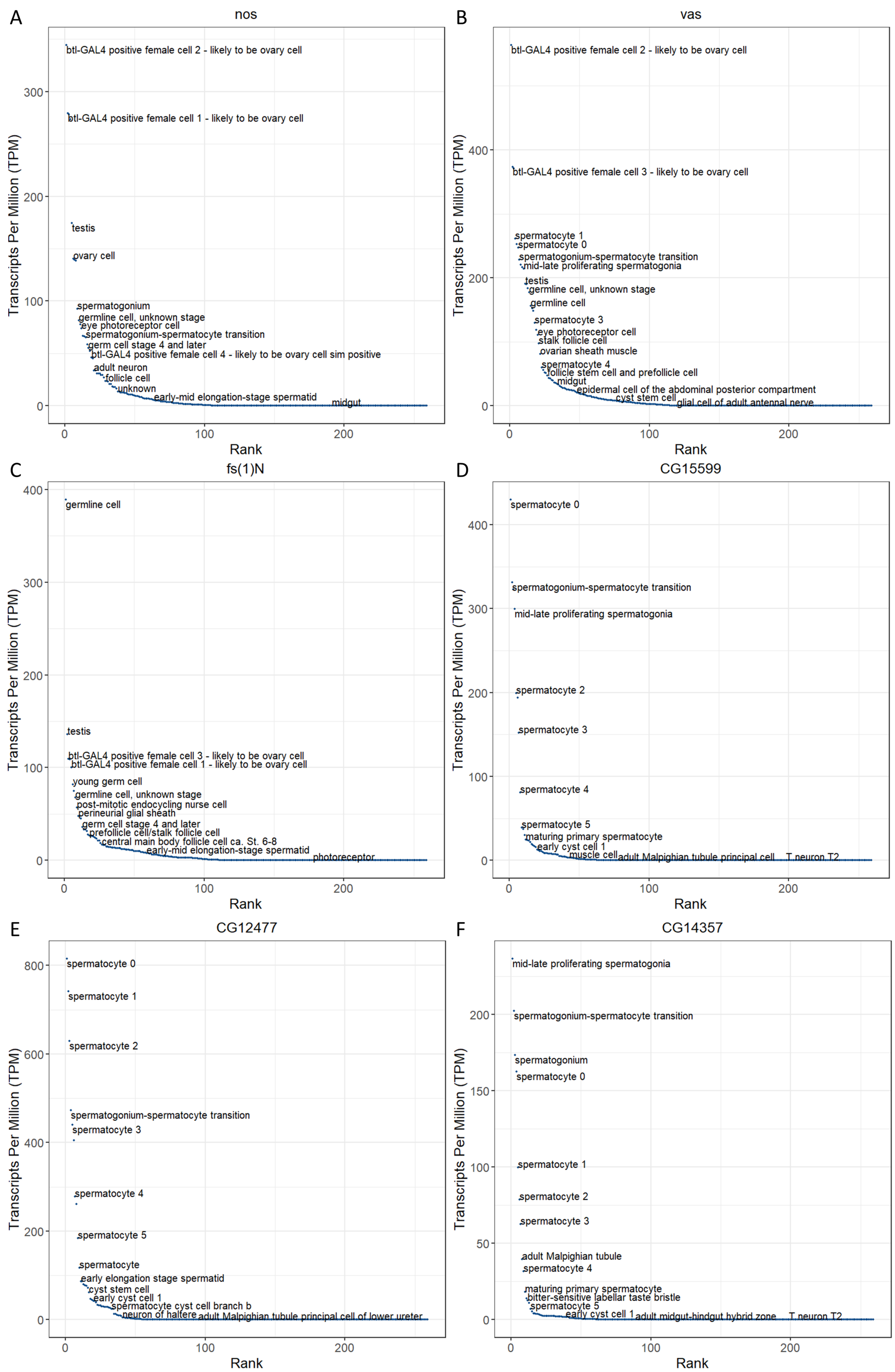

Supplementary Figure 8. Ranked expression distribution of different genes across single cells. Ranked expression profiles of six promoter associated genes across single cells, shown as transcripts per million (TPM). Each point represents the expression level of the indicated gene in one cell type, ranked from highest to lowest expression. **A.** Expression distribution of *nos*, **B.** Expression distribution of *vas*, **C.** Expression distribution of *fs(1)N*, **D.** Expression distribution of *CG15599*, **E.** Expression distribution of *CG12477*, **F.** Expression distribution of *CG14357*.

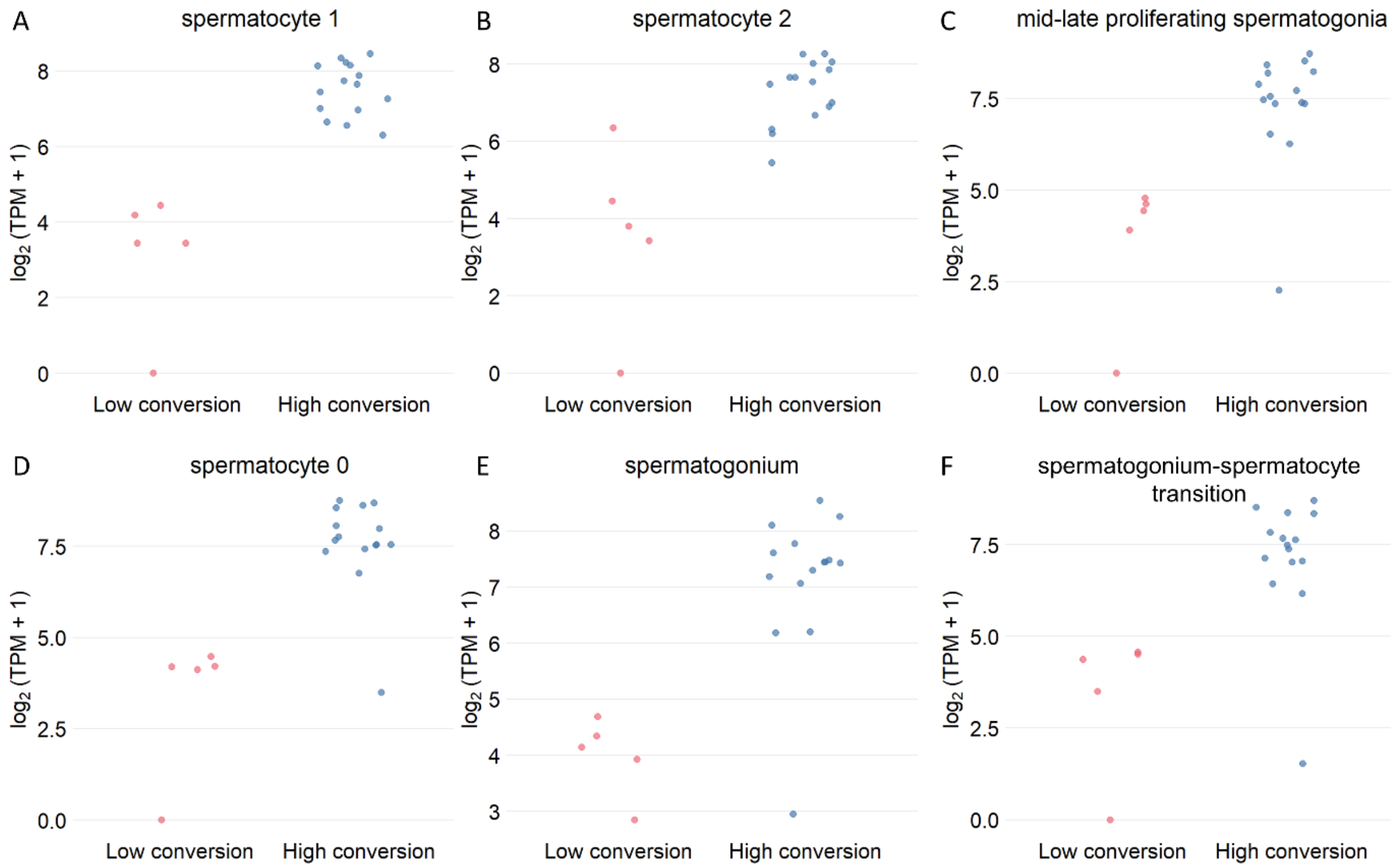

Supplementary Figure 9. Expression differences of promoter-associated genes between high and low male drive conversion rate groups in the *cinnabar* system. Scatter plots showing the $\log_2(\mathrm{TPM} + 1)$ expression of promoter-associated genes in significantly different cell types ($p < 0.05$), comparing high conversion (over 40%) and low conversion (under 10%) groups. Panels show the six most statistically significant cell types: **A.** spermatocyte 1, **B.** spermatocyte 2, **C.** mid-late proliferating spermatogonia, **D.** spermatocyte 0, **E.** spermatogonium, **F.** spermatogonium-spermatocyte transition. Each point represents the expression of one gene in the cell type. Statistical differences were evaluated using the two-sided Wilcoxon rank-sum test. No adjustment was made for multiple comparisons. The vertical axis shows the $\log_2(\mathrm{TPM} + 1)$ expression of a promoter's gene. Exact p values are provided in Source Data S8.

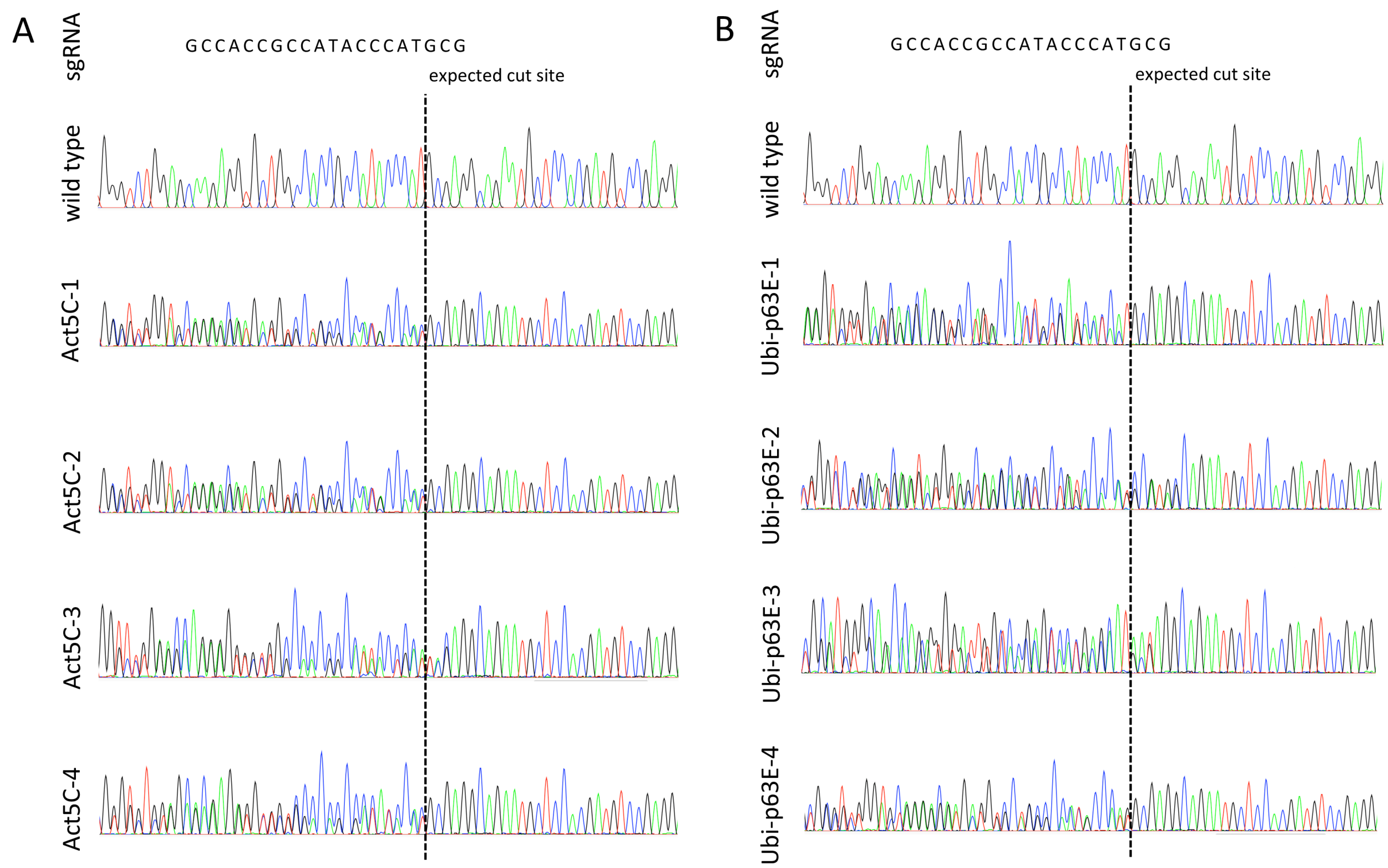

Supplementary Figure 10. Target site sequencing at the *cinnabar* locus. A. Target site sequencing in non-drive progeny from male parents with both the *Act5C* Cas9 element and *cinnabar* drive element. **B.** Target site sequencing in non-drive progeny from male parents with both the *Ubi-p63E* Cas9 element and *cinnabar* drive element. The dashed line indicates the expected Cas9 cut site. Mixed sequencing peaks or heterozygous signals at the target region (present in all four tested of each group) suggest that cleavage events occurred, forming a germline resistance allele in the parent that was inherited by the sequenced offspring.

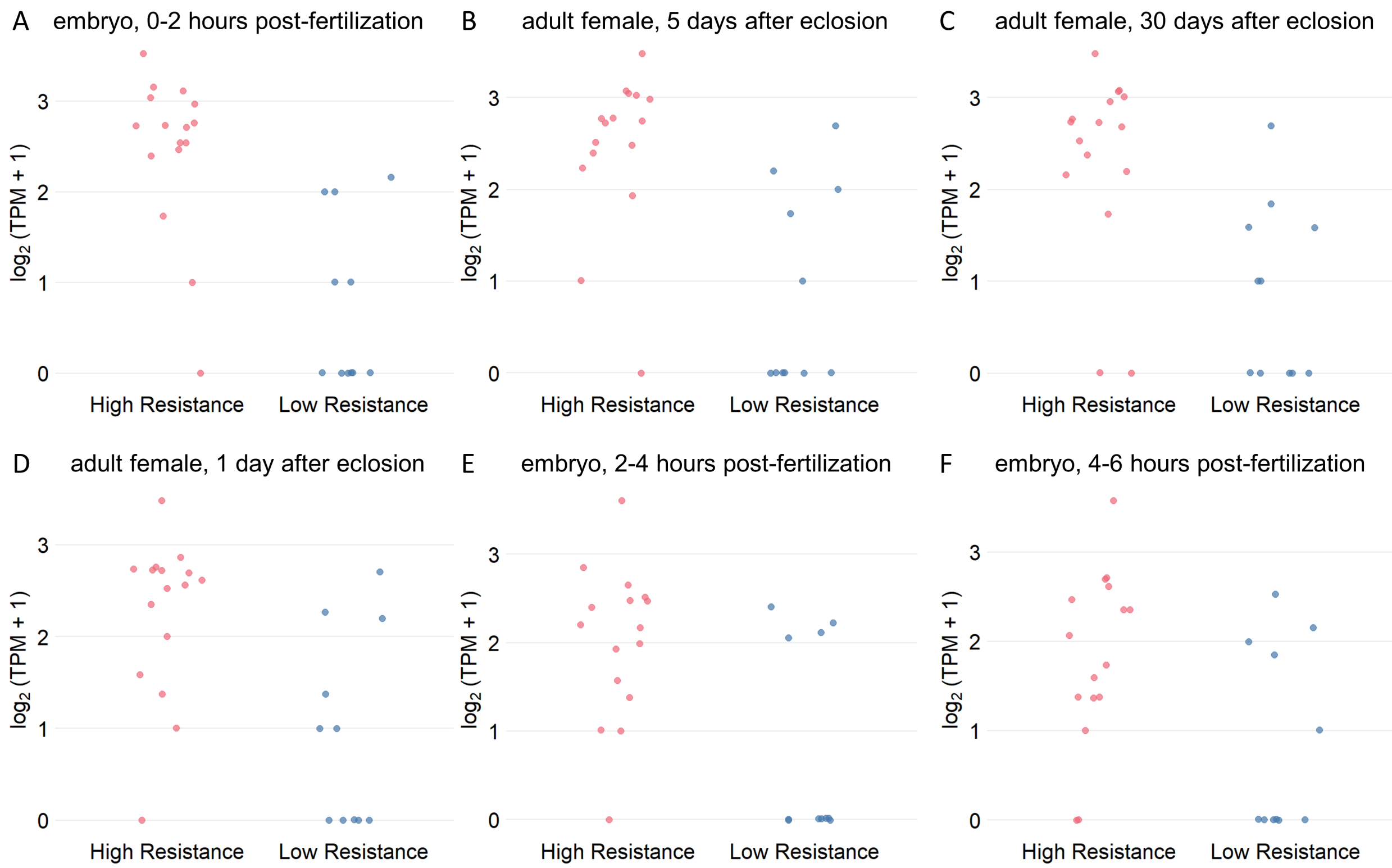

Supplementary Figure 11. Expression differences of promoter-associated genes between high and low embryo resistance rate groups in the *yellow* system, based on bulk RNA-Seq data. Scatter plots showing the $\log_2(\mathrm{TPM}+1)$ expression of promoter-associated genes in significantly different cell types ($p < 0.05$), comparing high (over 65%) and low embryo resistance (under 5%) groups. Only constructs with drive conversion rates greater than 40% were included. Panels show the all six statistically significant stages: **A.** embryo, 0-2 hours post-fertilization, **B.** adult female, 5 days after eclosion, **C.** adult female, 30 days after eclosion, **D.** adult female, 1 day after eclosion, **E.** embryo, 2-4 hours post-fertilization, **F.** embryo, 4-6 hours post-fertilization. Each point represents the expression of one promoter-associated gene in the sample. Statistical differences were evaluated using the two-sided Wilcoxon rank-sum test. No adjustment was made for multiple comparisons. Exact *p* values are provided in Source Data S12.

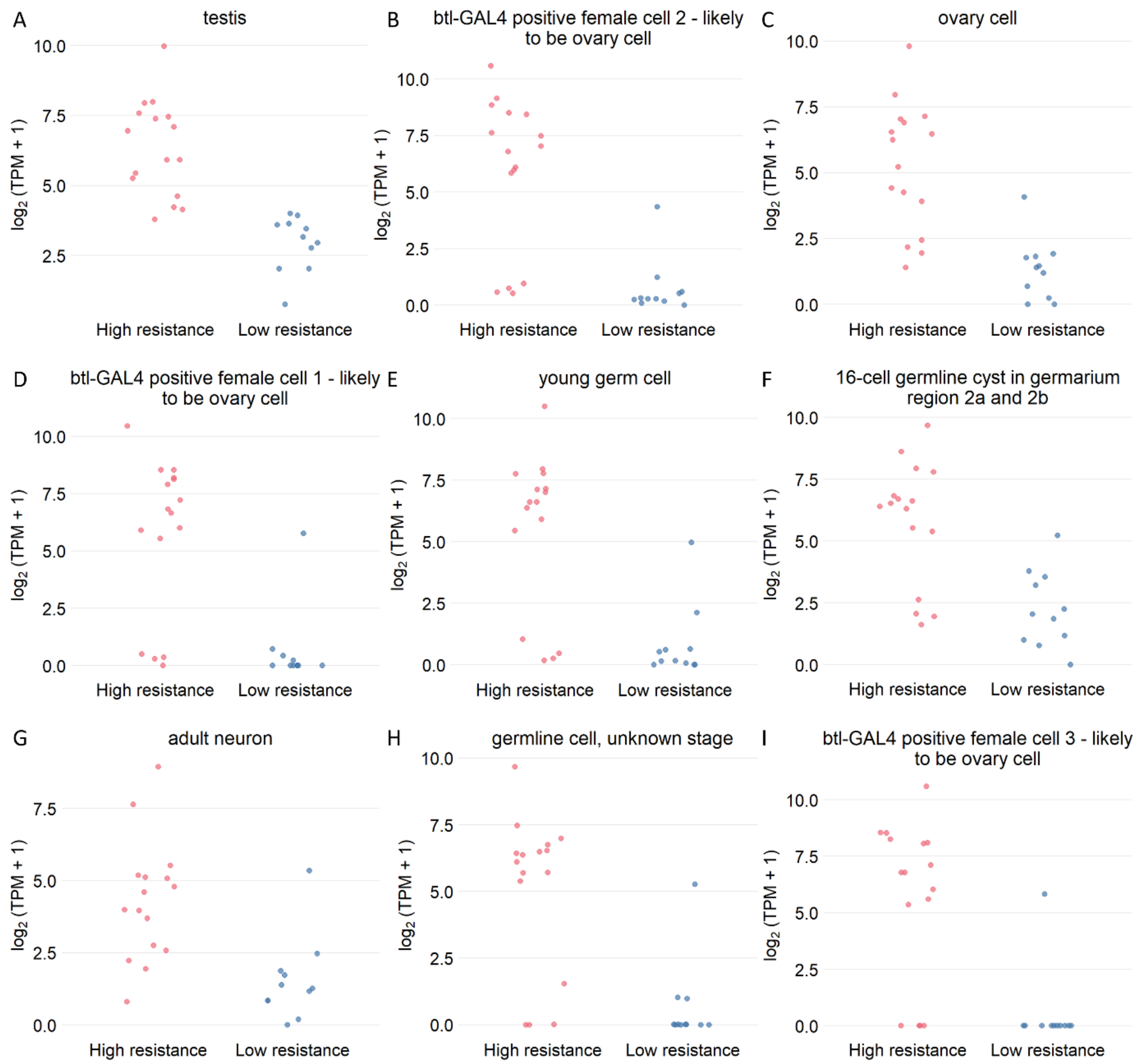

Supplementary Figure 12. Expression differences of promoter-associated genes between high and low embryo resistance rate groups in the *yellow* system, based on single-cell transcriptomic data. Scatter plots showing the $\log_2(\text{TPM} + 1)$ expression of promoter-associated genes in significantly different cell types ($p < 0.05$), comparing high embryo resistance (greater than 65%) and low embryo resistance rate (less than 5%) groups. Only constructs with drive conversion rates greater than 40% were included. Panels show the nine most statistically significant cell types: **A.** testis, **B.** btl-GAL4 positive female cell 2 - likely to be ovary cell, **C.** ovary cell, **D.** btl-GAL4 positive female cell 1 - likely to be ovary cell, **E.** young germ cell, **F.** 16-cell germline cyst in germarium region 2a and 2b, **G.** adult neuron, **H.** germline cell, unknown stage, **I.** btl-GAL4 positive female cell 3 - likely to be ovary cell. Each point represents the expression of one gene in the cell type. Statistical differences were evaluated using the two-sided Wilcoxon rank-sum test. No adjustment was made for multiple comparisons. The vertical axis shows the $\log_2(\text{TPM} + 1)$ expression of a promoter's gene. Exact p values are provided in Source Data S10.

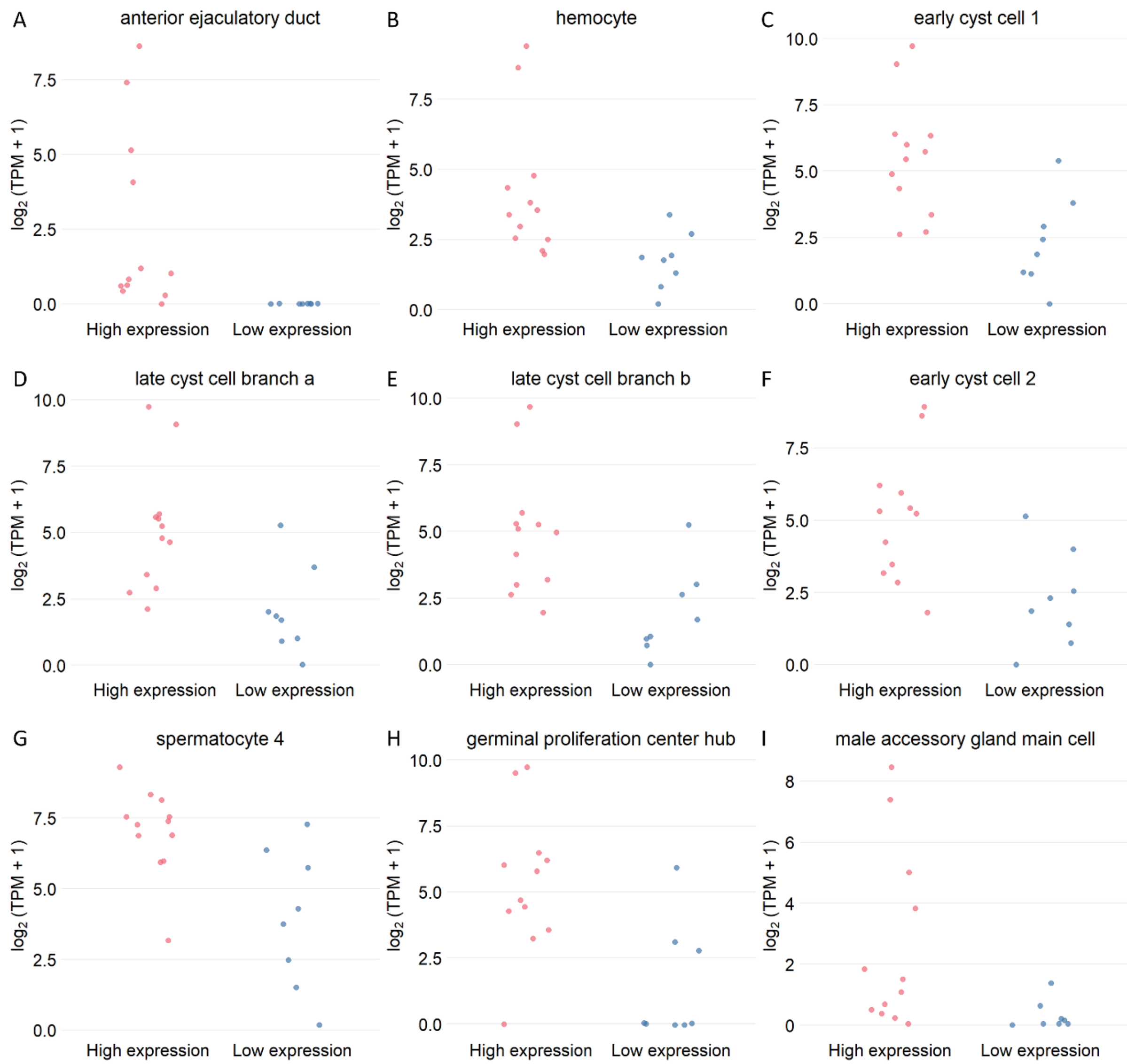

Supplementary Figure 13. Expression differences of promoter-associated genes between high and low somatic expression groups in the *yellow* system. Scatter plots showing the $\log_2(\text{TPM} + 1)$ expression of promoter-associated genes in significantly different cell types ($p < 0.05$), comparing low somatic expression (wild-type) and high somatic expression (yellow phenotype). Only constructs with drive conversion rates greater than 40% were included. Panels show the nine most statistically significant cell types: **A.** anterior ejaculatory duct, **B.** hemocyte, **C.** early cyst cell 1, **D.** late cyst cell branch a, **E.** late cyst cell branch b, **F.** early cyst cell 2, **G.** spermatocyte 4, **H.** germinal proliferation center hub, **I.** male accessory gland main cell. Each point represents the expression of one gene in the cell type. Each point represents the expression of one gene in the cell type. Statistical differences were evaluated using the two-sided Wilcoxon rank-sum test. No adjustment was made for multiple comparisons. The vertical axis shows the $\log_2(\text{TPM} + 1)$ expression of a promoter's gene. Exact p values are provided in Source Data S11.

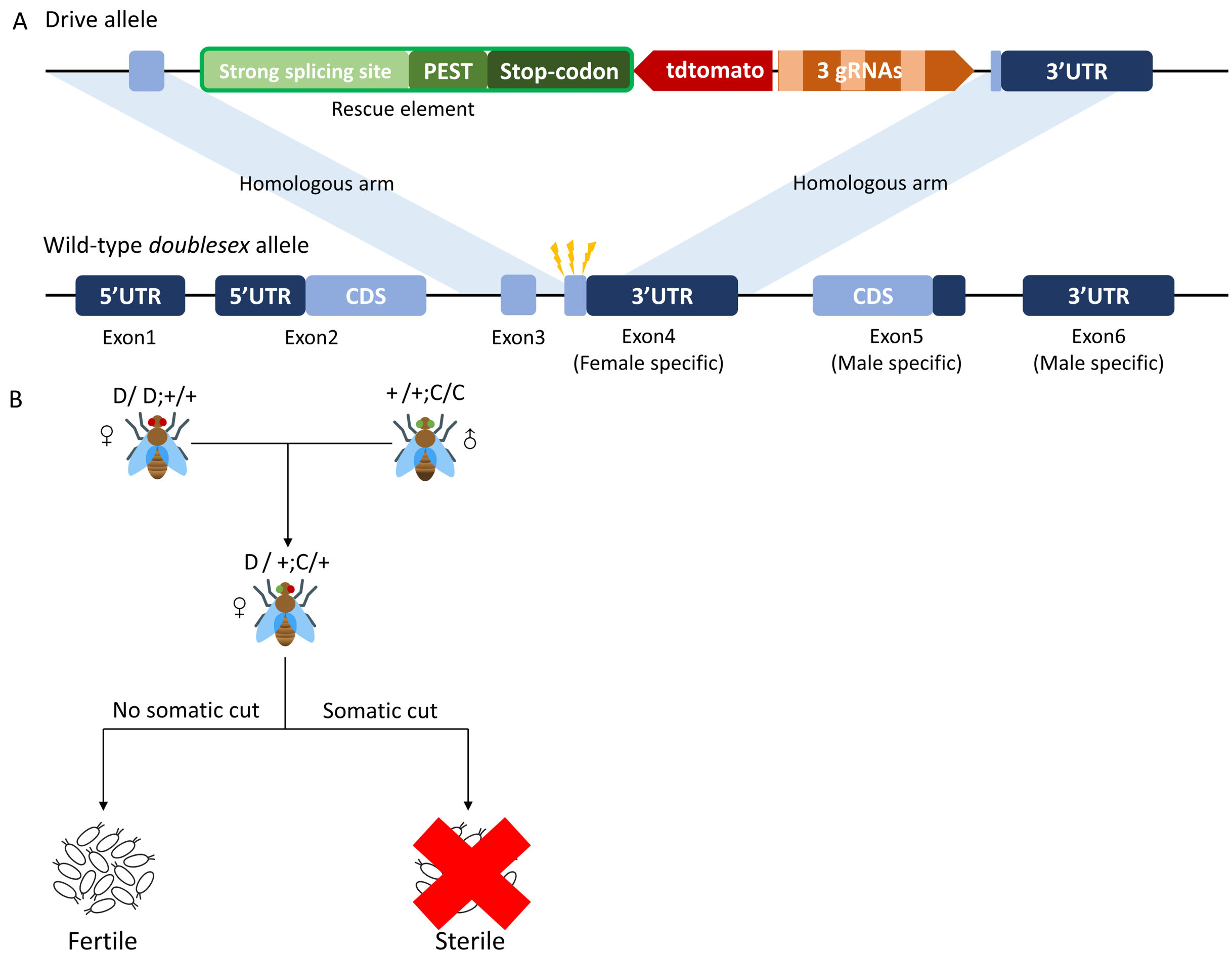

Supplementary Figure 14. Assessment of somatic expression in the *doublesex* systems. A. The *doublesex* system includes two components: a split Cas9 element and the drive element. The split Cas9 element is as previously described. The drive element contains three gRNAs targeting female-specific exon 4 *doublesex*, which are linked by tRNAs and expressed by the U6:3 promoter. Females with disrupted *doublesex* in both chromosomes have an intersex phenotype. **B.** The cross scheme of *doublesex* drive system for evaluating somatic expression. Females with the drive element are first crossed with males with the Cas9 element. Females carrying both elements are evaluated by crossing them with wild-type males and assessing their fertility (none of these females displayed increased intersex characteristics). Related data are provided in Source Data S2.

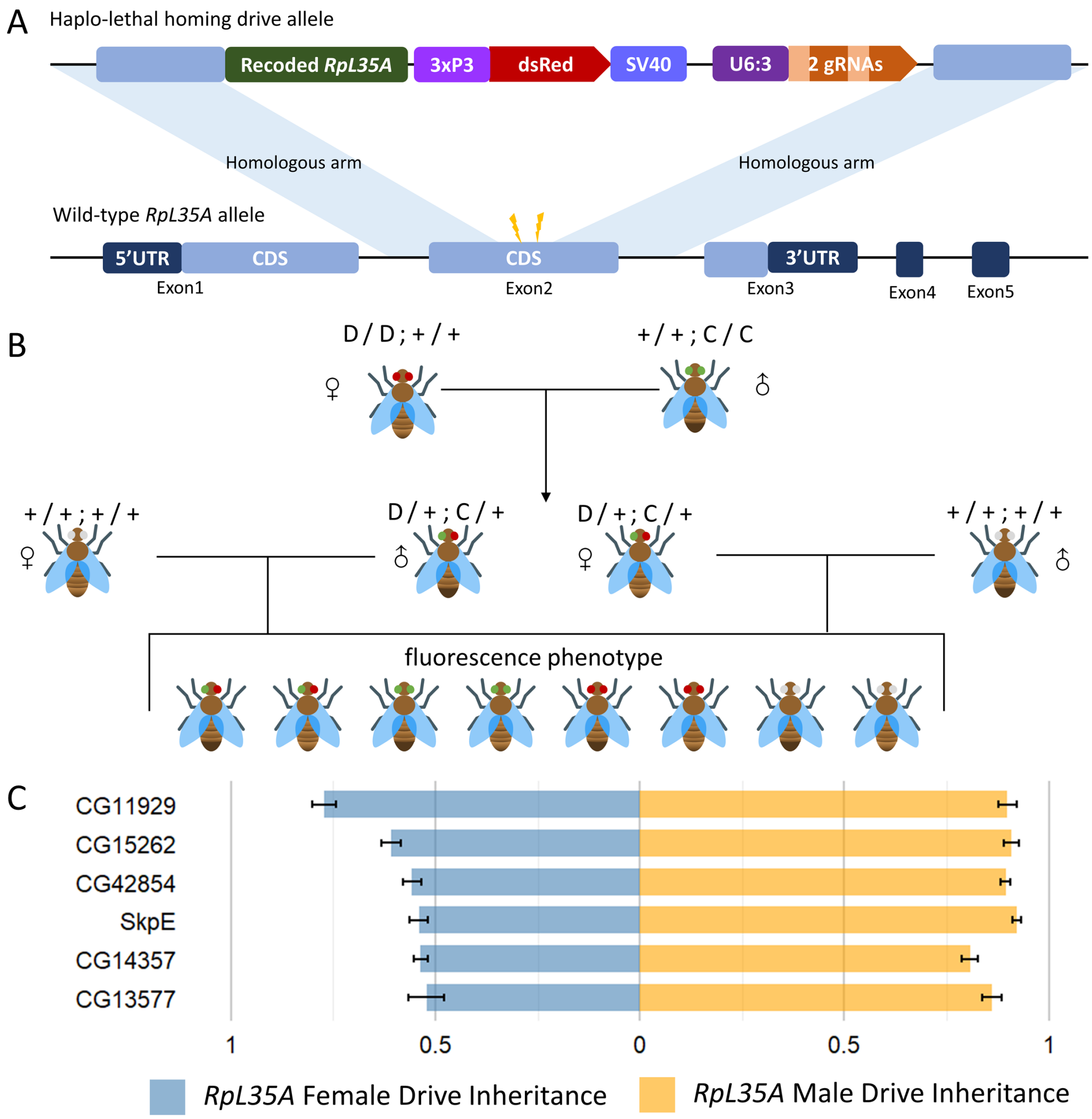

Supplementary Figure 15. The split drive system targeting *RpL35A*. A. The *RpL35A* system includes two components: a split Cas9 element and the drive element. The drive element contains two gRNAs targeting exon 2 of *RpL35A*, which are linked in tandem via a tRNA and expressed by the U6:3 promoter. *RpL35A* is a haplolethal gene encoding a highly conserved protein component of the 60S ribosomal subunit, so nonfunctional resistance alleles result in nonviability, regardless of the other allele. **B.** The cross scheme of the *RpL35A* drive system. Females with the drive element strain are first crossed to males with the Cas9 elements. Female or males carrying both fluorescent markers are selected from the progeny and crossed to w^{1118} flies. Drive performance was assessed by analyzing the fluorescence phenotypes of the resulting offspring. **C.** Drive performance of six Cas9 constructs regulated by different gene promoters in the *RpL35A* system. Error bars indicate standard error of the mean. Source data are provided in Source Data S4.
