# Supplementary material for: Finding the perfect promoter for Cas9 in homing gene drives using single cell transcriptome data
Source: Nat Commun. 2026 Jul 25;17:9043. doi: 10.1038/s41467-026-76107-0 (PMC13507122; doi:10.1038/s41467-026-76107-0)
Supplement: Supplementary file 3 [file 41467_2026_76107_MOESM3_ESM.pdf]

nature portfolio

Peer Review File

Finding the perfect promoter for Cas9 in homing gene drives using single cell transcriptome data

Corresponding Author: Professor Jackson Champer

This file contains all reviewer reports in order by version, followed by all author rebuttals in order by version.

Version 0:

Reviewer comments:

Reviewer #1

(Remarks to the Author)
The manuscript of Wu et al. identifies genes by their expression patterns in publicly available single-cell transcriptomic data sets, takes likely-promoter regions and uses them to express cas9. Thirty-five such constructs are then tested for attributes relevant to 'gene drive' technology: the efficiency at which cut and home in the germline, their cutting rate in the soma, the rate at which different classes of resistance arise. They do this among multiple gene drive designs that their impressive group has previously developed. While their overall conclusion that the timing of cas9 expression matters is unsurprising, the value of this paper is in the details, and how they differ between genetic biocontrol designs.

I found the introduction well structured around three 'aspects' of gene drive performance (conversion rates, resistance alleles, fitness costs) and I appreciated that the Discussion provided guidance to future research into finding promoters for non-model organisms. The results promise to be significant to biocontrol designs in pest species, perhaps not by straight homology, but definitely as a set of design issues that should be accounted for.

My major suggestion is to improve the clarity of writing when describing the designs by being more explicit. Doing this will underscore the elegance of the system the authors have developed (comments 1 to 3 below specifically relate to this).
(1) Fig 2C classifies gene drive performance four ways and I'd like the explanation of how these outcomes are arrived at to be crystal clear. I believe it is mostly achieved by counting the number of each of the 14 phenotype of flies shown in fig2B. Am I right in deducing that the blue (drive conversion efficiency) category in Fig2c is the number of red eyed females with yellow bodies? And the salmon colour is those that are yellow bodies but not red eyed? How exactly is the green category calculated? And if the somatic level (purple) is estimated by the number of mosaics – what is the quantitative relationship with the shades of purple? Can the 14 flies of figure 2B clustered so they link directly to the categories of 2c?

(2) Figures 2D and 2E reports 'various cell types'. Is that the 259 cell types referred to in the legend of Figure 1 or the 63 of Table S2 or some other number? If the latter what happened to the rest?

(3) Also for figures 2D and 2E can you be explicit about which lines get put in the "high" and the "low" cut rate groups. I don't think you mean to synonymize 'cut rate' and 'conversion' rates which are entangled in the paragraph on lines 295 to 307. Are you excluding the 'moderate' (line 302), 'moderate to low' (line 306) and not detectable (line 307)? What are the dashed lines in these figures?

(4) To explain the lower drive conversion rates in males with the Act5C and Ubi-p63E promoters the authors suggest that their action early in development skews the repair pathway from HDR to NHEJ. Is there any supporting information for this in the literature?

(5) At several places it is noted that the regions chosen as "promoters" may actually be missing regulatory elements and wonder whether that also applies to the paragraph lines 614-622 on why sex specific promoters did not appear to be that different between sexes. I can't help but wonder whether enhancer trap experiments in Drosophila are informative to this issue?

(6) The unexpected finding that somatic expression is correlated with promoters expressed in male reproductive cells is interesting and the potential caveats of this finding are well discussed. However I wonder if any more direct experimental

tests of the phenomenon could be proposed? I am not insisting on more experimental work, but the finding is unexpected and somewhat puzzling, and important if true.

Minor issues:
• The sentence in the abstract beginning "In addition, we found that an in situ construct…' is too cryptic.
• Line 69 it should read 'if many gRNAs are required' rather than 'is many gRNAs are required'.
• Legend of fig1. There are not really "35 gene candidates" because they include Act5c and Ubi-pSE. Perhaps "35 genes".
• I think it worth specifying you are working with Drosophila melanogaster earlier than you do. It was only obvious to me when I came to w1118 on line 156 of the methods.
• Line 77 consider replacing "in all or nearly all cells" with "a subset of cells" for clarity; i.e. contrast more directly with the previous phrases.

(Remarks on code availability)

Reviewer #2

(Remarks to the Author)
The use of emerging single cell technologies in order to improve the efficacy of gene drives through germline-restricted expression of Cas9 is an emerging and likely highly fruitful avenue of research. Therefore this piece of work is timely and relevant and likely to add substantially to the development of new and better gene drive systems.
Overall I found the work well done, and clearly reported. In the body of the report below I make several suggestions that I think should improve the impact and reliability of the work.
For plasmid construction - I would request that previously developed plasmids are briefly described, that confirmation is given as to whether new plasmids were whole plasmid sequenced - and that all sequences are deposited in a suitable data repository such as Genbank or Addgene. The current data files are located in a github repository only with no permanent doi.
Drive performance assays were carried out by first crossing male Cas9 to female gRNA before bisex crosses to w118. For grandparent crosses - sex of Cas9 bearing individual can be important - can this be briefly discussed - or confirmed that this was to reduce maternal effects?
For the statistical analyses - the accompanying scripts on github work have filepaths hard coded to the original users OS and do not include the raw data files - making them unreproducible. This should be corrected and the resulting analysis archived with a doi.
Similarly data obtained from the Fly Cell Atlas - could this data be mirrored somewhere to guarantee persistent access?
I found it difficult to understand the logic behind the cutoffs for determining high-functioning and low functioning cut rate/gene drive genes. Without further context these appear largely arbitrary - and I would expect a much clearer rationale as to why these values can be altered to be different for males and females. This study was not preregistered so it becomes impossible to determine if these thresholds were agreed before or after data collection and initial analysis.
Looking at the supplemental figures - it would appear that some of these low performing genes have uniformly low expression rates - so is there a risk of just conflating with general expression levels? I would like to see a strong justification for why continuous regression based analysis cannot be used other than - to increase effect sizes.
The Wilcoxon analysis is ok - but of course uses rank ordered expression only - was any consideration given to a robust permutation analysis where standardised expression values could be used? Could the authors confirm if corrections for multiple testing across cell types was made?
I would query the language used around comparing conversion rates across yellow and cinnibar. Essentially the pattern of association cannot be replicated with another genetic target - focused discussion on how "high peforming genes" remain consistent should be tempered with the broader finding.
The male-female asymmetry discussion is plausible but unsupported experimentally, and throughout some of the claims of broader application should be tempered as unproven. As the authors acknowledge, locus-specific and common regulatory elements may confound results in addition to species specific effects.

(Remarks on code availability)
The code is not reproducible in its current form - it does not contain the supporting data and is hardcoded to the original OS and file locations

Reviewer #3

(Remarks to the Author)
The work submitted by the authors describes an effort to identify promoters that improve gene drive performance. This is an important line of investigation, as gene drives are powerful tools for propagating specific genetic traits through wild populations and therefore hold significant potential for applications such as the control of agricultural pests and disease vectors, as well as for conservation purposes. One such system, the Cas9-based homing gene drive, has produced encouraging results in malaria vectors such as Anopheles gambiae and Anopheles stephensi, but has proven difficult to implement in non-anopheline species, particularly with respect to achieving high homing efficiency. It has long been

hypothesised, as also noted by the authors, that there exists a narrow temporal and/or quantitative window of Cas9 expression that is optimal for efficient homing.

In this context, the authors' attempt to address this challenge by integrating existing single-nucleus RNA-seq data with experimental analyses of transgenic strains expressing Cas9 under the control of up to 35 different promoters represents a valuable contribution. In addition, the authors have taken care to integrate all constructs into the same genomic locus, thereby controlling for positional effects that could otherwise influence transgene expression patterns and levels.

However, a major concern is the substantial inconsistency between Cas9 expression outcomes and the reported expression patterns and levels of the corresponding endogenous promoter genes. For example (more details below), approximately 50% of promoters associated with genes classified as male-gonad-specific based on snRNA-seq data nonetheless resulted in significant homing, maternal deposition, and somatic phenotypes in female drive heterozygotes. This discrepancy is concerning, particularly because the manuscript relies heavily on the assumption that snRNA-seq expression profiles accurately predict the spatial and quantitative expression of Cas9 when driven by the corresponding promoters. The data presented suggest that this assumption does not consistently hold.

While I believe this work addresses an important question and has the potential to make a meaningful contribution to the field, there are additional major issues, outlined below, that must be resolved before the conclusions and claims of the manuscript can be fully supported.

Major comments:
Figure 2C, and consequently 2D: if I understand this correctly, the germline resistance rate = males that did not inherit the drive but appear yellow. However, I don't think you can rule out that this phenotype could be a result of maternal deposition of Cas9 and/or sgRNA from their mother, especially in those crosses which produced rather high levels of embryonic resistance. Most of these germline cut rates are likely overestimation of germline cut rate, mixed up with maternal deposition. Consequently, you may actually be comparing high-low maternal deposition group in 2D and it makes sense that you are getting predominantly female germline and reproductive somatic cells in the top right quadrant.

Figure 3E: According to Fly Cell Atlas, these genes are highly active in the male germlines and/or reproductive somatic cells but are inactive in the female germline and reproductive somatic cells: CG4415, ATPsynepsilonL, RpL22-like, CG13577, CG11929, CG12477, CG15262, CG42854, CG6324, SkpE, CG1894, CG14357. However, all these promoters resulted in >50% conversion rate in females, indicating that the endogenous gene expression pattern does not match the Cas9 expression pattern as a transgene. This is a significant issue to the entire manuscript as it assumes that Cas9 expression patterns largely mimic their promoter gene, which is evidently not the case here. Looking back at Figure 2C now, there are again many male-specific promoters with not much endogenous somatic expression, driving high conversion, somatic, and embryo resistance rates in females. This significantly reduces the confidence that these Cas9 lines are actually mimicking endogenous expression at all, let alone using this assumption to infer the right promoters. One could almost conclude from the data that the conversion rate (largely similar across almost all crosses) could be the result of integrating the Cas9 cds into the location in chromosome 2R and that the surrounding context causes the conversion.

Lines 49-51: I don't think overall drive effectiveness is the main issue for deployment, more due to regulations. Also, it has not been particularly challenging for Anopheline species. Several groups have published near 100% homing rates in Anopheles species.

Figure 4B: Because the yellow system cross was done with female drive heterozygotes, can you cleanly distinguish somatic phenotype that resulted from maternal deposition vs somatic leaky expression? Looking back at Figure 2C, how did crosses have high embryonic resistance but low somatic level?

Line 543-544: I'm afraid this is the Achilles' heel of the study "but also, through integration with single-cell transcriptomic data,". The data indicates (see comment about Figure 3E) about half of the time, scRNA-seq results of endogenous gene don't match the expression pattern of Cas9 driven by the same gene promoter. And the difference is not just a difference in intensity, but huge differences like expressing in females when the endogenous gene is male-specific.

Minor comments:

Line 56: 'copy' rather than 'insert' would be more accurate here.

Line 69: typo: 'if many gRNAs'.

Line 87: an ideal homing gene drive.

Line 95: sufficient proportion of homozygous, therefore sterile…

Line 99: one of the articles cited here (Hammond et al.) demonstrates large cage trials that succeeded in elimination cage population. Is this an intended citation here?

Line 108-110: the zpg strain in citation 41 was not found to exhibit fitness costs due to somatic expression, but due to paternal deposition.

Line 114-115: all citations point to Aedes aegypti, what about other non-model species?

Line 102-129: it is probably worth mentioning the CHIROS concept introduced by Ang et al. 2025 (https://doi.org/10.1016/j.cois.2025.101373) and the recently published single-nuclei atlas for Aedes aegypti (https://doi.org/10.1016/j.cell.2025.10.008) in these sections.

Line 126: it is not clear what the authors meant in 'what kinds of differences may be found between such data and Cas9 activity when driven by 126 promoters from native genes'.

Figure 2B: it would be good either here or somewhere in the text to explain how conversion, germline resistance, and embryonic resistance rates are determined.

Also for Figure 2C: Can you add a key to indicate which of these promoters are sex-specific, both sex, and constitutive?

Also for 2C: Can you describe somewhere how you determine the somatic levels? What % is low and what % is high?

Again for 2C: Which rhino construct is this?

Figure 3A: Does the lighter shade in the gRNA mean something?

Figure 4: It will be helpful to remind readers in the legend that this is derived from the yellow drive system. For consistency, I'd suggest changing 'high and low' to 'low and high' in the legend.

Line 436-438: I could be missing something here but my understanding from Figure 4B is the opposite i.e. high expression was positively correlated with somatic expression. This also contradicts lines 641-642 of the manuscript.

Line 457-461: It is not clear how these two cell types are singled out in the analyses in Figure 5A-B).

Line 486: typo (previously)

Line 464: It would be good to mention which three kinds in the text even if it is mentioned in Figure 5.

Line 466-467: It is not clear which part of the results in Figure 5C lead to the conclusion made in these two lines. Is it the fact that the green data points are all quite different to the orange data points? But if that was the case, there is almost no difference between the green and purple data points, the latter has low conversion and low embryo resistance.

Line 455-497: I think this section quite hard to follow and may need some rewriting for better clarity. At the moment it has many ideas/results but not enough context to enable a good flow of the idea.

Line 489-490: What about egg-to-pupa survival rate of the progeny? Did you measure them?

Line 496-497: Could the multi-sgRNA in Rpl35A have anything to do with this?

Line 517-518: Can you comment on why rhino was picked for this and not other promoters?

Line 574-575: Don't think I can agree with this.

Line 606-608: One successful example would be the nanosD strain in An. gambiae (https://academic.oup.com/g3journal/advance-article/doi/10.1093/g3journal/jkaf246/8305671?searchresult=1).

(Remarks on code availability)
I don't feel skilled enough to review the code.

Version 1:

Reviewer comments:

Reviewer #1

(Remarks to the Author)
The amended manuscript is improved. Figure s2 helps significantly. I find the term 'embryo resistant rate' confusing but once I have understood it is a proportion of female flies without the cas9 gene that destroy yellow (Ry) out of the total female flies without cas9 then it makes some sense. It is not clear to me why mosaics (Rm) don't count to the numerator. Additionally I suppose heterozygotes for dsRED cannot be distinguished from homozygotes at this stage?

I also note that snRNA-Seq reflects an "On" switch, but manipulating the "Off" switch that determines transcript and protein half life might need similar scrutiny in the future.

(Remarks on code availability)

Reviewer #3

(Remarks to the Author)
I thank the authors for their thorough and thoughtful responses to the concerns raised in my previous review. I am satisfied that these issues have now been adequately addressed.

(Remarks on code availability)

Open Access This Peer Review File is licensed under a Creative Commons Attribution 4.0 International License, which permits use, sharing, adaptation, distribution and reproduction in any medium or format, as long as you give appropriate credit to the original author(s) and the source, provide a link to the Creative Commons license, and indicate if changes were made.
In cases where reviewers are anonymous, credit should be given to 'Anonymous Referee' and the source.
The images or other third party material in this Peer Review File are included in the article's Creative Commons license, unless indicated otherwise in a credit line to the material. If material is not included in the article's Creative Commons license and your intended use is not permitted by statutory regulation or exceeds the permitted use, you will need to obtain permission directly from the copyright holder.
To view a copy of this license, visit https://creativecommons.org/licenses/by/4.0/

In the following, reviewer comments are in bold, and our responses are in plain text.

Reviewer #1 (Remarks to the Author):

The manuscript of Wu et al. identifies genes by their expression patterns in publicly available single-cell transcriptomic data sets, takes likely-promoter regions and uses them to express cas9. Thirty-five such constructs are then tested for attributes relevant to 'gene drive' technology: the efficiency at which cut and home in the germline, their cutting rate in the soma, the rate at which different classes of resistance arise. They do this among multiple gene drive designs that their impressive group has previously developed. While their overall conclusion that the timing of cas9 expression matters is unsurprising, the value of this paper is in the details, and how they differ between genetic biocontrol designs.

I found the introduction well structured around three 'aspects' of gene drive performance (conversion rates, resistance alleles, fitness costs) and I appreciated that the Discussion provided guidance to future research into finding promoters for non-model organisms. The results promise to be significant to biocontrol designs in pest species, perhaps not by straight homology, but definitely as a set of design issues that should be accounted for.

We thank the reviewer for these kind comments.

My major suggestion is to improve the clarity of writing when describing the designs by being more explicit. Doing this will underscore the elegance of the system the authors have developed (comments 1 to 3 below specifically relate to this).
(1) Fig 2C classifies gene drive performance four ways and I'd like the explanation of how these outcomes are arrived at to be crystal clear. I believe it is mostly achieved by counting the number of each of the 14 phenotype of flies shown in fig2B. Am I right in deducing that the blue (drive conversion efficiency) category in Fig2c is the number of red eyed females with yellow bodies? And the salmon colour is those that are yellow bodies but not red eyed? How exactly is the green category calculated? And if the somatic level (purple) is estimated by the number of mosaics – what is the quantitative relationship with the shades of purple? Can the 14 flies of figure 2B clustered so they link directly to the categories of 2c?

We have added new Figure S2 in the Supplementary Information, which provides a phenotype to performance parameter schematic for the *yellow* gene drive system. It should allow questions like these to be understood more clearly. This figure and Figure 2 are updated to included clearer phenotype images as well.

(2) Figures 2D and 2E reports 'various cell types'. Is that the 259 cell types referred to in the legend of Figure 1 or the 63 of Table S2 or some other number? If the latter what happened to the rest?

Our analysis of cell type specific differential expression was consistently performed across 258 annotated cell types**.** The missing category was labeled as **“**unknown” in the original single cell transcriptomic dataset and was therefore excluded from subsequent analyses. We added a sentence in the third paragraph of the “Cell type-specific differential expression analysis” section in the methods to clarify this point.

The reason that only 63 cell types are listed in Table S2 is that these cell types were statistically significant and ranked among the top results. To label these different cell types clearly within the limited space of the figures, we abbreviated their names. We have now added a note to the table to clarify that the cell types listed represent only a subset of all tested cell types.

(3) Also for figures 2D and 2E can you be explicit about which lines get put in the “high” and the “low” cut rate groups. I don’t think you mean to synonymize ‘cut rate’ and ‘conversion’ rates which are entangled in the paragraph on lines 295 to 307. Are you excluding the ‘moderate’ (line 302), ‘moderate to low’ (line 306) and not detectable (line 307)? What are the dashed lines in these figures?

Regarding the classification of the high and low groups, we provide a full description of all grouping criteria in the second paragraph of the “Cell type-specific differential expression analysis” section in the Methods. To make this easier for readers to follow, we have also added the grouping criteria in a simple and explicit form to the legends of all volcano plots.

We did not exclude the “moderate,” “moderate to low,” or “not detectable” categories simply based on those descriptive labels. The labels used in the Results section summarize promoter performance, whereas the “high” and “low” groups used in the differential expression analyses were defined separately using explicit quantitative cutoffs. These cutoffs were chosen to maximize the contrast between groups while retaining as many promoters as possible in each group, thereby improving the interpretability and statistical power of the analysis.

In addition, the statement “The horizontal dashed line indicates the threshold for $p < 0.05$, and the two vertical dashed lines indicate the thresholds for $|\Delta\log2(\text{TPM}+1)| > 1$” has now been added to the legends to clarify the meaning of the dashed lines.

We are treating apparent germline cut rate (drive conversion + nonfunctional germline resistance) and drive conversion rate separately, with entirely sperate analyses in the figures. In lines 295-307, we often refer to these separately as well. We’ve checked the paragraph, and it looks like they are used correctly.

(4) To explain the lower drive conversion rates in males with the Act5C and Ubi-p63E promoters the authors suggest that their action early in development skews the repair pathway from HDR to NHEJ. Is there any supporting information for this in the literature?

Regarding drive performance, several previous studies (Du et al., 2024; Terradas et al., 2023) have shown that, in multiple *Drosophila* homing gene drive systems, female drive conversion often exceeds male drive conversion, although this pattern is not universal across all drive designs and target loci. The same trend can also be observed in the cinnabar system in our study. Although we have not found direct evidence demonstrating that double strand breaks generated during early male germline development are preferentially repaired through end-joining rather than HDR, we speculate that differences in the developmental programs of the male and female germlines in *Drosophila* may provide a reasonable explanation.

Under constitutive promoters such as Act5C and Ubi-p63E, Cas9 is likely to be expressed broadly at relatively early developmental stages and to induce DNA cleavage early during germline development. In the female germline, cells undergo a limited number of proliferations before entering a developmental state associated with meiosis and homologous recombination. Therefore, even when Cas9 is expressed broadly, its activity is still likely to overlap with a window that is relatively beneficial for HDR. By contrast, the male *Drosophila* germline lacks canonical meiotic recombination, and its early development is characterized primarily by the continued proliferation of germline stem cells and spermatogonial cells. If constitutive promoters regulate widespread Cas9 cleavage during these earlier premeiotic stages, the resulting double strand breaks are likely to arise in a window that is less beneficial for HDR. Under such conditions, although cleavage itself may still occur efficiently, repair may preferentially proceed through end-joining pathways, thereby producing the high cut, low conversion phenotype observed in our study.

One indirect line of evidence to support this is that drive conversion appears to be higher in germline cells than in somatic cells, even when total cut rate is high:
Li Z, Marcel N, Devkota S, Auradkar A, Hedrick SM, Gantz VM, Bier E. CopyCatchers are versatile active genetic elements that detect and quantify inter-homolog somatic gene conversion. *Nat Commun*, 12, 1–12, 2021.
However, this study doesn't alone explain male-female differences, just the notion that cutting in the germline is better than the soma in general.

(5) At several places it is noted that the regions chosen as "promoters" may actually be missing regulatory elements and wonder whether that also applies to the paragraph lines 614-622 on why sex specific promoters did not appear to be that different between sexes. I can't help but wonder whether enhancer trap experiments in Drosophila are informative to this issue?

Yes, this may also apply to our discussion of why sex specific promoters did not show as large a difference between the two sexes as expected. We used promoter regions of limited length. Although we made every effort to include all proximal transcription factor-binding regions when defining the promoter fragments, we were not able to fully capture additional regulatory information such as distal enhancers, chromatin context, or even regulatory elements located in the 3′ UTR.

Enhancer trapping is indeed a useful approach for detecting and validating whether the promoter regions we selected may be missing distal regulatory elements. However, it cannot by itself directly identify which specific elements are absent from these particular promoter constructs. If the goal were to fully recapitulate the expression pattern of a given gene, one possible strategy would be to insert Cas9 *in situ* immediately downstream of the gene's 5′UTR, thereby placing it under endogenous regulatory control (as illustrated in Fig. 6B). Either way, this doesn't seem to be a pattern related to specific promoter elements that might be missing something. It was a nearly universal pattern in our constructs, not likely to be explained by a modest fraction of our contracts missing an enhancer, unless distant enhancers are in fact extremely common.

Thus, we believe the more important explanations are likely the following two points. First, in processing the Fly Cell Atlas data, we used the median expression value to represent gene expression levels across different cell types. Although this approach helps reduce the influence of outliers and improves the overall robustness of the analysis, it may also compress the true dynamic range of expression and obscure transient high expression states that could be critical for drive performance. Second, the Fly Cell Atlas provides single cell expression information at the RNA level, whereas what we measured was drive performance at the level of the whole fly. The latter is influenced not only by transcriptional output, but also by post transcriptional regulation, translational efficiency, protein stability, protein persistence, and the timing of DNA cleavage and repair (including how long is available for cleavage before proceeding to the next cells stage, a critical consideration). Therefore, even if a gene shows a particular expression pattern in the single-cell transcriptomic data, the ultimate performance of Cas9 driven by the corresponding promoter does not necessarily have to match that pattern exactly. However, we would like to emphasize that our differential analysis does not depend on these constructs perfectly recapitulating the endogenous expression patterns of the corresponding genes. As long as these promoter fragments retain biologically meaningful regulatory information, this type of comparative analysis remains valid and informative.

(6) The unexpected finding that somatic expression is correlated with promoters expressed in male reproductive cells is interesting and the potential caveats of this finding are well discussed. However I wonder if any more direct experimental tests of the phenomenon could be proposed? I am not insisting on more experimental work, but the finding is unexpected and somewhat puzzling, and important if true.

Although this result suggests an interesting and perhaps previously unrecognized relationship, as noted in the Discussion, we must acknowledge that biases introduced during promoter selection may have influenced this result to some extent. We therefore believe that the key to testing this phenomenon is to establish a more balanced and less biased promoter testing framework.

Specifically, we could select a set of candidate promoters based on scRNA-seq data that represent different tissue specificities, while ensuring that the relative representation and specificity strength

of each promoter class are as balanced as possible. This would help reduce the bias arising from the enrichment of germline specific promoters in our promoter set (which was chosen to focus on germline promoters rather than be balanced) and would allow a more direct evaluation of the true relationship between somatic leakage and expression patterns.

Given the importance of this question and its potential mechanistic implications, as well as the large amount of time invested in expanding our set of promoter sufficiently to make it balanced, we believe that such a work would be an entire project by itself and thus beyond the scope of this study. We have added a brief description of this experimental validation strategy to the corresponding part of the Discussion.

Minor issues:
• The sentence in the abstract beginning "In addition, we found that an in situ construct…' is too cryptic.

We revised this sentence in the Abstract to make the meaning more explicit. It now reads: "In addition, we found that somatic expression of Cas9 with the rhino promoter is significantly reduced when integrated at the rhino native genomic locus, underscoring the importance of endogenous gene regulation."

• Line 69 it should read 'if many gRNAs are required' rather than 'is many gRNAs are required'.
• Legend of fig1. There are not really "35 gene candidates" because they include Act5c and Ubi-pSE. Perhaps "35 genes".

Both of these have been fixed.

• I think it worth specifying you are working with Drosophila melanogaster earlier than you do. It was only obvious to me when I came to w1118 on line 156 of the methods.

We now specify in the Abstract and end of the Introduction that the study was performed in *Drosophila melanogaster*.

• Line 77 consider replacing "in all or nearly all cells" with "a subset of cells" for clarity; i.e. contrast more directly with the previous phrases.

It's not entirely a contrast (because resistance can still form in all cells in a series of cleavage events), but we agree that mentioning it could happen to a subset is important too. We've revised the wording.

Reviewer #2 (Remarks to the Author):

The use of emerging single cell technologies in order to improve the efficacy of gene drives through germline-restricted expression of Cas9 is an emerging and likely highly fruitful avenue of research. Therefore this piece of work is timely and relevant and likely to add substantially to the development of new and better gene drive systems.
Overall I found the work well done, and clearly reported. In the body of the report below I make several suggestions that I think should improve the impact and reliability of the work.

We thank the reviewer for these comments.

For plasmid construction - I would request that previously developed plasmids are briefly described, that confirmation is given as to whether new plasmids were whole plasmid sequenced - and that all sequences are deposited in a suitable data repository such as Genbank or Addgene. The current data files are located in a github repository only with no permanent doi.

In the revised manuscript, we have added brief descriptions of the previously constructed plasmids in the first paragraph of the "Plasmid construction" section in the Methods. Specifically, SNc9CGnG contains a Cas9 expression cassette driven by the *CG11052* promoter and *nanos* 3′UTR, together with a 3×P3-EGFP marker. TTChsp70c9 contains a Cas9 expression cassette driven by the *Hsp70Bb* promoter. BHDcN1 contains a nanos promoter/nanos 3′UTR-driven Cas9 cassette, a 3×P3-dsRed marker, and a gRNA cassette targeting *cinnabar*. BHDabg1 contains gRNA cassettes targeting the intergenic region between *gapdh1* and *CG12824* on chromosome 2R. We now include these maps in our GitHub Repository, which will be archived on Zenodo (with a DOI), upon acceptance of the manuscript.

Most of the newly constructed plasmids were verified by whole-plasmid sequencing. For a small subset of plasmids generated at an earlier stage of the project, whole-plasmid sequencing was not performed because third-generation long-read sequencing was not yet widely accessible in China at that time. However, all of these plasmids were verified by Sanger sequencing across the key engineered regions. Based on these validation steps, we are confident that the plasmids used in this study were constructed correctly.

Drive performance assays were carried out by first crossing male Cas9 to female gRNA before bisex crosses to w118. For grandparent crosses - sex of Cas9 bearing individual can be important - can this be briefly discussed - or confirmed that this was to reduce maternal effects?

Yes, the sex of the individual carrying Cas9 in the grandparent cross can indeed affect the experimental outcome. The main reason is that maternal inheritance of Cas9/gRNA may result in deposition of these into the embryo, thereby promoting early embryonic cleavage and leading to the formation of resistance alleles. We've found that both need to be deposited to get such resistance that blocks drive. However, Cas9 deposition could potentially persist until new gRNAs

are expressed, changing the timing of cleavage for future drive conversion. For this reason, in the grandparent cross we used male Cas9 flies and female gRNA flies in order to minimize maternal Cas9 deposition effects on the subsequent drive performance assays. In the revised manuscript, we added a brief explanation of this point in the first paragraph of the "Drive performance assays" section in the Methods.

For the statistical analyses - the accompanying scripts on github work have filepaths hard coded to the original users OS and do not include the raw data files - making them unreproducible. This should be corrected and the resulting analysis archived with a doi.
Similarly data obtained from the Fly Cell Atlas - could this data be mirrored somewhere to guarantee persistent access?
Reviewer #2 (Remarks on code availability):
The code is not reproducible in its current form - it does not contain the supporting data and is hardcoded to the original OS and file locations

We have corrected this issue in the revised version. The analysis scripts were updated to remove OS-specific hard-coded file paths and now use relative paths. To improve reproducibility and ensure persistent access, we deposited the processed Fly Cell Atlas data used in this study, together with the bulk RNA-seq data, in Zenodo under DOI: 10.5281/zenodo.18996871. The analysis scripts and plasmid maps are currently available in our GitHub repository (https://github.com/jchamper/Single-Cell-Promoters-Homing). Upon acceptance of the manuscript, we will archive the GitHub repository on Zenodo and add the corresponding DOI to the final manuscript.

I found it difficult to understand the logic behind the cutoffs for determining high-functioning and low functioning cut rate/gene drive genes. Without further context these appear largely arbitrary - and I would expect a much clearer rationale as to why these values can be altered to be different for males and females. This study was not preregistered so it becomes impossible to determine if these thresholds were agreed before or after data collection and initial analysis.

The thresholds were not set arbitrarily. In each analysis, the guiding principle for threshold was to maximize the contrast between groups while still retaining a sufficient number of promoters, thereby constructing biologically meaningful extreme comparison groups.

We note that there is no natural cutoff in this setting, and the "good" versus "bad" categories used for grouping are not absolute definitions. Different thresholds were used for males and females because the distributions of drive performance differ between the two sexes. Overall, female conversion rates were generally higher than male conversion rates. Therefore, applying identical numerical cutoffs to both sexes would have resulted in too few promoters in the low conversion group for females, leading to a less balanced comparison. We now clarify this point in the Methods section.

Other cutoffs are already explained in detail, such as embryo resistance analysis only considering samples that had at least moderate germline activity (required for any maternal deposition).

Looking at the supplemental figures - it would appear that some of these low performing genes have uniformly low expression rates - so is there a risk of just conflating with general expression levels? I would like to see a strong justification for why continuous regression based analysis cannot be used other than - to increase effect sizes.

We agree that the low performing groups may indeed include some genes with relatively low overall expression levels. For the female bias, embryo resistance, and somatic expression analyses, we applied minimum drive conversion thresholds before defining comparison groups. As a result, promoters with generally low expression were not assigned to the low performance groups in a way that would confound these analyses. For the cut rate and drive conversion analyses, we acknowledge that promoters with lower overall expression could influence the differential comparisons to some extent. Accordingly, we interpret these results as reflecting a composite signal shaped by both overall expression and cell type specific expression features. Nevertheless, this analytical framework still allows us to identify biologically meaningful expression patterns associated with different drive performance.

Our decision not to use continuous regression was not based simply on increasing effect size. Although we systematically tested 35 promoters across two drive systems (representing a relatively large experimental effort for gene drive study), the sample size is still limited for continuous regression from a statistical modeling perspective. In addition, the phenotypic metrics themselves are bounded and unevenly distributed, and we did not expect the relationship between expression pattern and drive performance to be strictly linear (a clear example is our finding that optimal drive performance requires Cas9 expression within a relatively narrow range), especially when expression passed the threshold that would result in nearly 100% cleavage rates. Under these conditions, a global continuous regression model would be more susceptible to noise, outliers, and nonlinearity, whereas extreme-group comparisons are better suited to identifying clearly contrasting promoter classes and enabling more robust comparisons at the current scale of the dataset. If we are able to establish a higher throughput framework for measuring promoter drive performance in the future, it may then become feasible to apply continuous regression or other multivariable modeling approaches to a substantially larger promoter set.

The Wilcoxon analysis is ok - but of course uses rank ordered expression only - was any consideration given to a robust permutation analysis where standardised expression values could be used? Could the authors confirm if corrections for multiple testing across cell types was made?

We agree that permutation analysis could be a valuable complementary approach to consider in future work. In the present study, we chose the Wilcoxon rank-sum test because, as a nonparametric

method, it is more robust and particularly well suited to the non-normality, between-group heterogeneity, and outliers commonly encountered in expression data. Our primary aim in this analysis was to determine whether different promoter groups showed systematic differences in cell type associated expression patterns, rather than to precisely model the magnitude of standardized expression differences. Therefore, given the current dataset size and the purpose of the analysis, we considered the Wilcoxon approach to be a relatively conservative and straightforward choice.

At the same time, we also understand the concern regarding the robustness of the differential analysis results. In our response to Reviewer 3, we did a simplified permutation-based analysis. Although this was not a formal permutation test, it was sufficient to show that the observed differences were not easily generated by random grouping and thus displayed a certain degree of robustness.

We did not apply multiple testing correction in this analysis. As noted above, although we systematically tested 35 promoters across two drive systems, which already represents a relatively large experimental effort for this type of study, the sample size remains limited from the perspective of statistical power. Under these conditions, stringent correction could substantially reduce sensitivity and lead to the loss of potentially meaningful biological signals. Because this analysis was intended primarily as an exploratory screen, we chose at this stage to retain potentially informative candidate signals. This means that some false positives may remain, but these candidates can be resolved through future experimental validation. In our view, prematurely eliminating potential true positives would represent the greater loss in the context of this study. We also note that when discussing results, we often use groups of samples (such as from similar cell types, which could itself complicate multiple testing correction) rather than individual samples, thus mitigating the effect of not having multiple testing correction in terms of conclusions drawn.

I would query the language used around comparing conversion rates across yellow and cinnibar. Essentially the pattern of association cannot be replicated with another genetic target - focused discussion on how "high peforming genes" remain consistent should be tempered with the broader finding.

We are not quite certain what the reviewer is saying here. In the Results section, we show that for female drive conversion, the results obtained from the *cinnabar* system is highly consistent with those from the *yellow* system, indicating that the influence of promoters on drive performance is likely generalizable across different gene drive systems. Of course, this is not guaranteed, and gRNA activity and genetic target also make important contributions. We've adjusted our wording to more clearly emphasize these points.

The male-female asymmetry discussion is plausible but unsupported experimentally, and throughout some of the claims of broader application should be tempered as unproven. As the authors acknowledge, locus-specific and common regulatory elements may confound results in addition to species specific effects.

We have revised the relevant Results section to temper the wording (specifically noting that this is speculation), so that the possible explanation more accurately reflects its certainty level.

Reviewer #3 (Remarks to the Author):

The work submitted by the authors describes an effort to identify promoters that improve gene drive performance. This is an important line of investigation, as gene drives are powerful tools for propagating specific genetic traits through wild populations and therefore hold significant potential for applications such as the control of agricultural pests and disease vectors, as well as for conservation purposes. One such system, the Cas9-based homing gene drive, has produced encouraging results in malaria vectors such as Anopheles gambiae and Anopheles stephensi, but has proven difficult to implement in non-anopheline species, particularly with respect to achieving high homing efficiency. It has long been hypothesised, as also noted by the authors, that there exists a narrow temporal and/or quantitative window of Cas9 expression that is optimal for efficient homing.

In this context, the authors' attempt to address this challenge by integrating existing single-nucleus RNA-seq data with experimental analyses of transgenic strains expressing Cas9 under the control of up to 35 different promoters represents a valuable contribution. In addition, the authors have taken care to integrate all constructs into the same genomic locus, thereby controlling for positional effects that could otherwise influence transgene expression patterns and levels.

However, a major concern is the substantial inconsistency between Cas9 expression outcomes and the reported expression patterns and levels of the corresponding endogenous promoter genes. For example (more details below), approximately 50% of promoters associated with genes classified as male-gonad-specific based on snRNA-seq data nonetheless resulted in significant homing, maternal deposition, and somatic phenotypes in female drive heterozygotes. This discrepancy is concerning, particularly because the manuscript relies heavily on the assumption that snRNA-seq expression profiles accurately predict the spatial and quantitative expression of Cas9 when driven by the corresponding promoters. The data presented suggest that this assumption does not consistently hold.

While I believe this work addresses an important question and has the potential to make a meaningful contribution to the field, there are additional major issues, outlined below, that must be resolved before the conclusions and claims of the manuscript can be fully supported.

We thank the reviewer for this thorough summary. We agree that it's an issue that transcriptome data does not directly determine drive performance, but it seems to be at least correlated, which is perhaps all the could be expected given the known importance of other factors such as genomic

correlation. We also note that genes we defined as having specific expression patterns based on the transcriptomic data, for example male specific genes, were not completely silent in female germline cells in the original single cell dataset, but instead often still showed some level of expression there. This observation is also consistent with our proposed idea of an ideal expression window. Our conclusions certainly won't be a silver bullet for drive performance, but perhaps could still help improve the design of future drives, reducing the number of iterations before acceptable performance is obtained.

Major comments:

Figure 2C, and consequently 2D: if I understand this correctly, the germline resistance rate = males that did not inherit the drive but appear yellow. However, I don't think you can rule out that this phenotype could be a result of maternal deposition of Cas9 and/or sgRNA from their mother, especially in those crosses which produced rather high levels of embryonic resistance. Most of these germline cut rates are likely overestimation of germline cut rate, mixed up with maternal deposition. Consequently, you may actually be comparing high-low maternal deposition group in 2D and it makes sense that you are getting predominantly female germline and reproductive somatic cells in the top right quadrant.

We agree that maternal deposition of Cas9 and/or gRNA could potentially contribute to the yellow phenotype in some non-drive male progeny. It remains unclear exactly how much this occurred. The *yellow* system is X-linked. Any maternally deposited Cas9/gRNA in these males can act only after being present in the germline with Cas9. Most of the time, if an oocyte carried sufficient Cas9/gRNA for maternal deposition, cleavage of the wild-type allele would already have occurred in the female germline prior to fertilization. This is supported by our data showing that embryo resistance only takes place when the total germline cut rate is nearly 100% (shu represents a minor exception, with some embryo resistance taking place despite some wild-type alleles being present in males at the end).

This doesn't prove that embryo resistance is not important in our "germline cut rate" measurement, but emphasizes how closely correlated the two are. It may also be possible in some cases for Cas9 to be expressed relatively late during oogenesis but still at a high level. It could then avoid germline cleavage while being heavily deposited into embryos, cutting them at a high rate. Such cases would lead to some overestimation of the germline resistance rate.

Importantly, this does not change the main biological interpretation of Fig. 2D. Both true maternal germline resistance formation and resistance arising from maternally deposited Cas9/gRNA are expected to be associated with elevated female germline expression of the promoter-associated genes. Therefore, although the absolute values in Fig. 2C may include some contribution from maternal deposition, the association in Fig. 2D with female germline and reproductive cell expression remains biologically consistent. Further, we do see some differences between this chart and our subsequent analysis of just embryo resistance (which is sometimes low even when total germline cut rate is high).

To a certain extent, this can be examined. All of our constructs with embryo resistance had moderate amounts of mosaicism in addition to full yellow phenotype. This means that if there is moderate to high embryo resistance and a decent number of wild-type alleles that are not cut in the germline, we can expect to see some mosaic males. However, only four of our many promoters lines produced any mosaic males, and CG11929 and CG15262 produced only two each, an almost negligible amount. Interesting, Cyp6a19 produced a fair number of these males, and fs(1)M3 produced a lower but still significant number. These are remarkable for having relatively low drive conversion but higher germline resistance than expected. It is likely that they indeed have a lot of Cas9 expression, but too late for germline cleavage events (and thus overestimates of germline cleavage rates).

In the manuscript, we have made some revisions to bring up this possibility and note how closely correlated germline cutting and embryo resistance are. We state that: "One caveat of this data is that embryo resistance can act on any wild-type allele, including one that avoided germline cleavage. Thus, our germline nonfunctional resistance allele formation rates measured from male progeny and total germline cut rates may be overestimates if some of these formed in the embryo. Given that the Cas9 would have been expressed in the germline in the first place, it is likely that these would only be small overestimates, except perhaps in a fraction of promoters if Cas9 was expressed occurred very late in the germline. Because moderate to high embryo resistance is also associated with mosaic outcomes, we'd expect to see mosaic male progeny (in additional to full yellow phenotype males) if wild-type alleles were uncut in female germline but later affected by embryo activity. Most constructs produced none of these males. They only showed up in appreciable fractions for Cyp6a19 and to a lesser extent fs(1)M3. For these, our germline cleavage rates are likely overestimates. It is possible that these promoter lead to high levels of Cas9 expression in the late germline where there is insufficient time to cleavage all wild-type alleles, resulting in both high maternal deposition and cleavage of maternal wild-type alleles in the embryo."

Figure 3E: According to Fly Cell Atlas, these genes are highly active in the male germlines and/or reproductive somatic cells but are inactive in the female germline and reproductive somatic cells: CG4415, ATPsynepsilonL, RpL22-like, CG13577, CG11929, CG12477, CG15262, CG42854, CG6324, SkpE, CG1894, CG14357. However, all these promoters resulted in >50% conversion rate in females, indicating that the endogenous gene expression pattern does not match the Cas9 expression pattern as a transgene. This is a significant issue to the entire manuscript as it assumes that Cas9 expression patterns largely mimic their promoter gene, which is evidently not the case here. Looking back at Figure 2C now, there are again many male-specific promoters with not much endogenous somatic expression, driving high conversion, somatic, and embryo resistance rates in females. This significantly reduces the confidence that these Cas9 lines are actually mimicking endogenous expression at all, let alone using this assumption to infer the right promoters. One could almost conclude from the data that the conversion rate (largely similar across almost all crosses) could be the

result of integrating the Cas9 cds into the location in chromosome 2R and that the surrounding context causes the conversion.

We believe that some difference between a gene's expression pattern in the single cell transcriptomic data and the drive performance regulated by its corresponding promoter are to be expected. It's not quite the "ideal" result for drive designers, but we don't think anyone expected things like genomic location to not affect overall performance. Our results still show correlations between drive performance and the transcriptome, even if not quite all the expected ones and not quite enough to pick with certainty a single optimal promoter on the first try. However, we believe that our results indicate that using good quality single-cell transcriptome data will allow good promoters to be identified more quickly.

Specifically, in this study, we selected predicted transcription factor-enriched regions from the relevant genes as candidate promoter fragments. As a result, these fragments may not fully capture the contributions of distal enhancers, silencers, chromatin structure, and other regulatory elements that affect gene expression. Further, in processing the Fly Cell Atlas data, we used the median expression value to represent gene expression levels across different cell types. Although this approach helps reduce the influence of outliers and improves overall robustness, it may also compress the true dynamic range of expression and obscure transient high expression states that may be critical for drive performance. In addition, single-cell RNA-seq data are inherently sparse and subject to dropout, so median based summaries may not fully reflect the true activity of some genes. Finally, the Fly Cell Atlas provides single cell expression information at the RNA level, whereas what we measured was drive performance at the level of the whole fly. The latter is influenced not only by transcriptional output, but also by post-transcriptional regulation, translational efficiency, protein stability, protein persistence, and the timing of DNA cleavage and repair. Therefore, even if a gene shows a particular expression pattern in the single cell transcriptomic data, the ultimate performance of Cas9 driven by the corresponding promoter does not necessarily have to match that pattern exactly.

Taken together, these considerations indicate that such differences are expected and biologically reasonable. We therefore next consider whether these differences would affect the validity of our analysis. Importantly, our analysis does not require that each promoter construct perfectly reproduce the endogenous expression pattern of its associated gene. The goal of our differential analysis was not to claim one-to-one equivalence between endogenous transcript abundance and Cas9 expression. Rather, our aim was to test whether promoter-associated genes linked to different drive performance show distinguishable cell type specific expression patterns. As long as the promoter fragments retain biologically meaningful regulatory information, this comparative framework remains valid and informative.

To consider this, we used a simplified permutation-based analysis to show that our promoter fragments retain biologically meaningful information. Specifically, we randomly shuffled the correspondence between genes and their single-cell expression profiles. The purpose of this control

was to illustrate what the same differential analysis would yield if the promoter-associated genes and the expression patterns used in the analysis were completely unrelated. As shown in the figure below in parts A, C, E, G, I, and K, randomization largely abolished the signals seen in the original analysis. Significance was markedly reduced, and the coherent enrichment of biologically relevant cell types was lost. By contrast, the analyses based on the original expression profiles (Fig. B, D, F, H, J, and L) had clear and meaningful patterns. These comparisons indicate that the observed results are not simply an inevitable output of the analytical pipeline, but instead depend on the genuine relationship between promoter-associated genes and their expression profiles. This supports the notion that, even if the promoter constructs do not perfectly recapitulate endogenous expression, they still retain sufficient biologically meaningful regulatory information for the comparative analysis to remain informative.

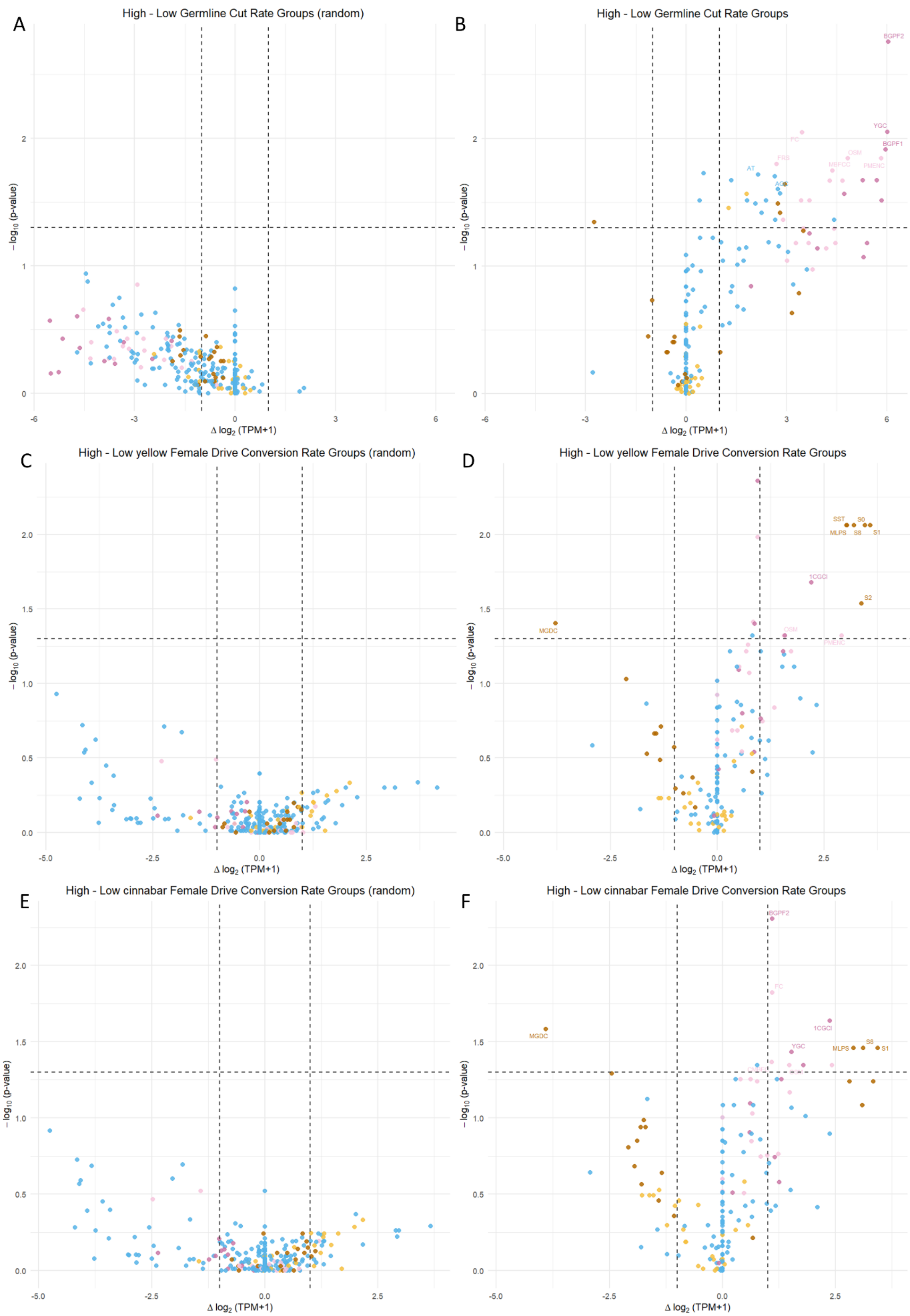
A
High - Low Germline Cut Rate Groups (random)
−log10 (p-value)
Δ log2 (TPM+1)
B
High - Low Germline Cut Rate Groups
BGPF2
YGC
BGPF1
FC
FRS
OSM
MBFCC
PMENC
AT
AGC
−log10 (p-value)
Δ log2 (TPM+1)
C
High - Low yellow Female Drive Conversion Rate Groups (random)
−log10 (p-value)
Δ log2 (TPM+1)
D
High - Low yellow Female Drive Conversion Rate Groups
SST
S0
MLPS
S8
S1
1CGCI
S2
MGDC
OSM
PMENC
−log10 (p-value)
Δ log2 (TPM+1)
E
High - Low cinnabar Female Drive Conversion Rate Groups (random)
−log10 (p-value)
Δ log2 (TPM+1)
F
High - Low cinnabar Female Drive Conversion Rate Groups
BGPF2
FC
1CGCI
MGDC
YGC
S8
MLPS
S1
−log10 (p-value)
Δ log2 (TPM+1)

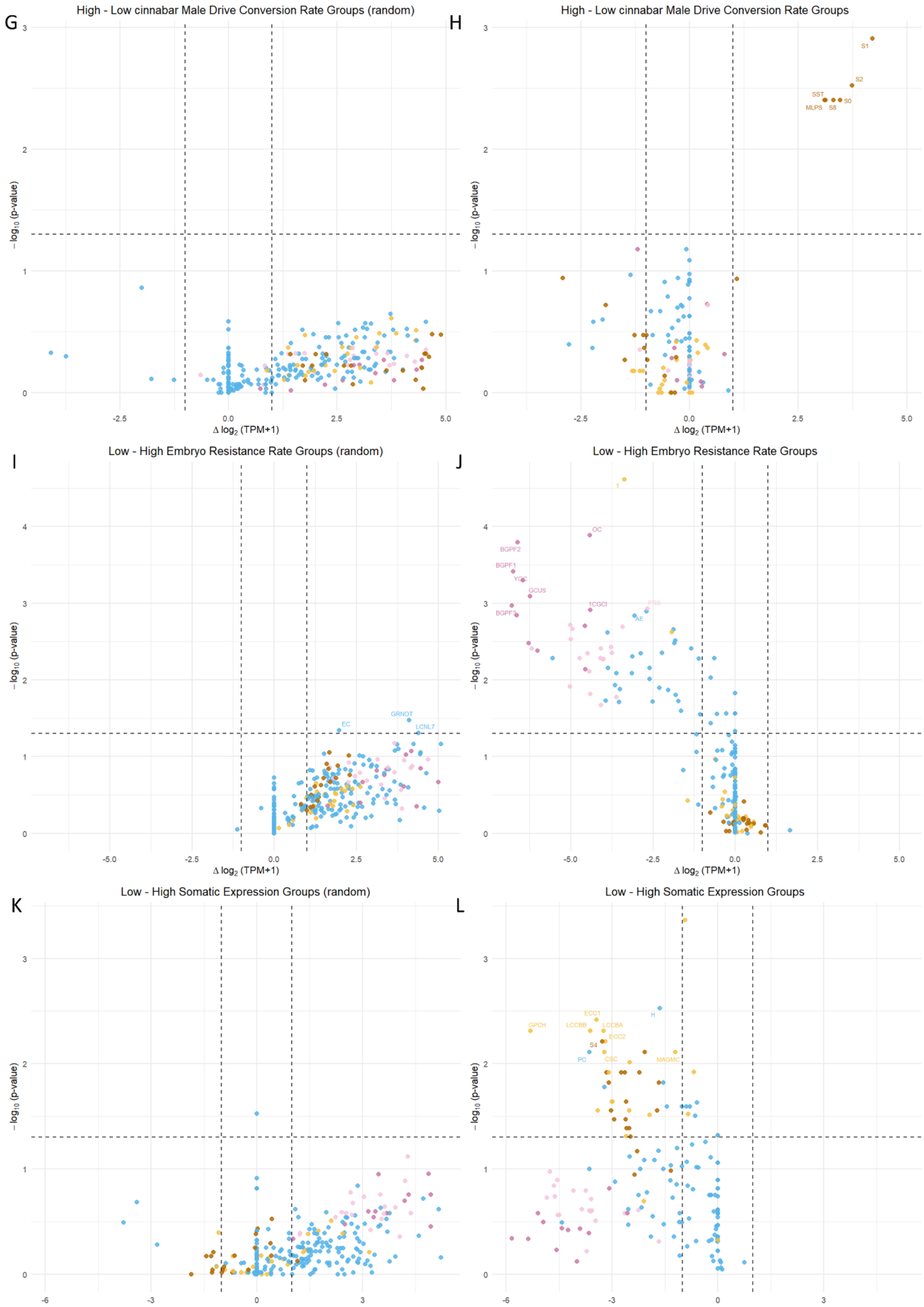
G
High - Low cinnabar Male Drive Conversion Rate Groups (random)
H
High - Low cinnabar Male Drive Conversion Rate Groups
S1
S2
SST
S0
MLPS
S8
I
Low - High Embryo Resistance Rate Groups (random)
EC
GRNOT
LCNL7
J
Low - High Embryo Resistance Rate Groups
T
OC
BGPF2
BGPF1
YGC
GCUS
1CGCI
FRS
AE
BGPF3
K
Low - High Somatic Expression Groups (random)
L
Low - High Somatic Expression Groups
ECC1
H
GPCH
LCCBB
LCCBA
ECC2
S4
PC
CSC
MAGMC
−log10 (p-value)
Δ log2 (TPM+1)

Differential cell type expression analysis of promoter associated genes for different performance groups. A-B. Differential expression of promoter-associated genes between high- and low-cut-rate groups across cell types: analysis using permuted single-cell expression profiles (A) and original single-cell expression profiles (B). Each point represents a single cell type from the Fly Cell Atlas dataset, with the horizontal axis indicating the difference in $\log_2(\text{TPM}+1)$ between high and low cut rate groups, and the vertical axis showing $-\log_{10}(p\text{-value})$ derived from Wilcoxon rank-sum tests. The horizontal dashed line indicates the threshold for $p < 0.05$, and the two vertical dashed lines indicate the thresholds for $|\Delta\log2(\text{TPM}+1)| > 1$. Top cell types with significant differences ($p < 0.05$ and $|\Delta\log_2(\text{TPM}+1)| > 1$) are labeled with their abbreviated names. **C-D.** Differential expression of promoter-associated genes between high and low female drive conversion groups in the *yellow* system across various cell types: analysis using permuted single-cell expression profiles (C) and original single-cell expression profiles (D). **E-F.** Differential expression of promoter-associated genes between high and low female drive conversion groups in the *cinnabar* system across various cell types: analysis using permuted single-cell expression profiles (C) and original single-cell expression profiles (D). **G-H.** Differential expression of promoter-associated genes between high and low male drive conversion groups in the *cinnabar* system across various cell types: analysis using permuted single-cell expression profiles (G) and original single-cell expression profiles (H). **I-J.** Differential expression of promoter-associated genes across various cell types between low and high embryo resistance rate groups across various cell types: analysis using permuted single-cell expression profiles (I) and original single-cell expression profiles (J). **K-L.** Differential expression of promoter-associated genes across various cell types between low and high somatic expression groups across various cell types: analysis using permuted single-cell expression profiles (K) and original single-cell expression profiles (L).

Finally, we do not think that the available data support the interpretation that drive conversion is determined primarily by the shared chromosome 2R integration site rather than by promoter identity itself. If the surrounding genomic context were the dominant factor, we would expect different constructs to show broadly similar outcomes. In reality, however, we observed variation among different promoters tested at the same integration site. In addition, female conversion rates were highly concordant between the *yellow* and *cinnabar* systems, supporting the view that promoter contributes meaningfully across different genetic backgrounds, rather than the observed effects being attributable solely to the chromosomal context. Our rhino results further indicate that genomic context does indeed affect expression, as *in situ* expression reduced somatic activity relative to the ectopically inserted constructs. On the other hand, our previous studies have shown more modest (but still existing) differences between expression at different genomic sites. Our overall conclusion supported by these results is that both promoter identity and genomic context jointly influence expression and drive phenotypes, not that promoter information itself lacks explanatory value.

Lines 49-51: I don't think overall drive effectiveness is the main issue for deployment, more due to regulations. Also, it has not been particularly challenging for Anopheline species. Several groups have published near 100% homing rates in Anopheles species.

We agree that regulatory, governance, and implementation considerations are important for whether gene drives can ultimately be deployed. We also agree that high drive conversion have been achieved in several *Anopheles* studies. However, it remains unclear whether the *Anopheles* studies have produced a gene drive that was technically ready for deployment, even up to today.

As an example, the Crisanti Lab produced a series of improved homing suppression drives from 2015-2024 for *Anopheles gambiae*. They initially failed due to functional resistance and high female fitness costs. Both of these were somewhat mitigated in a 2018 study that saw success in cages. However, additional testing indicated that functional resistance remained a problem, and modeling suggested that the remaining still medium fitness costs could also be problematic. A 2024 preprint partly solved functional resistance with multiplexing (it was also the study that showed that the 2018 study did not fully solve functional resistance), but not in a genomically stable way. It also seemed to further reduce fitness costs to accepted levels. At the same time, we and others showed better gRNA multiplexing methods. It is possible that Crisanti Lab made a drive in 2025 that would finally pass these laboratory tests, but such a drive is not published.

In another major example, the James Lab also made their first *Anopheles* homing modification drive in 2015. It usually failed due to fitness costs from the target site and resistance. A series of improvements made changes to the drive that allowed it to be mostly successful in cage populations (different Cas9 promoter, different target site). However, it is still unclear if the problem of resistance has been solved. They are only created at a low rate, but fitness costs in a natural environment might give them too big of an advantage over the drive (the drive targets an eye pigment gene with a single gRNA).

Thus, even in *Anopheles*, it remains unclear or at least unpublished if the technical problems have been solved. Furthermore, we do not think these high achieving results can be generalized across gene drive systems. Even within *Anopheles*, performance also depends on target locus and nuclease regulation. Outside *Anopheles*, performance has often been more limited. In other insects such as *Aedes* and *Culex* mosquitoes, drive conversion efficiency has remained far lower. We think that it is important to note that deployment challenges include both regulatory considerations and the fact that robust drive performance is still construct, locus, and species dependent. Our wording "in large part" emphasizes the technical aspects due to the nature of our manuscript, but does not exclude other considerations, which may play an increasing role in Anopheles in the near-future.

Figure 4B: Because the yellow system cross was done with female drive heterozygotes, can you cleanly distinguish somatic phenotype that resulted from maternal deposition vs somatic leaky expression? Looking back at Figure 2C, how did crosses have high embryonic resistance but low somatic level?

We apologize that the original manuscript did not describe clearly enough how the different drive performance parameters were measured in the *yellow* system. As noted above, we have added new Figure S2 in the revised manuscript to more clearly illustrate the phenotype classification and calculation of gene drive performance parameters in the *yellow* system.

Specifically, somatic leaky expression was assessed based on the body color of female drive heterozygotes generated by crossing Cas9 males to gRNA females. In this cross, Cas9 is inherited paternally rather than maternally, and therefore maternal deposition of Cas9 is not a confounding

factor. As a result, the body color changes observed in these female drive heterozygotes reflect somatic cleavage of *yellow* caused by somatic Cas9 expression.

By contrast, embryo resistance was measured in the progeny of these female drive heterozygotes crossed to wild-type males, and it was calculated as the proportion of yellow female progeny carrying the drive among female progeny carrying the drive, including only those in both categories that lacked Cas9. This shows resistance allele formation in the embryo that is associated with maternal deposition from the drive mother.

These two measurements therefore represent different biological processes occurring at different stages. Somatic phenotype in the female drive heterozygotes reflects leaky Cas9 somatic expression, whereas embryo resistance reflects events associated with maternal Cas9 into the next generation. Accordingly, a construct can show high embryo resistance but low somatic expression, because efficient maternal deposition into embryos does not necessarily imply strong detectable somatic leaky expression in the mother herself.

Line 543-544: I'm afraid this is the Achilles' heel of the study "but also, through integration with single-cell transcriptomic data,". The data indicates (see comment about Figure 3E) about half of the time, scRNA-seq results of endogenous gene don't match the expression pattern of Cas9 driven by the same gene promoter. And the difference is not just a difference in intensity, but huge differences like expressing in females when the endogenous gene is male-specific.

This issue is closely related to the comment raised above regarding Figure 3E (see our response for details). In sum, we would like to emphasize that our study does not assume that the endogenous scRNA-seq expression profile of a gene can fully recapitulate the pattern of Cas9 expression driven by a promoter fragment derived from that gene. Such difference is expected for several reasons, including the limited length of regulatory fragments, the integration site context, the use of median based Fly Cell Atlas data, and the fact that the atlas measures RNA abundance, whereas drive performance reflects an organism-level phenotype shaped by many processes.

Therefore, our intention was not to use single-cell transcriptomic data as a direct proxy for Cas9 expression patterns. Rather, we used these data in a comparative framework, asking whether genes associated with promoters linked to different drive performance tend to exhibit distinguishable cell type-specific expression patterns. In this sense, even if these data cannot perfectly predict promoter driven Cas9 activity, they can still provide biologically meaningful context and allow good promoter candidates to be identified substantially more quickly.

Minor comments:

Line 56: 'copy' rather than 'insert' would be more accurate here.
Line 69: typo: 'if many gRNAs'.

Line 87: an ideal homing gene drive.
Line 95: sufficient proportion of homozygous, therefore sterile…

All fixed (sometimes with more extensive rewording).

Line 99: one of the articles cited here (Hammond et al.) demonstrates large cage trials that succeeded in elimination cage population. Is this an intended citation here?

Whoops, this should have been the other Hammond *et al*. study from that year about promoters with different fitness costs. It has been fixed.

Line 108-110: the zpg strain in citation 41 was not found to exhibit fitness costs due to somatic expression, but due to paternal deposition.

No, this is only what the authors wrote about. The actual situation is that the fitness cost came from female heterozygotes due to new expression (probably somatic expression, though technically germline expression can't be ruled out), not from any parental deposition (though females may have given a very small amount of paternal deposition). We discuss this in a study that we now cite here, and the future studies from the Crisanti lab also shifted toward an explanation involving somatic expression rather than paternal deposition.

Line 114-115: all citations point to Aedes aegypti, what about other non-model species?

We revised the text and incorporated additional references from other species, including *Culex quinquefasciatus*, *Drosophila suzukii* and *Ceratitis capitata*, to better support our statement that promoter optimization remains challenging in non-model or less genetically tractable species.

Line 102-129: it is probably worth mentioning the CHIROS concept introduced by Ang et al. 2025 (https://doi.org/10.1016/j.cois.2025.101373) and the recently published single-nuclei atlas for Aedes aegypti (https://doi.org/10.1016/j.cell.2025.10.008) in these sections.
Line 126: it is not clear what the authors meant in 'what kinds of differences may be found between such data and Cas9 activity when driven by 126 promoters from native genes'.

We agree that these recent studies are relevant to this part. We have therefore revised the paragraph to incorporate this reference (and another with a nice figure) and the recently published single-nucleus atlas of *Aedes aegypti* and improve the clarity of our statements:
With the increasing availability of high-resolution transcriptomic resources, including the recently published single-nucleus atlas of *Aedes aegypti*, these datasets can potentially address this issue by providing precise information on expression patterns. Yet, it remains unclear exactly which expression pattern may be optimal, or to what extent expression patterns inferred from transcriptomic data reflect the actual Cas9 activity driven by promoters derived from native genes. It has been suggested that efficient homing may depend on Cas9 activity being restricted to specific

germline cell stages in which homology-directed repair is favored over end joining. Therefore, it is crucial to further investigate the relationship between expression of candidate promoter genes and drive performance to develop efficient and broadly applicable Cas9 promoter screening strategies for diverse species.

Figure 2B: it would be good either here or somewhere in the text to explain how conversion, germline resistance, and embryonic resistance rates are determined.

To address this concern, we have added new Figure S2 in the Supplementary Information, which provides a phenotype to parameter schematic for the *yellow* gene drive system.

Also for Figure 2C: Can you add a key to indicate which of these promoters are sex-specific, both sex, and constitutive?

We did seriously try to incorporate different symbols indicating specific expression patterns into the figure. However, Figure 2C already integrates a large amount of information and graphical elements within a limited space. Adding further icons and annotations (especially with borderline cases) for this and similar figures would require additional compression of the overall layout and font size, thereby reducing readability. Therefore, for this aspect, we would respectfully ask the reviewer and readers to refer also to Figure S1. To facilitate this, we have also added a note in the figure legend directing readers to Figure S1.

Also for 2C: Can you describe somewhere how you determine the somatic levels? What % is low and what % is high?

See new Figure S2 in the Supplementary Information.

Again for 2C: Which rhino construct is this?

We used the results from the RO3 construct. Because this construct contains the longest *rhino* promoter fragment and also uses the *rhino* 3′UTR, we believe that, among the three constructs, it most closely reflects the endogenous *rhino* expression pattern. We have also clarified this point in the legends of Figure 2C and Figure 3C.

Figure 3A: Does the lighter shade in the gRNA mean something?

In the gRNA expression cassette, the darker region was originally supposed to represent tRNA regions or a “terminator” at the end, but these were mixed up when making the figures. It has been fixed. It’s been removed for the single gRNA constructs and slightly revised for the dsx construct in the supplemental figure.

Figure 4: It will be helpful to remind readers in the legend that this is derived from the yellow drive system. For consistency, I'd suggest changing 'high and low' to 'low and high' in the legend.

We have changed the legend to "Differential cell-type expression analysis of promoter-associated genes for low and high embryo resistance and somatic activity in the *yellow* drive system." We have also clarified this point in the opening sentence of the relevant section that these measurements were derived from the *yellow* drive system.

Line 436-438: I could be missing something here but my understanding from Figure 4B is the opposite i.e. high expression was positively correlated with somatic expression. This also contradicts lines 641-642 of the manuscript.

Good catch! It has been fixed (the text in lines 641-642 is correct).

Line 457-461: It is not clear how these two cell types are singled out in the analyses in Figure 5A-B).

These two cell types emerged from our earlier cell type differential expression analyses for female drive conversion and embryo resistance. As described in the manuscript, we found that female drive conversion was associated with high Cas9 expression in certain female germline cell types, whereas low embryo resistance allele formation was associated with low Cas9 expression in those same cell types. Because both of these drive performance parameters were linked to changes in the same two cell types, we highlighted them in Fig. 5A-B. We now explicitly mention this.

Line 486: typo (previously)

Fixed.

Line 464: It would be good to mention which three kinds in the text even if it is mentioned in Figure 5.

We have added this. The revised sentence is shown below:
To further assess this, we examined the expression profiles of genes corresponding to three kinds of promoters with distinct drive performance: high female conversion-high embryo resistance, high female conversion-low embryo resistance, and low female conversion-low embryo resistance, focusing on their expression in female germline cells.

Line 466-467: It is not clear which part of the results in Figure 5C lead to the conclusion made in these two lines. Is it the fact that the green data points are all quite different to the orange data points? But if that was the case, there is almost no difference between the green and purple data points, the latter has low conversion and low embryo resistance.

We agree that the three groups are not completely separated in Figure 5C, and that there is indeed some degree of overlap. However, there is still a clear pattern. Purple is almost always lower than green in both cell types, which is in turn almost always lower than orange. This is most emphasized with the orange points that are much higher, but it can be seen even when zoomed in, allowing greater emphasis on differences that are smaller in magnitude (but still substantial in terms of relative difference).

These differences are not crystal clear, though, even when zooming even. We have thus toned down our wording when describing this result.

Line 455-497: I think this section quite hard to follow and may need some rewriting for better clarity. At the moment it has many ideas/results but not enough context to enable a good flow of the idea.

Besides the above, we've made some additional revisions to these two paragraphs (separating them to include a third paragraph) that will hopefully provide clearer explanations.

Line 489-490: What about egg-to-pupa survival rate of the progeny? Did you measure them?

We did not further examine the egg-to-pupa survival rate. This could be influenced by embryo resistance and somatic expression, which would have been a complicated analysis and not addressed the main issue (the influence of somatic activity is very different from the analysis). If we had high female germline cut rate, then an investigation of embryo resistance would still have been appropriate and potentially have shown a difference. However, given the low female activity, we expect hat there would be no detectable embryo resistance.

Line 496-497: Could the multi-sgRNA in Rpl35A have anything to do with this?

We agree that the multi-sgRNA design in the *RpL35A* system could be indirectly related to this outcome, although we did not directly test its specific contribution here. The use of multiple gRNAs is expected to reduce the probability of generating functional resistance alleles while increasing the formation of nonfunctional resistance alleles. Because *RpL35A* is a haplolethal target, such nonfunctional resistant alleles are eliminated. We used the tRNA system here instead of individual promoters for gRNAs. Our other studies, some ongoing, indicate that this (and ribozymes) may be a bad idea for preserving gRNA activity. We got away with it before with the RpL35A construct with strong Cas9 and gRNA promoters, allowing retention of high germline cutting. Our future studies will explore gRNA expression in more detail.

Line 517-518: Can you comment on why rhino was picked for this and not other promoters?

We chose *rhino* because it represented a germline-specific promoter with detectable drive activity and substantial room for improvement. In particular, it showed measurable cutting efficiency, relatively high embryo resistance and leaky somatic expression, making it a useful case for evaluating whether further construct optimization could improve drive performance. We have added a brief statement in the revised manuscript to clarify this rationale. Additionally, it was chosen for this before most of our other tests were completed and with less complete data (the *rhino* studies were complete well before much of the rest of the manuscript, even though it fit best at the end when we were writing it).

Line 574-575: Don't think I can agree with this.

We believe this point can be addressed by referring to our earlier response regarding Figure 3E and Lines 543-544. In particular, the permutation test provides support for a relationship between promoter activity and the transcriptomic patterns of the corresponding genes, indicating that the analysis retains biological relevance. Regarding the specific language, we agree that genomic context is important, but we are specifically referring the 5′ regulatory elements here. We have softened our language on this point in the manuscript.

Line 606-608: One successful example would be the nanosD strain in An. gambiae (https://academic.oup.com/g3journal/advance-article/doi/10.1093/g3journal/jkaf246/8305671?searchresult=1).

We would first like to clarify what we meant in the manuscript when referring specifically to suppression drives. Suppression drives typically target a gene that is important for fertility, development, or survival. If one attempts to use the native regulatory elements of such a gene to drive Cas9 expression, then once Cas9 is expressed, it will cleave the target essential gene. As a result, in a drive/wild-type heterozygote, the functional wild-type copy may be cleaved and mutated, while the drive allele, due to the insertion of the drive element, will fully disrupt the target gene. Consequently, before the individual can realize the intended transmission advantage of the drive, it may instead suffer a fitness cost, sterility, or even embryonic or organismal lethality because the function of this critical gene has been compromised in some cells where drive activity occurs. If Cas9 cleavage occurs sufficiently late, however, the endogenous gene may already have been transcribed and translated to produce a sufficient amount of normal gene product before the locus is disrupted. In that case, even if the locus is subsequently cleaved, the gene product accumulated earlier may still be sufficient to support normal development or reproductive function.

We agree that the *nanos*D strain provides a case in which an integral gene drive exploits an endogenous locus and its native regulatory context. We have therefore cited this study where we discuss integral drives elsewhere in the manuscript. However, this system was a modification drive, not a suppression drive as we describe above.

In the following, reviewer comments are in bold, and our responses are in plain text.

Reviewer #1 (Remarks to the Author):

The amended manuscript is improved. Figure s2 helps significantly. I find the term 'embryo resistant rate' confusing but once I have understood it is a proportion of female flies without the cas9 gene that destroy yellow (Ry) out of the total female flies without cas9 then it makes some sense. It is not clear to me why mosaics (Rm) don't count to the numerator.

First, we would like to clarify our definition of embryo resistance. In this study, embryo resistance refers to the formation of uncleavable resistant alleles at the wild-type *yellow* locus (located on the X chromosome) due to maternally deposited Cas9 in the absence of a genomic Cas9 source. In the absence of embryo and somatic cleavage, female individuals carrying the dsRed but lacking Cas9 should be heterozygous at the *yellow* locus, with the allele containing dsRed inherited maternally and the wild-type *yellow* allele inherited paternally. The paternally derived yellow allele may subsequently be cleaved by maternally deposited Cas9, resulting in the formation of a resistance allele.

In theory, the appearance of the mosaic phenotype (in individuals without Cas9) indicates that maternally deposited Cas9 cleaved the *yellow* gene in at least a subset of embryonic cells. However, results from our previously published study (Champer et al., 2019, *Molecular safeguarding of CRISPR gene drive experiments*) demonstrated that mosaic yellow females could still mediate germline drive conversion, with 15 of 16 mosaic drive carrier females showing an average conversion efficiency of 54%. These findings suggest that, in mosaic yellow females, only a small fraction of the cleavage events induced by deposited Cas9 occur directly within the germline lineage. Consequently, the majority of germline cells in these individuals still retain targetable wild-type *yellow* alleles capable of undergoing drive conversion. Therefore, we did not include Rm individuals in the calculation of embryo resistance. This mosaic phenotype still represents important events, but it is distinct from the complete phenotype, and often closely correlated for embryo resistance.

Additionally I suppose heterozygotes for dsRED cannot be distinguished from homozygotes at this stage?

We are not quite sure where the reviewer is going with this question, but we can provide a detailed answer. It is possible to distinguish fluorescent markers between homozygotes and heterozygotes, and we often use this to generate homozygous lines more quickly. However, it is a highly error-prone process when using the naked eye + microscope because there is a fair amount of natural fluorescence variation between individuals (which is often affected by age). In our experience, this can be overcome by using imaging and computer-based methods. However, it might be complicated in cases with high somatic activity, which results in a moderate about of drive conversion in somatic cells (thus giving an intensity between homozygotes and true heterozygotes).

I also note that snRNA-Seq reflects an "On" switch, but manipulating the "Off" switch that determines transcript and protein half life might need similar scrutiny in the future.

We agree that the mechanisms controlling transcript and protein degradation, stability, and half-life represent an important direction for future investigation. Transcript half-life is also essential, of course, but this is largely already captured by RNA sequencing methods (lower half-life will result in faster degradation and thus a lower amount of transcript detected at any particular time, which is what is directly measured). Regarding protein stability, only a little work has been done, and there is certainly room for improvement (our previous test with PEST sequences went to far toward destabilizing Cas9).

We have now expanded the Discussion to highlight this point:
"Beyond regulation at the promoter and UTR levels, protein stability may also influence Cas9 activity and drive performance. Previous work using PEST sequences to modulate Cas9 protein stability suggests that post-translational regulation can substantially affect effective Cas9 activity[37,62]. Determining protein turnover rates would provide a more complete understanding of how Cas9 expression is regulated after transcription and translation in the future."
